# Glycosylation of Methoxylated Flavonoids in the Cultures of *Isaria fumosorosea* KCH J2

**DOI:** 10.3390/molecules23102578

**Published:** 2018-10-09

**Authors:** Monika Dymarska, Tomasz Janeczko, Edyta Kostrzewa-Susłow

**Affiliations:** Department of Chemistry, Wrocław University of Environmental and Life Sciences, Norwida 25, Wrocław 50-375, Poland; janeczko13@interia.pl (T.J.); ekostrzew@gmail.com (E.K.-S.)

**Keywords:** flavonoids, biotransformations, *Isaria*, fungi, flavonoid glycosides, 4-*O*-methylglucopyranoside, methoxyflavanone, methoxyflavone

## Abstract

Flavonoids are widely described plant secondary metabolites with high and diverse pro-health properties. In nature, they occur mostly in the form of glycosides. Our research showed that an excellent way to obtain the sugar derivatives of flavonoids is through biotransformations with the use of entomopathogenic filamentous fungi as biocatalysts. In the current paper, we described the biotransformations of five methoxylated flavonoid compounds (2′-methoxyflavanone, 3′-methoxyflavanone, 4′-methoxyflavanone, 6-methoxyflavanone, and 6-methoxyflavone) in cultures of *Isaria fumosorosea* KCH J2. As a result, we obtained twelve new flavonoid 4-*O*-methylglucopyranosides. The products were purified with methods that enabled the reduction of the consumption of organic solvents (preparative TLC and flash chromatography). The structures of the products were confirmed with spectroscopic methods (NMR: ^1^H, ^13^C, HSQC, HMBC, COSY). The compounds obtained by us expand the library of available flavonoid derivatives and can be used in biological research.

## 1. Introduction

Nowadays preventive medicine is increasing in importance. It is estimated that all over the world, the cost of chronic diseases in 2015–2030 will reach $17 trillion (including health care costs and reduced productivity). A sub-optimal diet is currently the leading risk factor for both disabilities and death worldwide [1]. The prevention and control of non-communicable diseases (such as cancer, cardiovascular diseases, and diabetes) will be one of the major challenges in healthcare in the next 50 years [2].

It is well known that vegetable and fruit consumption may be associated with lower mortality and non-communicable diseases rates [2]. Health professionals claim >50% of health problems can be prevented by implementing an appropriate diet [1]. The 2015–2020 Dietary Guidelines for Americans strongly recommend the intake of more vegetables and fruits in our daily diet [3]. Just consuming 1–2 servings of fruit rich in anthocyanins (such as strawberries, raspberries or blueberries) every day helps significantly reducing the risk of cardiovascular diseases [1]. In vitro and in vivo studies confirm that flavonoid compounds are desirable from the pharmaceutical point of view activities: they are anti-oxidants, anti-inflammatory, vasorelaxants, anticoagulants, cardioprotective, anti-obesity and anti-diabetic, chemoprotective, neuroprotective, antidepressants, antimicrobials [3,4,5,6,7,8].

Many researchers argue that the presence of free hydroxyl groups is crucial for the biological activity of flavonoid compounds [9,10,11]. However, some studies conclude that flavonoid derivatives containing methoxy groups or sugar units in their structure also show desirable biological potential.

Polymethoxyflavones are one of the types of flavonoid compounds found in significant amounts in citrus. Those polyphenols may regulate metabolic disorders, prevent cardiovascular diseases, protect neurons and have some other desirable bioactivities such as anti-atherosclerotic, anti-inflammatory, neuroprotective, anti-cancer, anti-microbial, and anti-oxidative effects [12,13]. Nobiletin and tangeretin (A-ring polymethoxylated flavonoids possessing methoxyl groups in the B ring as wll) demonstrated high anti-proliferative activity in cancer cells. In thecase of chrysin, methoxylation greatly enhanced the anti-inflammatory properties. Akbar et al. demonstrated that 6-methoxyflavanone (**4**) has the potential to easily cross the blood-brain barrier and possesses anti-allodynic and anti-vulvodynic activities in the rat model of streptozotocin-induced diabetes mellitus. Therefore, this compound could be used to treat neuropathic pain associated with diabetes mellitus [14]. In another study, Akbar et al. showed that 6-methoxyflavanone (**4**) exerted an anxiolytic-like effect in several tests conducted with mice [15]. *O*-methylation has been proven to be also beneficial to the anti-viral, anti-bacterial, and anti-diabetic properties of flavonoids [8].

Despite the extraordinary biological activities of flavonoids, these compounds are still relatively rarely used as pharmaceuticals. This is due to their poor bioavailability [8,16]. Glycosylation is a well-known method of improving the properties of flavonoid compounds, such as solubility and stability [3,6,17]. Flavonoid glycosides often present a lower bioavailability than their parent aglycones but not in the case of glucosides. The presence of a glucose unit attached to the flavonoid skeleton is reported to improve its absorption [17,18]. In most cases, prior to absorption, flavonoid glycosides need to be hydrolyzed into aglycones. Anthocyanins are a well-documented exception: intact glycosides were detected in the circulation system [16,17,19]. Another relatively high bioavailable group are methoxylated flavonoids. When compared to hydroxyl flavonoids, a 100-fold higher plasma concentration has been obtained for methoxylated derivatives [8]. The compounds 5,7-dimethoxyflavone and 3′,4′-dimethoxyflavone were reported to be more stable compared to galangin (3,5,7-trihydroxyflavone) [16].

Bioavailability and biological activities of compounds strongly differ between distinct flavonoid subclasses, and within subclasses, they depend on the substitution patterns. For this reason, it is important to expand the flavonoid library with new derivatives, doing so gives us tools to further understanding the metabolism of this complex group of plant secondary metabolites in humans and may result in obtaining new pharmaceuticals of natural origin.

Our study is a continuation of research on the biotransformations of flavonoid aglycones in cultures of entomopathogenic filamentous fungi [20,21,22]. In the current paper, we describe the biotransformations of six methoxylated flavonoid compounds (2′-methoxyflavanone, 3′-methoxyflavanone, 4′-methoxyflavanone, 6-methoxyflavanone, and 6-methoxyflavone) in cultures of *Isaria fumosorosea* KCH J2. As a result, we obtained twelve flavonoid 4-*O*-methylglucopyranosides that were not previously described. Our current research provides an insight into the microbial glycosylation of flavonoid compounds lacking free hydroxyl groups. The biotransformation products expand the library of available flavonoid derivatives and can be used in biological research.

## 2. Results and Discussion

In order to obtain new flavonoid 4-*O*-methylglucopyranosides, we used five flavonoid substrates and a strain of the entomopathogenic filamentous fungus isolated from spider carcasses in Wrocław: *Isaria fumosorosea* KCHJ2, which is so far the most efficient biocatalyst in the microbial glycosidation of flavonoids in our collection. Detailed characteristics of the strain can be found in our previous publication [20].

For the basis of the screening, we chose different durations of scale-up biotransformations for each substrate. Each scale-up biotransformation was terminated on the day when, in the HPLC analysis of a sample taken during the screening procedure, no peak was observed from the substrate, and the areas under the peaks from the putative glycosylation products were the highest. Experiments conducted on a larger scale allowed us to determine the chemical structures of products and their isolated yields. Percent yields are given in relation to the molar ratio of products to substrates. Enantiomeric excesses of products and unreacted substrates were determined by HPLC using chiral column; we did not observe any significant enantiomeric excesses. In the course of our study, we obtained twelve biotransformation products, none of the products have been previously reported in the literature.

### 2.1. Biotransformations of 2′-Methoxyflavanone (1)

**Scheme 1:**
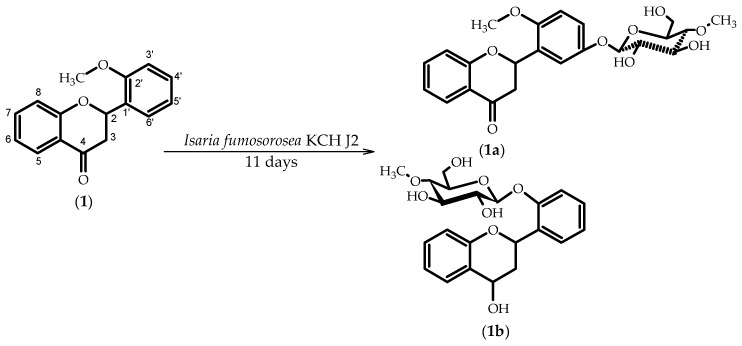
The microbial transformation of 2′-methoxyflavanone (**1**) in the *I. fumosorosea* KCH J2 culture.

As a result of the 11-day biotransformation of 2′-methoxyflavanone (**1**) in the culture of *I. fumosorosea* KCH J2, we obtained two products: 2′-methoxyflavanone 5′-*O*-β-D-(4″-*O*-methyl)-glucopyranoside (**1a**) with an 18.6% yield and flavan-4-ol 2′-*O*-β-d-(4″-*O*-methyl)-glucopyranoside (**1b**) with a 24.9% yield (Scheme 1).

The presence of a glucose unit in molecules **1a** and **1b** is confirmed by five characteristic carbon signals observed in the region from about δ = 80.0 ppm to about δ = 62.0 ppm in the ^13^C-NMR spectra, along with the proton signals of δH ranging from about δ = 3.85 ppm to δ = 3.23 (3.28) ppm in the ^1^H-NMR spectra. Additionally, the attachment of a sugar unit to substrate 1 was confirmed by a one-proton doublet visible at δ = 4.90 ppm in the ^1^H-NMR spectrum of **1a** and δ = 4.85 ppm in the ^1^H-NMR spectrum of 1b, which are due to protons at the anomeric carbon atoms. The β-configuration of the glucose unit was proved by the coupling constant (*J* = 7.8 Hz) for the anomeric proton. A three-proton singlet at about δ = 3.60 ppm in the ^1^H-NMR and the corresponding signal at about δ = 60.5 ppm in the ^13^C-NMR proved that one of the hydroxyl groups had been methylated. *O*-methylation occurred in the C-4″ hydroxyl group of the glucose moieties, which was confirmed in the HMBC spectra, where the proton signals due to -OCH_3_ were coupled with the signal of C-4 (δ = 80.2/79.8 ppm) in the glucose unit.

The sugar unit was attached to C-5′ of **1a** because, in the HMBC spectrum, the signal due to the proton at the anomeric carbon atom (δ = 4.90 ppm) was coupled with the C-5′ signal (δ = 152.8 ppm), which was shifted from δ = 121.6 ppm, indicating the attachment of an electronegative atom. In the COSY spectrum, it can be seen that the proton at C-6′ became isolated. The ^13^C-NMR signals from C-4′ and C-6′ were shifted towards higher field values, indicating the appearance of an electron-withdrawing substituent in their vicinity. Chemical shifts of the other signals in the ^1^H- and ^13^C-NMR spectra have only slightly changed, which indicates that the flavanone skeleton remained intact.

In the case of compound **1b**, the sugar unit was attached to C-2′, because in the HMBC spectrum the signal due to the proton at the anomeric carbon atom (δ = 4.85 ppm) was coupled with the C-2′ signal (δ = 155.4 ppm), which was only slightly shifted from δ = 157.0 ppm. The positions of the signals from the remaining protons of the B-ring did not significantly change, indicating no changes in the substitution pattern. In the C ring, the carbonyl group at C-4 was reduced. The proof is the lack of a characteristic signal at about δ = 190 ppm on the ^13^C-NMR spectrum and the appearance of a signal at δ = 64.3 ppm along with one-proton singlet at δ = 4.83 ppm observed in the ^1^H-NMR spectrum of **1b**.

### 2.2. Biotransformations of 3′-Methoxyflavanone (2)

**Scheme 2:**
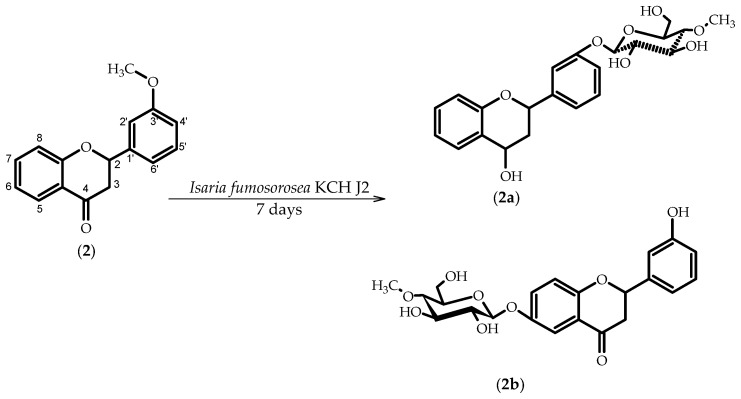
The microbial transformation of 3′-methoxyflavanone (**2**) in *I. fumosorosea* KCH J2 culture.

As a result of the 7-day biotransformation of 3′-methoxyflavanone (**2**) in the culture of *I. fumosorosea* KCH J2, we obtained flavan-4-ol 3′-*O*-β-d-(4″-*O*-methyl)-glucopyranoside (**2a**) with a 15.4% yield and 3′-hydroxyflavanone 6-*O*-β-d-(4″-*O*-methyl)-glucopyranoside (**2b**) with a 16% yield (Scheme 2).

The presence of a glucose unit in molecules **2a** and **2b** was confirmed by five characteristic carbon signals observed in the region from about δ = 80.0 ppm to δ = 62.0 ppm in the ^13^C-NMR spectra, along with the proton signals of δH ranging from about δ = 3.90 ppm to δ = 3.30 ppm in the ^1^H-NMR spectra. The attachment of a sugar unit to substrate 2 was also confirmed by a one-proton doublet visible at δ = 5.03/4.93 ppm in the ^1^H-NMR spectrum of **2a**/**2b**, which was due to the protons at the anomeric carbon atoms. The β-configuration of the glucose unit was proved by the coupling constant (*J* = 7.8 Hz) for the anomeric proton. A three-proton singlet at δ = 3.60 ppm in the ^1^H-NMR spectra and the corresponding signal at δ = 60.5 ppm in the ^13^C-NMR spectra indicates the *O*-methylation of the hydroxyl group at C-4″ of glucose, which was confirmed on the basis of the analysis of the HMBC spectra of compound **2a**/**2b**, where the proton signals due to -OCH_3_ (δ = 3.60 ppm) were coupled with the signal of C-4″ (δ = 80.1/79.9 ppm) in the glucose unit.

The sugar unit was attached to C-3′ of **2a** because in the HMBC spectrum the signal due to the proton at the anomeric carbon atom (δ = 5.03 ppm) was coupled with the C-3′ signal (δ = 158.9 ppm) which was only slightly shifted from δ = 160.9 ppm. There were no significant changes in the chemical shifts of signals of the remaining protons in the B-ring indicating no changes in the substitution pattern. In the ^13^C-NMR of **2a**, there is no characteristic signal at about δ = 190 ppm, suggesting a change in substitution at C-4. The appearance of a signal at δ = 63.6 ppm along with one-proton signal at δ = 4.81 ppm in the ^1^H-NMR of **2a** prove that the carbonyl group at C-4 was reduced.

In the case of **2b**, the glucose moiety was attached to C-6, which was proved on the basis of the HMBC spectrum. The signal due to the proton at the anomeric carbon atom (δ = 4.93 ppm) was coupled with the C-6 signal (δ = 153.1 ppm), which was shifted from δ = 122.3 ppm. There was no signal from C-6 in the ^1^H-NMR spectrum of **2b**. The ^13^C-NMR spectrum signals from C-5 and C-7 were shifted towards higher field values (upfield), suggesting the appearance of a strongly electronegative substituent in their vicinity. Demethylation at C-3′ was confirmed on the basis of the HMBC spectrum of **2b**: the signal from C-3′ (δ = 158.5 ppm) was coupled with a singlet signal from the hydroxyl group proton (δ = 8.54 ppm) and there was also no signal from C-3′-OCH_3_ in the ^1^H-NMR.

### 2.3. Biotransformations of 4′-Methoxyflavanone (3)

**Scheme 3:**
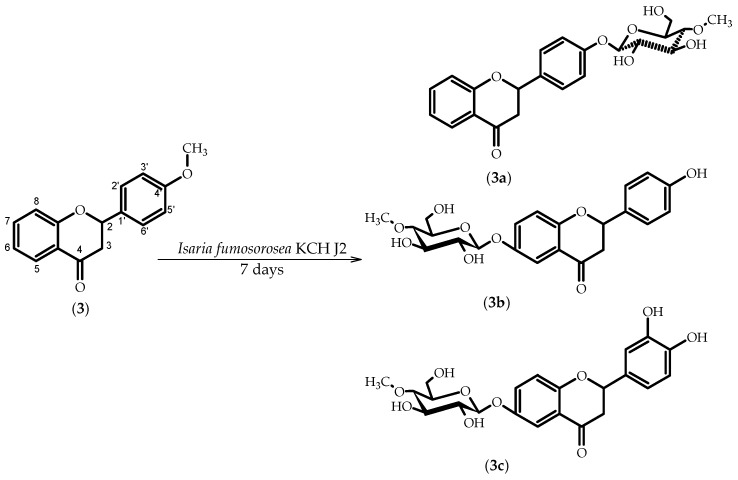
The microbial transformation of 4′-methoxyflavanone (**3**) in *I. fumosorosea* KCH J2 culture.

As a result of the 7-day biotransformation of 4′-methoxyflavanone (**3**) in the culture of *I. fumosorosea* KCH J2, we obtained flavanone 4′-*O*-β-d-(4″-*O*-methyl)-glucopyranoside (**3a**) with a 11% yield, 4′-hydroxyflavanone 6-*O*-β-d-(4″-*O*-methyl)-glucopyranoside (**3b**) with a 20.7% yield and 3′,4′-dihydroxyflavanone 6-*O*-β-_D_-(4″-*O*-methyl)-glucopyranoside (**3c**) with a 32.8% yield (Scheme 3).

The presence of a glucose unit in molecules **3a**, **3b**, and **3c** was confirmed by five characteristic carbon signals observed in the region from about δ = 80.0 ppm to δ = 62.0 ppm in the ^13^C-NMR spectra and proton signals from about δ = 3.87 ppm to about δ = 3.27 ppm in the ^1^H-NMR spectra. Additionally, the attachment of a sugar unit to substrate 3 was confirmed by a one-proton doublet at δ = 5.02/4.93/4.92 ppm in the ^1^H-NMR spectrum of **3a**/**3b**/**3c**, which was due to the protons at the anomeric carbon atoms. The β-configuration of the glucose unit was proved by the coupling constant (*J* = 7.8/7.7/6.3 Hz) for the anomeric proton. A three-proton singlet at δ = 3.60 ppm in the ^1^H-NMR and the corresponding signal at about δ = 60.5 ppm in the ^13^C-NMR prove that one of the hydroxyl groups had been methylated. *O*-methylation occurred in the C-4″ hydroxyl group of the glucose moiety, which was confirmed in the HMBC spectra of the biotransformation products **3a**/**3b**/**3c**, where the proton signals due to -OCH_3_ (δ = 3.60 ppm) were coupled with the signal of C-4″ (δ = 80.1/79.9/79.9 ppm) in the glucose unit.

The sugar unit was attached to C-4′ of compound **3a** because in the HMBC spectrum the signal due to the proton at the anomeric carbon atom (δ = 5.02 ppm) was coupled with the C-4′ signal (δ = 158.9 ppm) which was only slightly shifted from δ = 160.9 ppm. There were no other changes in the substitution pattern of the flavanone skeleton because chemical shifts of the other signals in the ^1^H and ^13^C-NMR spectra had only slightly changed.

In the case of compound **3b** and **3c**, demethylation at C-4′ occurred. In the HMBC spectrum of the **3b** signal from C-4′ (δ = 158.6 ppm) was coupled with a singlet signal from the hydroxyl group proton (δ = 8.57 ppm); there was also no signal from C-4′-OCH_3_ in the ^1^H-NMR or ^13^C-NMR of **3b** and **3c**. The glucose unit was attached to C-6 because in the HMBC spectrum of **3b** (**3c**) the signal due to the proton at the anomeric carbon atom (δ = 4.93/4.92 ppm) was coupled with the C-6 signal (δ = 153.0 ppm), which was shifted from δ = 122.1 ppm. The lack of a signal from C-6 in the ^1^H-NMR spectrum of **3b** and the shift of signals from C-5 and C-7 towards higher field values in the ^13^C-NMR spectrum indicate the attachment of a sugar at C-6. In the case of compound **3c**, an additional hydroxylation occurred in position C-3′, because the ^13^C-NMR signal from C-3′ was visible at δ = 146.0 ppm indicating the attachment of a strongly electronegative atom. In the COSY spectrum, the signal from C-2′ became isolated and was shifted towards higher field values in the ^13^C-NMR.

### 2.4. Biotransformations of 6-Methoxyflavanone (4)

**Scheme 4:**
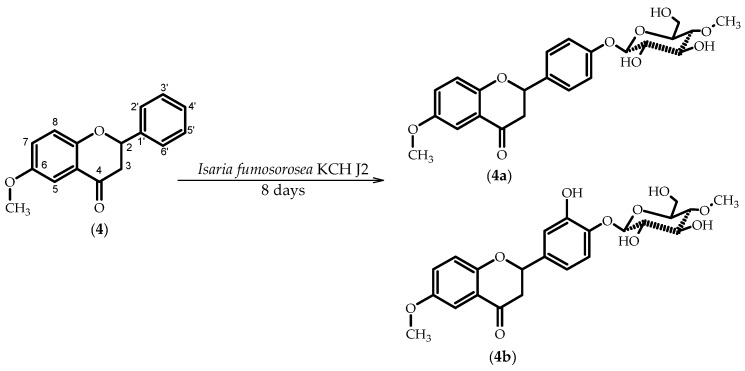
The microbial transformation of 6-methoxyflavanone (**4**) in *I. fumosorosea* KCH J2 culture.

As a result of the 8-day biotransformation of 6-methoxyflavanone (**4**) in the culture of *I. fumosorosea* KCH J2, we obtained 6-methoxyflavanone 4′-*O*-β-d-(4″-*O*-methyl)-glucopyranoside (**4a**) with a 6% yield and 3′-hydroxy-6-methoxyflavanone 4′-*O*-β-d-(4″-*O*-methyl)-glucopyranoside (**4b**) with a 7% yield (Scheme 4).

The presence of a glucose unit in molecule **4a** (**4b**) was confirmed by five characteristic carbon signals observed in the region from about δ = 80.0 ppm to about δ = 62.0 ppm in the ^13^C-NMR spectra, along with proton signals of δH ranging from about δ = 3.90 ppm to δ = 3.26 ppm in the ^1^H-NMR spectra. Additionally, the attachment of a sugar unit to substrate 4 was confirmed by a one-proton doublet at δ = 5.01 (4.81) ppm in the ^1^H-NMR spectrum of **4a** (**4b**) from the protons at the anomeric carbon atoms. The β-configuration of the glucose unit was proved by the coupling constant (*J* = 7.8/7.9 Hz) for the anomeric proton. A three-proton singlet at δ = 3.60 ppm in the ^1^H-NMR and the corresponding signal at δ = 60.6 ppm in the ^13^C-NMR proved that one of the hydroxyl groups was methylated. *O*-methylation occurred in the C-4″ hydroxyl group of the glucose moiety, which was confirmed in the HMBC spectra of compound **4a** (**4b**), where the proton signals due to -OCH_3_ (δ = 3.60 ppm) were coupled with the signal of C-4″ (δ = 80.1/80.0 ppm) in the glucose unit.

In the case of **4a**, a sugar unit was attached to C-4′, it was proved in the HMBC spectrum. The signal due to the proton at the anomeric carbon atom (δ = 5.01 ppm) was coupled with the C-4′ signal (δ = 158.9 ppm), which was shifted from δ = 129.3 ppm indicating a substitution with a strongly electronegative atom. The substitution in position 4′ was also evidenced by the characteristic two doublets in the ^1^H-NMR spectrum from the protons at C-2′ and C-6′ (δ = 7.54 ppm) as well as C-3′ and C-5′ (δ = 7.15 ppm).

In the case of **4b**, 4-*O*-methylglucopyranose was also attached to C-4′. In the HMBC spectrum of **4b**, the signal due to the proton at the anomeric carbon atom (δ = 4.81 ppm) was coupled with the C-4′ signal (δ = 146.2 ppm). Additionally, hydroxylation into position C-3′ occurred. In the ^13^C-NMR spectrum, the signal from C-3′ was shifted toward a lower field value (from δ = 129.5 ppm to δ = 148.8 ppm) and the signals from C-2′ and C-5′ were shifted upfield by 12.3 ppm and 10.9 ppm, respectively, indicating the appearance of strongly electronegative atoms in their vicinity. In the ^1^H-NMR spectrum, there is no signal from the protons at C-3′ and C-4′. The substitution pattern was also confirmed on the basis of the COSY spectrum in which it can be seen that signals from C-2′ became isolated.

### 2.5. Biotransformations of 6-Methoxyflavone (5)

**Scheme 5:**
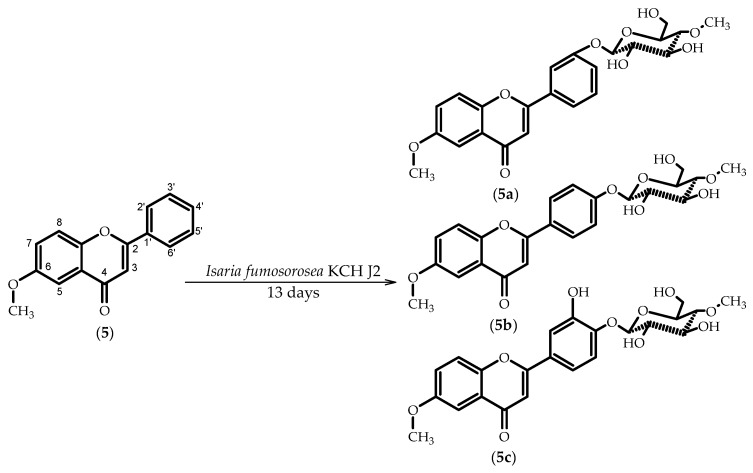
The microbial transformation of 6-methoxyflavone (**5**) in *I. fumosorosea* KCH J2 culture.

As a result of the 12-day biotransformation of 6-methoxyflavone (**5**) in the culture of *I. fumosorosea* KCH J2 we obtained 6-methoxyflavone 3′-*O*-β-d-(4″-*O*-methyl)-glucopyranoside (**5a**) with a 2.6% yield, 6-methoxyflavone 4′-*O*-β-d-(4″-*O*-methyl)-glucopyranoside (**5b**) with a 7.8% yield and 3′-hydroxy-6-methoxyflavone 4′-*O*-β-d-(4″-*O*-methyl)-glucopyranoside (**5c**) with a 7.9% yield (Scheme 5).

The presence of a glucose unit in molecule **5a** (**5b**, **5c**) was confirmed by five characteristic carbon signals observed in the region from about δ = 80.0 ppm to about δ = 62.0 ppm in the ^13^C-NMR spectra, along with proton signals of δH ranging from about δ = 3.94 ppm (3.90 ppm, 3.92 ppm) to δ = 3.25 ppm (3.28 ppm, 3.29 ppm) in the ^1^H-NMR spectra. Additionally, the attachment of a sugar unit to substrate 5 was confirmed by a one-proton doublet visible at δ = 5.14 ppm (5.14 ppm, 5.00 ppm) in the ^1^H-NMR spectrum of **5a** (**5b**, **5c**) from the protons at the anomeric carbon atoms. The β-configuration of the glucose unit was proved by the coupling constant (*J* = 7.8 Hz) for the anomeric protons. A three-proton singlet at about δ = 3.60 ppm in the ^1^H-NMR and the corresponding signal at δ = 60.6 ppm in the ^13^C-NMR proved that one of the hydroxyl groups had been methylated. *O*-methylation occurred in the C-4″ hydroxyl group of the glucose moiety, which was confirmed in the HMBC spectra of compound **5a** (**5b**, **5c**), where the proton signals from-OCH_3_ were coupled with the signal of C-4″ (δ = 80.3 ppm, 80.1 ppm, 80.0 ppm) in the glucose molecule.

The sugar unit was attached to C-3′ of **5a** because in the HMBC spectrum, the signal due to the proton at the anomeric carbon atom (δ = 5.14 ppm) was coupled with the C-3′ signal (δ = 159.3 ppm). The ^13^C-NMR signals from C-2′ and C-4′ were shifted upfield indicating the appearance of an electron-withdrawing substituent in their vicinity. In the COSY spectrum, it could be seen that the signal from C-2′ became isolated.

In the case of compounds **5b** and **5c**, the sugar unit was attached to C-4′. In the HMBC spectra, signals due to the protons at the anomeric carbon atoms (δ = 5.14 ppm, 5.00 ppm) were coupled with signals from C-4′ (δ = 161.3 ppm, δ = 148.9 ppm) which were shifted downfield from δ = 132.4 ppm. In the case of **5b**, the characteristic two two-proton signals in the ^1^H-NMR spectrum from the protons at C-2′ and C-6′ (δ = 8.08 ppm) as well as C-3′ and C-5′ (δ = 7.28 ppm) indicated the substitution at C-4′. The compound 5c has also hydroxyl group at C-3′, in the ^13^C-NMR signal from C-3′ was shifted downfield by 18.8 ppm and the signal from C-2′ was shifted upfield by 12.6 ppm indicating the attachment of strongly electronegative atom to C-3′. In the COSY spectrum, the signal from C-2′ became isolated.

In the available literature, we have not found any reports on obtaining sugar derivatives of substrates, whose biotransformations we describe in the present paper. The only two examples of the microbial glycosylation of flavonoid compounds lacking hydroxyl groups are described by us as the biotransformations of 6-methylflavone [20] and flavone [21] in cultures of entomopathogenic filamentous fungi. In the case of 6-methylflavone, 4-O-methylglucopyranose was attached to C-8 in the first product and to C-4′ in the second product. The biotransformation of flavone yielded three products: flavone 2′-*O*-β-d-(4″-O-methyl)-glucopyranoside, flavone 4′-*O*-β-d-(4″-*O*-methyl)-glucopyranoside and 3′-hydroxyflavone 4′-*O*-β-d-(4″-*O*-methyl)-glucopyranoside.

For microbial transformations of methoxy derivatives of flavonoids, the most frequently described reaction is demethylation. Our team reported on the enantioselective conversion of four derivatives of 6-hydroxyflavanone. Racemic 6-methoxyflavanone was hydrolyzed by filamentous fungus Aspergillus niger MB to 6-hydroxyflavanone. The R-enantiomer of 6-hydroxyflavanone was further hydroxylated at the C-4′ position, while the S-enantiomer was biotransformed into 6-hydroxyflavone [23]. In another publication, *Kostrzewa-Susłow* et al. described the biotransformations of 6- and 7-methoxyflavones in cultures of A. niger and Penicillium chermesinum. In the A. niger MB culture, 6-methoxyflavone underwent hydrolysis to 6-hydroxyflavone which was further biotransformed into 4′,6-dihydroxyflavone [24]. Cao et al. in an extensive review paper described examples of various microbial transformations of bioactive flavonoids. In the case of tangeretin (4′,5,6,7,8-pentamethoxyflavone), A. niger ATCC 9142 selectively O-demethylated the methoxy group at the C-4′ position. A. alliaceus UI 315 performed the hydrolysis of 2′,3′-dimethoxyflavanone to 3′-hydroxy-2′-methoxyflavanone. Cuninghamella elegans NRRL 1392 performed the O-demethylation of 7-*O*-methylnaringenin (4′,5-dihydroxy-7-methoxyflavanone) and 4′,5-dihydroxy-3′,7-dimethoxyflavanone to yield naringenin (4′,5,7-trihydroxyflavanone) and homoeriodictyol (4′,5,7-trihydroxy-3′-methoxyflavanone). On the basis of their review, Cao et al. suggested that the demethylation of polymethoxy flavonoid derivatives occurs more frequently for the C-3′and C-4′ positions [25]. Our experience shows that strains of the genus Isaria perform demethylation only in the case of methoxy substituents in ring B. To time we performed biotransformations of over thirty variously substituted flavones and flavanones using entomopathogenic filamentous fungi as biocatalysts. Only for flavanones with methoxyl groups in ring B did we observe the glycosylation into the C-6 position for flavonoid derivatives lacking the hydroxy group in this position. In the case of 6-methoxyflavanone and 6-methoxyflavone, 4-*O*-methylglucopyranose was attached most frequently to C-4′, but also to C-3′ for 6-methoxyflavone. When we used the above-mentioned 6-methylflavone as a substrate, we also observed the formation of 6-methylflavone 8-*O*-β-d-(4″-*O*-methyl)-glucopyranoside [20].

A reaction, which we did not observe previously for other flavonoid substrates biotransformed in the cultures of entomopathogenic filamentous fungi was the reduction of carbonyl group at C-4. It occurred in the case of 2′-methoxyflavanone and 3′-methoxyflavanone.

## 3. Materials and Methods

### 3.1. Chemicals

The compound 6-methoxyflavanone was purchased from the Sigma Chemical Company (St. Louis, MO, USA). The compound 6-methoxyflavanone was purchased from TCI EUROPE N.V. (Zwijndrecht, Belgium). The compounds 2′-methoxyflavanone, 3′-methoxyflavanone, and 4′-methoxyflavanone were chemically synthesized.

#### 3.1.1. 2′-Methoxyflavanone (**1**)

C_16_H_14_O_3_, *t*_R_ 20.85; ^1^H-NMR, see Table 1; ^13^C-NMR, see Table 2.

#### 3.1.2. 3′-Methoxyflavanone (**2**)

C_16_H_14_O_3_, *t*_R_ 19.30; ^1^H-NMR, see Table 1; ^13^C-NMR, see Table 2.

#### 3.1.3. 4′-Methoxyflavanone (**3**)

C_16_H_14_O_3_, *t*_R_ 19.01; ^1^H-NMR, see Table 3; ^13^C-NMR, see Table 4.

#### 3.1.4. 6-Methoxyflavanone (**4**)

C_16_H_14_O_3_, *t*_R_ 21.36; ^1^H-NMR, see Table 5; ^13^C-NMR, see Table 6.

#### 3.1.5. 6-Methoxyflavone (**5**)

C_16_H_12_O_3_, *t*_R_ 19.63; ^1^H-NMR, see Table 5; ^13^C-NMR, see Table 6.

#### 3.1.6. Chemical Synthesis of 2′-Methoxyflavanone (**1**), 3′-Methoxyflavanone (**2**), 4′-Methoxyflavanone (**3**)

The compounds 2′-methoxyflavanone (**1**), 3′-methoxyflavanone (**2**), and 4′-methoxyflavanone (**3**) were prepared according to the method we described earlier [26]. A total of 10 g of the 2′-hydroxychalcone with a metoxy substituent (obtained from 2′-hydroxyacetophenone and methoxy-substituted benzaldehyde) was dissolved in ethanol (75 mL) with the addition of sodium acetate (8 g), and the reaction mixture was refluxed for 48 h. Crystallization from ethanol afforded pure methoxyflavanone.

### 3.2. Microorganism

The molecular identification of the strain *I. fumosorosea* KCH J2 was described in our previous publication [20]. The microorganism was stored at 4 °C on potato slants and freshly subcultured before use.

### 3.3. Analysis

The course of the biotransformation was determined with the use of chromatographic methods (TLC, HPLC). The TLC analysis was carried out on TLC Silica gel 60/Kieselguhr F254 plates (Merck, Darmstadt, Germany) with a mixture of chloroform and methanol in the ratio of 9:1 as developing system. The solution of 5% aluminum chloride in ethanol was used as the staining solution. The plates were observed at two wavelengths: 254 and 365 nm.

HPLC analyses were performed with a Waters 2690 instrument equipped with a Waters 996 photodiode array detector, using an ODS 2 column (4.6 × 250 mm, Waters, Milford, MA, USA) and a Guard-Pak Inserts µBondapak C18 pre-column. The following separation conditions were used: gradient elution, using 80% of acetonitrile in 4.5% acetic acid solution (eluent A) and 4.5% acetic acid (eluent B); flow: 1 mL/min; detection wavelength 280 nm; program: 0–7 min, 10% A 90% B; 7–10 min, 50% A 50% B; 10–13 min, 60% A 40% B; 13–15 min, 70% A 30% B; 15–20 min 80% A 20% B; 20–30 min 90% A 10% B; 30–40 min, 100% A.

Enantiomeric excess values were determined using a Chiralpak AD-H HPLC column, 4.6 mm × 250 mm (Diacel), with hexane:isopropanol (9:1) as the eluent (isocratic resolution).

Separation of the products obtained by the scale-up biotransformation of 3′-methoxyflavanone, 4′-methoxyflavanone, 6-methoxyflavanone, and 6-methoxyflavone was accomplished using a 1000 μm preparative TLC silica gel plates (Analtech, Gehrden, Germany). The developing system was a mixture of chloroform and methanol (9:1). After the separation, selected fractions were scraped out and compounds were extracted using ethyl acetate (twice) and tetrahydrofuran (once). The extracts were combined and the solvents were removed using a rotary evaporator.

Products obtained by the scale-up biotransformation of 2′-methoxyflavanone were separated with the use of Reveleris^®^ PREP Purification System (BUCHI Corporation, Flawil, Switzerland) using Reveleris^®^ Silica 4 g column. The separation conditions were as follows: gradient elution, using chloroform (eluent A) and methanol (eluent B); flow: 20 mL/min; detection wavelengths: 254 nm, 290 nm, and 360 nm; mode: Flash Dry.

NMR spectra were obtained using a Bruker Avance600 MHz NMR spectrometer (Bruker, Billerica, MA, USA) with an UltraShield Plusmagnet.

Molecular formulas of products were confirmed with the use of high-pressure liquid chromatography HPLC 1200 Agilent Technologies with DAD, FLD, and Mass detectors Triple Quad LC/MS (Agilent Technologies, Santa Clara, CA, USA).

Optical rotation was measured by means of the digital polaimeter P-2000-Na (ABL&E-JASCO, Kraków, Poland).

### 3.4. Screening Procedure

Sabouraud medium (1% peptone, 3% glucose) was used in all the experiments. The fungus was transferred to a 300 mL Erlenmeyer flask containing 100 mL of the medium. Pre-incubation was conducted at 25 °C for 72 h using a rotary shaker (140 rpm). The screening was performed in 100 mL Erlenmeyer flasks containing 30 mL of the medium. The medium was inoculated wit 0.5 mL of pre-grown culture and after 72-h incubation, 3 mg of the substrate dissolved in 0.5 mL of tetrahydrofuran was added. A separate flask of microbial culture was used for each sample collection. The biotransformation was carried out under the same conditions as pre-incubation. After 2, 5, and 12 days of biotransformation, the metabolites were extracted adding 30 mL of ethyl acetate to the fungal culture. The mixture was then shaken and the organic phase (extract) was separated. The extracts were dried over MgSO_4_ (5 min), concentrated on a rotary evaporator and analyzed using TLC and HPLC.

The stability of the substrate was determined under the same conditions, without the microorganism.

### 3.5. Scale-Up Biotransformations

Scale-up biotransformations were conducted in 2 L flasks containing 500 mL of the Sabouraud medium. A duration of 72 h of incubation followed the inoculation after which 50 mg of the substrate dissolved in 1 mL of tetrahydrofuran was added. The scale-up biotransformation was conducted under analogous conditions to the screening (140 rpm, 25 °C). After the time was determined on the base of the screening experiments (different for each substrate used), the metabolites were extracted using 200 mL of ethyl acetate (repeated three times, the procedure was as in the case of screening). The extracts were dried over MgSO_4_ and concentrated in vacuo. Product separation was carried out using preparative TLC plates or flash chromatography (in the case of 2′-methoxyflavanone (**1**)). The products structures were confirmed by means of spectroscopic methods (^1^H-NMR, ^13^C-NMR, COSY, HMBC, HSQC).

The physical and spectral data of the products obtained are presented below (Table 1, Table 2, Table 3, Table 4, Table 5 and Table 6) (Supplementary Materials).

#### 3.5.1. 2′-Methoxyflavanone 5′-*O*-β-d-(4″-*O*-methyl)-glucopyranoside (**1a**)

C_23_H_26_O_9_, M_w_ = 446.4, *t*_R_ 11.87, ${\left[\alpha \right]}_{D}^{20}=$ −5.4°, ^1^H-NMR, see Table 1; ^13^C-NMR, see Table 2.

#### 3.5.2. Flavan-4-ol 2′-*O*-β-d-(4″-*O*-methyl)-glucopyranoside (**1b**)

C_22_H_26_O_8_, M_w_ = 418.4, *t*_R_ 12.16, ${\left[\alpha \right]}_{D}^{20}=$ −25.7°, ^1^H-NMR, see Table 1; ^13^C-NMR, see Table 2.

#### 3.5.3. Flavan-4-ol 3′-*O*-β-d-(4″-*O*-methyl)-glucopyranoside (**2a**)

C_22_H_26_O_8_, M_w_ = 418.4, *t*_R_ 10.90, ${\left[\alpha \right]}_{D}^{20}=$ −6.5°, ^1^H-NMR, see Table 1; ^13^C-NMR, see Table 2.

#### 3.5.4. 3′-Hydroxyflavanone 6-*O*-β-d-(4″-*O*-methyl)-glucopyranoside (**2b**)

C_22_H_24_O_9_, M_w_ = 432.4, *t*_R_ 10.54, ${\left[\alpha \right]}_{D}^{20}=$ −24.0°, ^1^H-NMR, see Table 1; ^13^C-NMR, see Table 2.

#### 3.5.5. Flavanone 4′-*O*-β-d-(4″-*O*-methyl)-glucopyranoside (**3a**)

C_22_H_24_O_8_, M_w_ = 416.4, *t*_R_ 11.49, ${\left[\alpha \right]}_{D}^{20}=$ −19.1°, ^1^H-NMR, see Table 3; ^13^C-NMR, see Table 4.

#### 3.5.6. 4′-Hydroxyflavanone 6-*O*-β-d-(4″-*O*-methyl)-glucopyranoside (**3b**)

C_22_H_24_O_9_, M_w_ = 432.4, *t*_R_ 10.34, ${\left[\alpha \right]}_{D}^{20}=$ −36.0° ^1^H-NMR, see Table 3; ^13^C-NMR, see Table 4.

#### 3.5.7. 3′,4′-Dihydroxyflavanone 6-*O*-β-d-(4″-*O*-methyl)-glucopyranoside (**3c**)

C_22_H_24_O_10_, M_w_ = 448.4, *t*_R_ 10.01, ${\left[\alpha \right]}_{D}^{20}=$ −25.7°, ^1^H-NMR, see Table 3; ^13^C-NMR, see Table 4.

#### 3.5.8. 6-Methoxyflavanone 4′-*O*-β-d-(4″-*O*-methyl)-glucopyranoside (**4a**)

C_23_H_26_O_9_, M_w_ = 446.4, *t*_R_ 11.68, ${\left[\alpha \right]}_{D}^{20}=$ −19.2°, ^1^H-NMR, see Table 5; ^13^C-NMR, see Table 6.

#### 3.5.9. 3′-Hydroxy-6-methoxyflavanone 4′-*O*-β-d-(4″-*O*-methyl)-glucopyranoside (**4b**)

C_23_H_26_O_10_, M_w_ = 462.4, *t*_R_ 10.45, ${\left[\alpha \right]}_{D}^{20}=$ −19.9°, ^1^H-NMR, see Table 5; ^13^C-NMR, see Table 6.

#### 3.5.10. 6-Methoxyflavone 3′-*O*-β-d-(4″-*O*-methyl)-glucopyranoside (**5a**)

C_23_H_24_O_9_, M_w_ = 444.4, *t*_R_ 11.23, ${\left[\alpha \right]}_{D}^{20}=$ −34.2°, ^1^H-NMR, see Table 5; ^13^C-NMR, see Table 6.

#### 3.5.11. 6-Methoxyflavone 4′-*O*-β-d-(4″-*O*-methyl)-glucopyranoside (**5b**)

C_23_H_24_O_9_, M_w_ = 444.4, *t*_R_ 10.95, ${\left[\alpha \right]}_{D}^{20}=$ −13.0°, ^1^H-NMR, see Table 5; ^13^C-NMR, see Table 6.

#### 3.5.12. 3′-Hydroxy-6-methoxyflavone 4′-*O*-β-d-(4″-*O*-methyl)-glucopyranoside (**5c**)

C_23_H_24_O_10_, M_w_ = 460.4, *t*_R_ 10.81, ${\left[\alpha \right]}_{D}^{20}=$ −25.1°, ^1^H-NMR, see Table 5; ^13^C-NMR, see Table 6.

## 4. Conclusions

Hereby, we reported on microbial glycosylation of methoxylated flavonoids. The strain *I. fumosorosea* KCH J2 has an unprecedented ability to attach a sugar unit to flavonoid aglycones lacking hydroxyl groups. In the case of flavanones with methoxy substituents in ring B, 4-*O*-methylglucopyranose was attached to ring B carbons but also attached to the C-6 position. In the case of 6-methoxyflavanone and 6-methoxyflavone glycosylation occurred only in the B ring. As a result, we obtained twelve flavonoid 4-*O*-methylglucopyranosides. None of the products has been previously reported in the literature. The method presented by us is relatively easy, cheap, and environmentally friendly and it allows us to obtain products in amounts that enable their further use, for example, to assess their biological activities or to evaluate the bioavailability of flavonoid sugar derivatives.

## Figures and Tables

**Table 1:** The ^1^H-NMR shifts (δ) (ppm) and coupling constants (*J*_H,H_) (Hz) of 2′-methoxyflavanone (**1**) and 3′-methoxyflavanone (**2**) and the products of their biotransformations (**1a**, **1b**, **2a**, **2b**) in Acetone-d_6_, 600 MHz (Supplementary Materials).

Proton	Compound					
	1	1a	1b	2	2a	2b
**H-2**	5.89 (dd) *J*_2,3a_ = 13.3, *J*_2,3e_ = 2.8	5.85 (dd) *J*_2,3a_ = 13.0, *J*_2,3e_ = 2.8	5.84 (d) *J* = 11.4	5.65 (dd) *J*_2,3a_ = 13.0, *J*_2,3e_ = 2.9	5.31 (dd) *J*_2,3a_ = 11.7, *J*_2,3e_ = 2.1	5.57 (dd) *J*_2,3a_ = 12.7, *J*_2,3e_ = 3.0
**H-3_a_**	3.05 (dd) *J*_3a,2_ = 13.3, *J*_3a,3e_ = 16.7	3.01 (m)	2.49 (d) *J* = 14.0	3.19 (dd) *J*_3a,2_ = 13.1, *J*_3a,3e_ = 16.8	2.26 (m)	3.12 (dd) *J*_3a,2_ = 12.7, *J*_3a,3e_ = 16.8
**H-3_e_**	2.88 (m)	2.88 (m)	2.01 (m)	2.90 (dd) *J*_3e,2_ = 2.9, *J*_3e,3a_ = 16.8	2.15 (m)	2.87 (m)
**H-4**	-	-	4.83 (m)	-	4.81 (m)	-
**H-5**	7.90 (dd) *J*_5,6_ = 8.2, *J*_5,7_ = 1.8	7.89 (dd) *J*_5,6_ = 7.8, *J*_5,7_ = 1.6	7.60 (d) *J*_5,6_ = 7.0	7.88 (dd) *J*_5,6_ = 8.0, *J*_5,7_ = 1.8	7.39 (dd) *J*_5,6_ = 7.6, *J*_5,7_ = 1.6	7.49 (t) *J* = 3.1
**H-6**	7.12 (m)	7.13 (m)	7.19 (t) *J* = 7.4	7.13 (m)	6.96(dt) *J* = 7.4, *J* = 1.1	-
**H-7**	7.62 (m)	7.63 (m)	7.33 (m)	7.63 (ddd) *J*_7,5_ = 1.7, *J*_7,6_ = 7.2, *J*_7,8_ = 8.3	7.25 (m)	7.37 (dd) *J*_7,5_ = 3.1, *J*_7,8_ = 9.0
**H-8**	7.12 (m)	7.13 (m)	7.33 (m)	7.13 (m)	6.91 (dd) *J*_8,6_ = 0.9, *J*_8,7_ = 8.1	7.07 (m)
**H-2′**				7.22 (m)	7.23 (m)	7.07 (m)
**H-3′**	7.12 (m)	7.03 (d) *J*_3′,4′_ = 8.9	6.92 (d) *J*_3′,4′_ = 8.2	-	-	-
**H-4′**	7.41 (m)	7.11 (dd) *J*_4′,3′_ = 9.1, *J*_4′,6′_ = 2.9	4.25 (m)	7.00 (ddd) *J*_4′,2′_ = 0.8, *J*_4′,5′_ = 8.3, *J*_4′,6′_ = 2.6	7.08 (ddd) *J*_4′,2′_ = 0.7, *J*_4′,5′_ = 8.5, *J*_4′,6′_ = 2.6	6.89 (ddd) *J*_4′,2′_ = 0.8, *J*_4′,5′_ = 8.1, *J*_4′,6′_ = 2.3
**H-5′**	7.12 (m)	-	6.96 (t) *J* = 7.4	7.40 (m)	7.36 (t) *J* = 8.0	7.29 (t) *J* = 7.9
**H-6′**	7.68 (dd) *J*_6′,4′_ = 1.5, *J*_6′,5′_ = 7.6	7.41 (d) *J*_6′,4′_ = 2.8	7.42 (d) *J* = 6.5	7.19 (m)	7.17 (d) *J*_6′,5′_ = 7.6	7.07 (m)
**H-1″**	-	4.90 (d) *J* = 7.8	4.85 (d) *J* = 7.8	-	5.03 (d) *J* = 7.8	4.93 (d) *J* = 7.8
**H-2″**	-	3.52 (m)	3.53 (m)	-	3.51 (m)	3.49 (m)
**H-3″**	-	3.65 (m)	3.63 (t) *J* = 8.5	-	3.66 (m)	3.67 (dt) *J* = 9.0, *J* = 3.7
**H-4″**	-	3.23 (m)	3.28 (t) *J* = 9.3	-	3.26 (m)	3.27 (t) *J* = 9.3
**H-5″**	-	3.48 (m)	3.42 (ddd) *J* = 9.4, *J* = 4.0, *J* = 2.0	-	3.51 (m)	3.49 (m)
**H-6″**	-	3.72 (m) 3.85 (m)	3.84 (dd) *J* = 5.2, *J* = 2.4 3.74 (dd) *J* = 11.0, *J* = 3.8	-	3.85 (m) 3.72 (m)	3.87 (m) 3.76 (m)
**C-4″-OCH_3_**	-	3.59 (s)	3.59 (s)	-	3.60 (s)	3.60 (s)
**C-2′-OCH_3_**	3.92 (s)	3.89 (s)	-	-	-	-
**C-3′-OCH_3_**	-	-	-	3.88 (s)	-	-
**C-3′-OH**	-	-	-	-	-	8.54 (s)

**Table 2:** The ^13^C-NMR shifts (δ) (ppm) of 2′-methoxyflavanone (**1**) and 3′-methoxyflavanone (**2**) and the products of their biotransformations (**1a**, **1b**, **2a**, **2b**) in Acetone-d_6_, 151 MHz (Supplementary Materials).

Carbon	Compound					
	1	1a	1b	2	2a	2b
**C-2**	75.5	75.0	68.9	80.3	73.8	80.3
**C-3**	44.1	44.0	38.4	45.0	39.6	44.9
**C-4**	192.1	192.1	64.3	191.8	63.6	191.7
**C-4a**	121.9	121.9	133.2	122.0	126.1	122.1
**C-5**	127.4	127.4	127.2	127.4	131.5	113.7
**C-6**	122.2	122.3	124.1	122.3	121.1	153.1
**C-7**	136.8	136.8	129.5	136.8	129.9	126.8
**C-8**	118.9	119.0	118.5	118.9	117.5	118.2
**C-8a**	162.8	162.7	156.1	162.4	155.6	157.8
**C-1′**	128.4	129.2	125.7	141.9	144.2	141.9
**C-2′**	157.0	152.0	155.4	112.9	115.4	114.2
**C-3′**	111.8	112.7	117.6	160.9	158.9	158.5
**C-4′**	130.4	117.8	130.0	114.7	116.5	116.2
**C-5′**	121.6	152.8	121.1	130.6	130.3	130.6
**C-6′**	127.4	116.3	131.7	119.3	120.7	119.9
**C-1″**	-	102.4	104.3	-	101.5	102.6
**C-2″**	-	75.3	75.1	-	75.0	75.0
**C-3″**	-	78.0	77.6	-	78.0	77.8
**C-4″**	-	80.2	79.8	-	80.1	79.9
**C-5″**	-	77.0	76.9	-	77.0	77.0
**C-6″**	-	62.2	61.8	-	62.1	62.0
**C-4″-OCH_3_**	-	60.5	60.5	-	60.5	60.5
**C-2′-OCH_3_**	56.0	56.4	-	-	-	-
**C-3′-OCH_3_**	-	-	-	55.6	-	-

**Table 3:** The ^1^H-NMR shifts (δ) (ppm) and coupling constants (*J*_H,H_) (Hz) of 4′-methoxyflavanone (**3**) and the products of its biotransformations (**3a**, **3b**, **3c**) in Acetone-d_6_, 600 MHz (Supplementary Materials).

Proton	Compound			
	3	3a	3b	3c
**H-2**	5.60 (dd) *J*_2,3a_ = 13.1, *J*_2,3e_ = 2.8	5.63 (dd) *J*_2,3a_ = 13.0, *J*_2,3e_ = 2.8	5.51 (ddd) *J*_2,3a_ = 13.1, *J*_2,3e_ = 2.8 *J* = 4.8	5.45 (ddd) *J*_2,3a_ = 12.9, *J*_2,3e_ = 2.9 *J* = 5.2
**H-3_a_**	3.22 (dd) *J*_3a,2_ = 13.1, *J*_3a,3e_ = 16.7	3.21 (m)	3.17 (ddd) *J*_3a,2_ = 13.2, *J*_3a,3e_ = 16.8, *J* = 0.9	3.13 (ddd) *J*_3a,2_ = 12.9, *J*_3a,3e_ = 16.8, *J* = 1.3
**H-3_e_**	2.85 (dd) *J*_3e,2_ = 2.9, *J*_3e,3a_ = 16.8	2.86 (m)	2.81 (dt) *J* = 16.8, *J* = 3.0	2.81 (m)
**H-5**	7.88 (dd) *J*_5,6_ = 7.7, *J*_5,7_ = 1.8	7.88 (dd) *J*_5,6_ = 7.8, *J*_5,7_ = 1.7	5.49 (t) *J* = 3.0	7.49 (t) *J* = 3.0
**H-6**	7.12 (m)	7.11 (m)	-	-
**H-7**	7.61 (m)	7.61 (m)	7.35 (dd) *J*_7,5_ = 3.1, *J*_7,8_ = 9.0	7.35 (dd) *J*_7,5_ = 3.1, *J*_7,8_ = 9.0
**H-8**	7.09 (d) *J*_8,7_ = 8.7	7.11 (m)	7.03 (dd) *J*_8,7_ = 8.9, *J* = 1.4	7.02 (dd) *J*_8,7_ = 9.0, *J* = 1.4
**H-2′**	7.56 (m)	7.55 (m)	6.44 (m)	7.08 (t) *J* = 1.9
**H-3′**	7.04 (m)	7.16 (m)	6.93 (m)	-
**H-5′**	7.04 (m)	7.16 (m)	6.93 (m)	6.91 (m)
**H-6′**	7.56 (m)	7.55 (m)	6.44 (m)	6.91 (m)
**H-1″**	-	5.02 (dd) *J* = 7.8, *J* = 1.9	4.93 (d) *J* = 7.7	4.92 (d) *J* = 6.3
**H-2″**	-	3.52 (m)	3.49 (m)	3.49 (m)
**H-3″**	-	3.67 (dt) *J* = 9.1, *J* = 3.9	3.67 (m)	3.67 (t) *J* = 9.0
**H-4″**	-	3.25 (m)	3.27 (t) *J* = 9.3	3.27 (t) *J* = 9.3
**H-5″**	-	3.52 (m)	3.49 (m)	3.49 (m)
**H-6″**	-	3.87 (m) 3.74 (m)	3.87 (m) 3.75 (m)	3.87 (d) *J* = 11.4 3.75 (dd) *J* = 10.9, *J* = 5.5
**C-4″-OCH_3_**	-	3.60 (s)	3.60 (s)	3.60 (s)
**C-4′-OCH_3_**	3.87 (s)	-	-	-
**C-4′-OH**	-	-	8.57 (s)	-

**Table 4:** The ^13^C-NMR shifts (δ) (ppm) of 4′-methoxyflavanone (**3**) and the products of its biotransformations (**3a**, **3b**, **3c**) in Acetone-d_6_, 600 MHz (Supplementary Materials).

Carbon	Compound			
	3	3a	3b	3c
**C-2**	80.2	80.1	80.4	80.4
**C-3**	44.8	44.8	44.8	44.8
**C-4**	192.1	192.0	192.1	192.1
**C-4a**	121.9	121.9	122.0	122.0
**C-5**	127.4	127.4	113.7	113.7
**C-6**	122.1	122.2	153.0	153.0
**C-7**	136.8	136.8	126.8	126.8
**C-8**	118.9	118.9	119.9	119.9
**C-8a**	162.5	162.5	158.0	157.9
**C-1′**	132.2	133.8	131.1	131.9
**C-2′**	128.9	128.7	129.0	114.7
**C-3′**	114.8	117.4	116.2	146.0
**C-4′**	160.9	158.9	158.6	146.3
**C-5′**	114.8	117.4	116.2	119.2
**C-6′**	128.9	128.7	129.0	116.0
**C-1″**	-	101.6	102.7	102.6
**C-2″**	-	74.0	75.0	75.0
**C-3″**	-	78.0	77.9	77.8
**C-4″**	-	80.1	79.9	79.9
**C-5″**	-	77.1	77.0	76.9
**C-6″**	-	62.1	62.0	62.0
**C-4″-OCH_3_**	-	60.6	60.5	60.5
**C-4′-OCH_3_**	55.6	-	-	-

**Table 5:** The ^1^H-NMR shifts (δ) (ppm) and coupling constants (*J*_H,H_) (Hz) of 6-methoxyflavanone (**4**) and 6-methoxyflavone (**5**) and the products of their biotransformations (**4a**, **4b**, **5a**, **5b**, **5c**) in Acetone-d_6_, 600 MHz (Supplementary Materials).

Proton	Compound						
	4	4a	4b	5	5a	5b	5c
**H-2**	5.62 (dd) *J*_2,3a_ = 13.1, *J*_2,3e_ = 2.9	7.56 (dd) *J*_2,3a_ = 13.0, *J*_2,3e_ = 2.8	5.52 (dd) *J*_2,3a_ = 12.8, *J*_2,3e_ = 2.9	-	-	-	-
**H-3_a_**	3.15 (dd) *J*_3a,2_ = 13.1, *J*_3a,3e_ = 16.8	3.17 (dd) *J*_3a,2_ = 13.0, *J*_3a,3e_ = 16.8	3.14 (m)	-	-	-	-
**H-3_e_**	2.88 (m)	2.87 (m)	2.85 (m)				
**H-3 (flavone)**	-	-	-	6.88 (s)	6.88 (s)	6.80 (s)	6.79 (s)
**H-5**	7.33 (d) *J*_5,7_ = 3.2	7.32 (d) *J*_5,7_ = 3.1	7.31 (m)	7.54 (d) *J*_5,7_ = 3.1	7.54 (m)	7.54 (d) *J*_5,7_ = 3.1	7.54 (d) *J*_5,7_ = 3.1
**H-7**	7.23 (dd) *J*_7,5_ = 3.2, *J*_7,8_ = 9.0	7.22 (dd) *J*_7,5_ = 3.2, *J*_7,8_ = 9.0	7.21 (m)	7.43 (dd) *J*_7,5_ = 3.1, *J*_7,8_ = 9.1	7.43 (dd) *J*_7,5_ = 3.2, *J*_7,8_ = 9.1	7.41 (dd) *J*_7,5_ = 3.2, *J*_7,8_ = 9.1	7.42 (dd) *J*_7,5_ = 3.2, *J*_7,8_ = 9.1
**H-8**	7.08 (d) *J*_8,7_ = 9.0	7.05 (d) *J*_8,7_ = 9.0	7.05 (m)	7.73 (d) *J*_8,7_ = 9.1	7.78 (d) *J*_8,7_ = 9.1	7.72 (d) *J*_8,7_ = 9.1	7.74 (d) *J*_8,7_ = 9.1
**H-2′**	7.62 (d) *J*_2′,3′_ = 7.4	7.54 (d) *J*_2′,3′_ = 8.6	7.14 (d) *J*_2′,5′_ = 2.2	8.13 (m)	7.81 (m)	8.08 (m)	7.61 (m)
**H-3′**	7.49 (t) *J* = 7.5	7.15 (d) *J*_3′,2′_ = 8.6	-	7.64 (m)	-	7.28 (m)	-
**H-4′**	7.43 (m)	-	-	7.64 (m)	7.31 (m)	-	-
**H-5′**	7.49 (t) *J* = 7.5	7.15 (d) *J*_5′,6′_ = 8.6	7.00 (dd) *J*_5′,2′_ = 2.1, *J*_5′,6′_ = 8.4	7.64 (m)	7.54 (m)	7.28 (m)	7.37 (d) *J*_5′,6′_ = 8.5
**H-6′**	7.62 (d) *J*_6′,5′_ = 7.4	7.54 (d) *J*_6′,5′_ = 8.6	7.24 (d) *J*_6′,5′_ = 8.3	8.13 (m)	7.77 (m)	8.08 (m)	7.58 (dd) *J*_6′,2′_ = 2.3, *J*_6′,5′_ = 8.5
**H-1″**	-	5.01 (d) *J* = 7.8	4.81 (d) *J* = 7.9	-	5.14 (d) *J* = 7.8	5.14 (d) *J* = 7.8	5.00 (d) *J* = 7.8
**H-2″**	-	3.51 (m)	3.53 (m)	-	3.57 (m)	3.55 (m)	3.57 (m)
**H-3″**	-	3.67 (t) *J* = 8.9	3.67 (t) *J* = 9.0	-	3.71 (m)	3.69 (m)	3.72 (m)
**H-4″**	-	3.26 (t) *J* = 9.3	3.26 (m)	-	3.25 (dd) *J* = 9.5, *J* = 9.1	3.28 (m)	3.29 (m)
**H-5″**	-	3.53 (m)	3.50 (m)	-	3.65 (ddd) *J* = 9.7, *J* = 5.5, *J* = 1.9	3.59 (m)	3.57 (m)
**H-6″**	-	3.86 (m) 3.72 (dd) *J* = 12.0, *J* = 4.8	3.88 (m) 3.73 (m)	-	3.94 (m) 3.76 (m)	3.90 (m) 3.75 (m)	3.92 (m) 3.77 (m)
**C-4″-OCH_3_**	-	3.60 (s)	3.60 (s)	-	3.62 (s)	3.61 (s)	3.61 (s)
**C-6-OCH_3_**	3.86 (s)	3.85 (s)	3.85 (s)	3.96 (s)	3.97 (s)	3.96 (s)	3.96 (s)

**Table 6:** The ^13^C-NMR shifts (δ) (ppm) of 6-methoxyflavanone (**4**) and 6-methoxyflavone (**5**) and the products of their biotransformations (**4a**, **4b**, **5a**, **5b**, **5c**) in Acetone-d_6_, 151 MHz (Supplementary Materials).

Carbon	Compound						
	4	4a	4b	5	5a	5b	5c
**C-2**	80.4	80.1	80.1	163.6	163.2	163.5	163.4
**C-3**	45.0	44.8	44.9	107.2	107.4	106.1	106.4
**C-4**	191.8	192.0	191.9	177.7	177.7	177.6	177.6
**C-4a**	121.9	121.9	121.9	125.5	125.4	125.5	125.5
**C-5**	108.3	108.3	108.3	105.8	105.7	105.8	105.8
**C-6**	155.2	155.1	155.1	158.0	158.1	158.0	158.0
**C-7**	125.3	125.2	125.2	124.0	124.0	123.8	123.8
**C-8**	120.2	120.2	120.2	120.8	120.8	120.7	120.7
**C-8a**	156.9	157.0	156.9	151.8	151.8	151.7	151.7
**C-1′**	140.5	133.9	136.3	132.9	134.2	126.3	128.0
**C-2′**	127.3	128.7	115.0	127.1	115.0	128.7	114.5
**C-3′**	129.5	117.4	148.8	130.0	159.3	117.6	148.8
**C-4′**	129.3	158.9	146.2	132.4	120.6	161.3	148.9
**C-5′**	129.5	117.4	118.6	130.0	131.0	117.6	118.3
**C-6′**	127.3	128.7	119.4	127.1	120.9	128.7	119.2
**C-1″**	-	101.6	104.3	-	101.7	101.2	103.2
**C-2″**	-	74.8	74.8	-	75.0	74.9	74.9
**C-3″**	-	77.9	78.7	-	78.1	78.0	77.4
**C-4″**	-	80.1	80.0	-	80.3	80.1	80.0
**C-5″**	-	77.1	77.3	-	77.3	77.2	77.4
**C-6″**	-	62.0	61.9	-	62.2	62.1	62.0
**C-4″-OCH_3_**	-	60.6	60.6	-	60.6	60.6	60.6
**C-6-OCH_3_**	56.0	56.1	56.0	56.2	56.2	56.2	56.2

## Supplementary Materials

Supplementary Materials can be found online.

## References

[1] Cassidy A. (2018). Berry anthocyanin intake and cardiovascular health. Mol. Aspects Med..

[2] Lin M., Zhang J., Chen X. (2018). Bioactive flavonoids in *Moringa oleifera* and their health-promoting properties. J. Funct. Foods.

[3] Deng J., Yang H., Capanoglu E., Cao H., Xiao J. (2018). Technological Aspects and Stability of Polyphenols.

[4] Wang W., Sun C., Mao L., Ma P., Liu F., Yang J., Gao Y. (2016). The biological activities, chemical stability, metabolism and delivery systems of quercetin: A review. Trends Food Sci. Technol..

[5] Aklaghi M., Ghobadi S., Mohammad Hosseini M., Gholami Z., Mohammadian F. (2018). Flavanols are potential anti-obesity agents, a systematic review and meta-analysis of controlled clinical trials. Nutr. Metab. Cardiovasc. Dis..

[6] Belščak-Cvitanović A., Durgo K., Huđek A., Bačun-Družina V., Komes D., Galanakis C. (2018). Overview of Polyphenols and Their Properties.

[7] Perez-Vizcaino F., Fraga C.G. (2018). Research trends in flavonoids and health. Arch. Biochem. Biophys..

[8] Wang T.-Y., Li Q., Bi K.-S. (2018). Bioactive flavonoids in medicinal plants: Structure, activity and biological fate. Asian J. Pharm. Sci..

[9] Kumar S., Pandey A.K. (2013). Chemistry and Biological Activities of Flavonoids: An Overview. Sci. World J..

[10] Treml J., Smejkal K. (2016). Flavonoids as Potent Scavengers of Hydroxyl Radicals. Compr. Rev. Food Sci. Food Saf..

[11] Symonowicz M., Kolanek M. (2012). Flavonoids and their properties to form chelate complexes. Biotechnol. Food Sci..

[12] Gao Z., Gao W., Zeng S., Li P., Liu E. (2018). Chemical structures, bioactivities and molecular mechanisms of citrus polymethoxy flavones. J. Funct. Foods.

[13] Hung W., Chang W., Lu W., Wei G., Wang Y., Ho C.-T., Hwang L.S. (2018). Pharmacokinetics, bioavailability, tissue distribution and excretion of tangeretin in rat. J. Food Drug Anal..

[14] Akbar S., Subhan F., Karim N., Shahid M., Ahmad N., Ali G., Mahmood W., Fawad K. (2016). 6-Methoxyflavanone attenuates mechanical allodynia and vulvodynia in the streptozotocin-induced diabetic neuropathic pain. Biomed. Pharmacother..

[15] Akbar S., Subhan F., Karim N., Aman U., Ullah S., Shahid M., Ahmad N., Fawad K., Sewell R.D.E. (2017). Characterization of 6-methoxyflavanone as a novel anxiolytic agent: A behavioral and pharmacokinetic approach. Eur. J. Pharmacol..

[16] Thilakarathna S.H., Vasantha Rupasinghe H.P. (2013). Flavonoid bioavailability and attempts for bioavailability enhancement. Nutrients.

[17] Yang B., Liu H., Yang J., Jiang Y. (2018). New insights on bioactivities and biosynthesis of flavonoid glycosides. Trends Food Sci. Technol..

[18] Mojzer E.B., Hrncic M.K., Škerget M., Knez Ž., Bren U. (2016). Polyphenols: Extraction methods, antioxidative action, bioavailability and anticarcinogenic effects. Molecules.

[19] Archivio M.D., Filesi C., Varì R., Scazzocchio B. (2010). Bioavailability of the polyphenols: Status and controversies. Int. J. Mol. Sci..

[20] Dymarska M., Grzeszczuk J., Urbaniak M., Janeczko T., Pląskowska E., Stępień Ł., Kostrzewa-Susłow E. (2017). Glycosylation of 6-methylflavone by the strain *Isaria fumosorosea* KCH J2. PLoS ONE.

[21] Dymarska M., Janeczko T., Kostrzewa-Susłow E. (2018). Biotransformations of flavones and an isoflavone (daidzein) in cultures of entomopathogenic filamentous fungi. Molecules.

[22] Dymarska M., Janeczko T., Kostrzewa-Susłow E. (2018). Glycosylation of 3-hydroxyflavone, 3-methoxyflavone, quercetin and baicalein in fungal cultures of the genus *Isaria*. Molecules.

[23] Kostrzewa-Susłow E., Dymarska M., Białońska A., Janeczko T. (2014). Enantioselective conversion of certain derivatives of 6-hydroxyflavanone. J. Mol. Catal. B Enzym..

[24] Kostrzewa-Susłow E., Dmochowska-Gładysz J., Janeczko T., Środa K., Michalak K., Palko A. (2012). Microbial transformations of 6- and 7-methoxyflavones in *Aspergillus niger* and *Penicillium chermesinum* cultures. Zeitschrift fur Naturforsch. Sect. C J. Biosci..

[25] Cao H., Chen X., Jassbi A.R., Xiao J. (2015). Microbial biotransformation of bioactive flavonoids. Biotechnol. Adv..

[26] Janeczko T., Dymarska M., Siepka M., Gniłka R., Leśniak A., Popłoński J., Kostrzewa-Susłow E. (2014). Enantioselective reduction of flavanone and oxidation of cis- and trans-flavan-4-ol by selected yeast cultures. J. Mol. Catal. B Enzym..

