# Supplementary material for: Glycosylation of Methoxylated Flavonoids in the Cultures of Isaria fumosorosea KCH J2
Source: Molecules. 2018 Oct 9;23(10):2578. doi: 10.3390/molecules23102578 (PMC6222689; doi:10.3390/molecules23102578)
Supplement: Supplementary file 1 [file molecules-23-02578-s001.pdf]

Supplementary data

Glycosylation of methoxylated flavonoids in the cultures of *Isaria fumosorosea* KCH J2

Monika Dymarska*, Tomasz Janeczko and Edyta Kostrzewa-Susłow

Department of Chemistry, Faculty of Biotechnology and Food Science, Wrocław University of Environmental and Life Sciences, Wrocław, Poland; janeczko13@interia.pl (T.J.); ekostrzew@gmail.com (E.K.-S.)

* Correspondence: monika.dymarska@gmail.com

Content

Figure S1. ^{1}H NMR spectrum of 2′-methoxyflavanone(1) (Acetone-d_6, 600 MHz)
Figure S2. ^{1}H NMR spectrum of 2′-methoxyflavanone(1) (Acetone-d_6, 600 MHz)
Figure S3. ^{13}C NMR spectrum of 2′-methoxyflavanone (1) (Acetone-d_6, 151 MHz)
Figure S4. HSQC NMR spectrum of 2′-methoxyflavanone (1) (Acetone-d_6, 151 MHz)
Figure S5. HSQC NMR spectrum of 2′-methoxyflavanone (1) (Acetone-d_6, 151 MHz)
Figure S6. HMBC NMR spectrum of 2′-methoxyflavanone (1) (Acetone-d_6, 151 MHz)
Figure S7. COSY NMR spectrum of 2′-methoxyflavanone (1) (Acetone-d_6, 600 MHz)
Figure S8. ^{1}H NMR spectrum of 2′-methoxyflavanone 5′-*O*-β-D-(4″-*O*-methyl)-glucopyranoside (1a) (Acetone-d_6, 600 MHz)
Figure S9. ^{1}H NMR spectrum of 2′-methoxyflavanone 5′-*O*-β-D-(4″-*O*-methyl)-glucopyranoside (1a) (Acetone-d_6, 600 MHz)
Figure S10. ^{13}C NMR spectrum of 2′-methoxyflavanone 5′-*O*-β-D-(4″-*O*-methyl)-glucopyranoside (1a) (Acetone-d_6, 151 MHz)
Figure S11. HSQC NMR spectrum of 2′-methoxyflavanone 5′-*O*-β-D-(4″-*O*-methyl)-glucopyranoside (1a) (Acetone-d_6, 151 MHz)
Figure S12. HSQC NMR spectrum of 2′-methoxyflavanone 5′-*O*-β-D-(4″-*O*-methyl)-glucopyranoside (1a) (Acetone-d_6, 151 MHz)
Figure S13. HSQC NMR spectrum of 2′-methoxyflavanone 5′-*O*-β-D-(4″-*O*-methyl)-glucopyranoside (1a) (Acetone-d_6, 151 MHz)
Figure S14. HMBC NMR spectrum 2′-methoxyflavanone 5′-*O*-β-D-(4″-*O*-methyl)-glucopyranoside (1a) (Acetone-d_6, 151 MHz)
Figure S15. COSY NMR spectrum of 2′-methoxyflavanone 5′-*O*-β-D-(4″-*O*-methyl)-glucopyranoside (1a) (Acetone-d_6, 600 MHz)

Figure S16. ^{1}H NMR spectrum of flavan-4-ol 2′-*O*-β-D-(4″-*O*-methyl)-glucopyranoside (1b) (Acetone-d_6, 600 MHz)
Figure S17. ^{1}H NMR spectrum of flavan-4-ol 2′-*O*-β-D-(4″-*O*-methyl)-glucopyranoside (1b) (Acetone-d_6, 600 MHz)
Figure S18. ^{13}C NMR spectrum of flavan-4-ol 2′-*O*-β-D-(4″-*O*-methyl)-glucopyranoside (1b) (Acetone-d_6, 151 MHz)
Figure S19. HSQC NMR spectrum of flavan-4-ol 2′-*O*-β-D-(4″-*O*-methyl)-glucopyranoside (1b) (Acetone-d_6, 151 MHz)
Figure S20. HSQC NMR spectrum of flavan-4-ol 2′-*O*-β-D-(4″-*O*-methyl)-glucopyranoside (1b) (Acetone-d_6, 151 MHz)
Figure S21. HSQC NMR spectrum of flavan-4-ol 2′-*O*-β-D-(4″-*O*-methyl)-glucopyranoside (1b) (Acetone-d_6, 151 MHz)
Figure S22. HSQC NMR spectrum of flavan-4-ol 2′-*O*-β-D-(4″-*O*-methyl)-glucopyranoside (1b) (Acetone-d_6, 151 MHz)
Figure S23. HMBC NMR spectrum of flavan-4-ol 2′-*O*-β-D-(4″-*O*-methyl)-glucopyranoside (1b) (Acetone-d_6, 151 MHz)
Figure S24. COSY NMR spectrum of flavan-4-ol 2′-*O*-β-D-(4″-*O*-methyl)-glucopyranoside (1b) (Acetone-d_6, 600 MHz)
Figure S25. ^{1}H NMR spectrum of 3′-methoxyflavanone (2) (Acetone-d_6, 600 MHz)
Figure S26. ^{1}H NMR spectrum of 3′-methoxyflavanone (2) (Acetone-d_6, 600 MHz)
Figure S27. ^{13}C NMR spectrum of 3′-methoxyflavanone (2) (Acetone-d_6, 151 MHz)
Figure S28. HSQC NMR spectrum of 3′-methoxyflavanone (2) (Acetone-d_6, 151 MHz)
Figure S29. HSQC NMR spectrum of 3′-methoxyflavanone (2) (Acetone-d_6, 151 MHz)
Figure S30. HMBC NMR spectrum of 3′-methoxyflavanone (2) (Acetone-d_6, 151 MHz)
Figure S31. COSY NMR spectrum of 3′-methoxyflavanone (2) (Acetone-d_6, 600 MHz)
Figure S32. ^{1}H NMR spectrum of flavan-4-ol 3′-*O*-β-D-(4″-*O*-methyl)-glucopyranoside (2a) (Acetone-d_6, 600 MHz)
Figure S33. ^{1}H NMR spectrum of flavan-4-ol 3′-*O*-β-D-(4″-*O*-methyl)-glucopyranoside (2a) (Acetone-d_6, 600 MHz)
Figure S34. ^{13}C NMR spectrum of flavan-4-ol 3′-*O*-β-D-(4″-*O*-methyl)-glucopyranoside (2a) (Acetone-d_6, 151 MHz)
Figure S35. HSQC NMR spectrum of flavan-4-ol 3′-*O*-β-D-(4″-*O*-methyl)-glucopyranoside (2a) (Acetone-d_6, 151 MHz)
Figure S36. HSQC NMR spectrum of flavan-4-ol 3′-*O*-β-D-(4″-*O*-methyl)-glucopyranoside (2a) (Acetone-d_6, 151 MHz)
Figure S37. HSQC NMR spectrum of flavan-4-ol 3′-*O*-β-D-(4″-*O*-methyl)-glucopyranoside (2a) (Acetone-d_6, 151 MHz)
Figure S38. HMBC NMR spectrum of flavan-4-ol 3′-*O*-β-D-(4″-*O*-methyl)-glucopyranoside (2a) (Acetone-d_6, 151 MHz)
Figure S39. COSY NMR spectrum of flavan-4-ol 3′-*O*-β-D-(4″-*O*-methyl)-glucopyranoside (2a) (Acetone-d_6, 600 MHz)

Figure S40. ^{1}H NMR spectrum of 3′-hydroxyflavanone 6-*O*-β-D-(4″-*O*-methyl)-glucopyranoside (2b) (Acetone-d_6, 600 MHz)
Figure S41. ^{1}H NMR spectrum of 3′-hydroxyflavanone 6-*O*-β-D-(4″-*O*-methyl)-glucopyranoside (2b) (Acetone-d_6, 600 MHz)
Figure S42. ^{13}C NMR spectrum of 3′-hydroxyflavanone 6-*O*-β-D-(4″-*O*-methyl)-glucopyranoside (2b) (Acetone-d_6, 151 MHz)
Figure S43. HSQC NMR spectrum of 3′-hydroxyflavanone 6-*O*-β-D-(4″-*O*-methyl)-glucopyranoside (2b) (Acetone-d_6, 151 MHz)
Figure S44. HSQC NMR spectrum of 3′-hydroxyflavanone 6-*O*-β-D-(4″-*O*-methyl)-glucopyranoside (2b) (Acetone-d_6, 151 MHz)
Figure S45. HSQC NMR spectrum of 3′-hydroxyflavanone 6-*O*-β-D-(4″-*O*-methyl)-glucopyranoside (2b) (Acetone-d_6, 151 MHz)
Figure S46. HMBC NMR spectrum of 3′-hydroxyflavanone 6-*O*-β-D-(4″-*O*-methyl)-glucopyranoside (2b) (Acetone-d_6, 151 MHz)
Figure S47. COSY NMR spectrum of 3′-hydroxyflavanone 6-*O*-β-D-(4″-*O*-methyl)-glucopyranoside (2b) (Acetone-d_6, 600 MHz)
Figure S48. ^{1}H NMR spectrum of 4′-methoxyflavanone (3) (Acetone-d_6, 600 MHz)
Figure S49. ^{1}H NMR spectrum of 4′-methoxyflavanone (3) (Acetone-d_6, 600 MHz)
Figure S50. ^{13}C NMR spectrum of 4′-methoxyflavanone (3) (Acetone-d_6, 151 MHz)
Figure S51. HSQC NMR spectrum of 4′-methoxyflavanone (3) (Acetone-d_6, 151 MHz)
Figure S52. HMBC NMR spectrum of 4′-methoxyflavanone (3) (Acetone-d_6, 151 MHz)
Figure S53. ^{1}H NMR spectrum of flavanone 4′-*O*-β-D-(4″-*O*-methyl)-glucopyranoside (3a) (Acetone-d_6, 600 MHz)
Figure S54. ^{1}H NMR spectrum of flavanone 4′-*O*-β-D-(4″-*O*-methyl)-glucopyranoside (3a) (Acetone-d_6, 600 MHz)
Figure S55. ^{13}C NMR spectrum of flavanone 4′-*O*-β-D-(4″-*O*-methyl)-glucopyranoside (3a) (Acetone-d_6, 151 MHz)
Figure S56. HSQC NMR spectrum of flavanone 4′-*O*-β-D-(4″-*O*-methyl)-glucopyranoside (3a) (Acetone-d_6, 151 MHz)
Figure S57. HSQC NMR spectrum of flavanone 4′-*O*-β-D-(4″-*O*-methyl)-glucopyranoside (3a) (Acetone-d_6, 151 MHz)
Figure S58. HSQC NMR spectrum of flavanone 4′-*O*-β-D-(4″-*O*-methyl)-glucopyranoside (3a) (Acetone-d_6, 151 MHz)
Figure S59. HMBC NMR spectrum of flavanone 4′-*O*-β-D-(4″-*O*-methyl)-glucopyranoside (3a) (Acetone-d_6, 151 MHz)
Figure S60. COSY NMR spectrum of flavanone 4′-*O*-β-D-(4″-*O*-methyl)-glucopyranoside (3a) (Acetone-d_6, 600 MHz)
Figure S61. ^{1}H NMR spectrum of 4′-hydroxyflavanone 6-*O*-β-D-(4″-*O*-methyl)-glucopyranoside (3b) (Acetone-d_6, 600 MHz)
Figure S62. ^{1}H NMR spectrum of 4′-hydroxyflavanone 6-*O*-β-D-(4″-*O*-methyl)-glucopyranoside (3b) (Acetone-d_6, 600 MHz)

Figure S63. ^{13}C NMR spectrum of 4′-hydroxyflavanone 6-*O*-β-D-(4″-*O*-methyl)-glucopyranoside (3b) (Acetone-d_6, 151 MHz)
Figure S64. HSQC NMR spectrum of 4′-hydroxyflavanone 6-*O*-β-D-(4″-*O*-methyl)-glucopyranoside (3b) (Acetone-d_6, 151 MHz)
Figure S65. HSQC NMR spectrum of 4′-hydroxyflavanone 6-*O*-β-D-(4″-*O*-methyl)-glucopyranoside (3b) (Acetone-d_6, 151 MHz)
Figure S66. HSQC NMR spectrum of 4′-hydroxyflavanone 6-*O*-β-D-(4″-*O*-methyl)-glucopyranoside (3b) (Acetone-d_6, 151 MHz)
Figure S67. HMBC NMR spectrum of 4′-hydroxyflavanone 6-*O*-β-D-(4″-*O*-methyl)-glucopyranoside (3b) (Acetone-d_6, 151 MHz)
Figure S68. COSY NMR spectrum of 4′-hydroxyflavanone 6-*O*-β-D-(4″-*O*-methyl)-glucopyranoside (3b) (Acetone-d_6, 600 MHz)
Figure S69. ^{1}H NMR spectrum of 3′,4′-dihydroxyflavanone 6-*O*-β-D-(4″-*O*-methyl)-glucopyranoside (3c) (Acetone-d_6, 600 MHz)
Figure S70. ^{1}H NMR spectrum of 3′,4′-dihydroxyflavanone 6-*O*-β-D-(4″-*O*-methyl)-glucopyranoside (3c) (Acetone-d_6, 600 MHz)
Figure S71. ^{13}C NMR spectrum of 3′,4′-dihydroxyflavanone 6-*O*-β-D-(4″-*O*-methyl)-glucopyranoside (3c) (Acetone-d_6, 151 MHz)
Figure S72. HSQC NMR spectrum of 3′,4′-dihydroxyflavanone 6-*O*-β-D-(4″-*O*-methyl)-glucopyranoside (3c) (Acetone-d_6, 151 MHz)
Figure S73. HSQC NMR spectrum of 3′,4′-dihydroxyflavanone 6-*O*-β-D-(4″-*O*-methyl)-glucopyranoside (3c) (Acetone-d_6, 151 MHz)
Figure S74. HSQC NMR spectrum of 3′,4′-dihydroxyflavanone 6-*O*-β-D-(4″-*O*-methyl)-glucopyranoside (3c) (Acetone-d_6, 151 MHz)
Figure S75. HMBC NMR spectrum of 3′,4′-dihydroxyflavanone 6-*O*-β-D-(4″-*O*-methyl)-glucopyranoside (3c) (Acetone-d_6, 151 MHz)
Figure S76. COSY NMR spectrum of 3′,4′-dihydroxyflavanone 6-*O*-β-D-(4″-*O*-methyl)-glucopyranoside (3c) (Acetone-d_6, 600 MHz)
Figure S77. ^{1}H NMR spectrum of 6-methoxyflavanone (4) (Acetone-d_6, 600 MHz)
Figure S78. ^{1}H NMR spectrum of 6-methoxyflavanone (4) (Acetone-d_6, 600 MHz)
Figure S79. ^{13}C NMR spectrum of 6-methoxyflavanone (4) (Acetone-d_6, 151 MHz)
Figure S80. HSQC NMR spectrum of 6-methoxyflavanone (4) (Acetone-d_6, 151 MHz)
Figure S81. HSQC NMR spectrum of 6-methoxyflavanone (4) (Acetone-d_6, 151 MHz)
Figure S82. HMBC NMR spectrum of 6-methoxyflavanone (4) (Acetone-d_6, 151 MHz)
Figure S83. ^{1}H NMR spectrum of 6-methoxyflavanone 4′-*O*-β-D-(4″-*O*-methyl)-glucopyranoside (4a) (Acetone-d_6, 600 MHz)
Figure S84. ^{1}H NMR spectrum of 6-methoxyflavanone 4′-*O*-β-D-(4″-*O*-methyl)-glucopyranoside (4a) (Acetone-d_6, 600 MHz)
Figure S85. ^{13}C NMR spectrum of 6-methoxyflavanone 4′-*O*-β-D-(4″-*O*-methyl)-glucopyranoside (4a) (Acetone-d_6, 151 MHz)
Figure S86. HSQC NMR spectrum of 6-methoxyflavanone 4′-*O*-β-D-(4″-*O*-methyl)-glucopyranoside (4a) (Acetone-d_6, 151 MHz)
Figure S87. HSQC NMR spectrum of 6-methoxyflavanone 4′-*O*-β-D-(4″-*O*-methyl)-glucopyranoside (4a) (Acetone-d_6, 151 MHz)

Figure S88. HMBC NMR spectrum of 6-methoxyflavanone 4′-*O*-β-D-(4″-*O*-methyl)-glucopyranoside (4a) (Acetone-d_6, 151 MHz)
Figure S89. COSY NMR spectrum of 6-methoxyflavanone 4′-*O*-β-D-(4″-*O*-methyl)-glucopyranoside (4a) (Acetone-d_6, 600 MHz)
Figure S90. ^{1}H NMR spectrum of 3′-hydroxy-6-methoxyflavanone 4′-*O*-β-D-(4″-*O*-methyl)-glucopyranoside (4b) (Acetone-d_6, 600 MHz)
Figure S91. ^{1}H NMR spectrum of 3′-hydroxy-6-methoxyflavanone 4′-*O*-β-D-(4″-*O*-methyl)-glucopyranoside (4b) (Acetone-d_6, 600 MHz)
Figure S92. ^{13}C NMR spectrum of 3′-hydroxy-6-methoxyflavanone 4′-*O*-β-D-(4″-*O*-methyl)-glucopyranoside (4b) (Acetone-d_6, 151 MHz)
Figure S93. HSQC NMR spectrum of 3′-hydroxy-6-methoxyflavanone 4′-*O*-β-D-(4″-*O*-methyl)-glucopyranoside (4b) (Acetone-d_6, 151 MHz)
Figure S94. HSQC NMR spectrum of 3′-hydroxy-6-methoxyflavanone 4′-*O*-β-D-(4″-*O*-methyl)-glucopyranoside (4b) (Acetone-d_6, 151 MHz)
Figure S95. HSQC NMR spectrum of 3′-hydroxy-6-methoxyflavanone 4′-*O*-β-D-(4″-*O*-methyl)-glucopyranoside (4b) (Acetone-d_6, 151 MHz)
Figure S96. HMBC NMR spectrum of 3′-hydroxy-6-methoxyflavanone 4′-*O*-β-D-(4″-*O*-methyl)-glucopyranoside (4b) (Acetone-d_6, 151 MHz)
Figure S97. COSY NMR spectrum of 3′-hydroxy-6-methoxyflavanone 4′-*O*-β-D-(4″-*O*-methyl)-glucopyranoside (4b) (Acetone-d_6, 600 MHz)
Figure S98. ^{1}H NMR spectrum of 6-methoxyflavone (5) (Acetone-d_6, 600 MHz)
Figure S99. ^{13}C NMR spectrum of 6-methoxyflavone (5) (Acetone-d_6, 151 MHz)
Figure S100. HSQC NMR spectrum of 6-methoxyflavone (5) (Acetone-d_6, 151 MHz)
Figure S101. HMBC NMR spectrum of 6-methoxyflavone (5) (Acetone-d_6, 151 MHz)
Figure S102. COSY NMR spectrum of 6-methoxyflavone (5) (Acetone-d_6, 600 MHz)
Figure S103. ^{1}H NMR spectrum of 6-methoxyflavone 3′-*O*-β-D-(4″-*O*-methyl)-glucopyranoside (5a) (Acetone-d_6, 600 MHz)
Figure S104. ^{1}H NMR spectrum of 6-methoxyflavone 3′-*O*-β-D-(4″-*O*-methyl)-glucopyranoside (5a) (Acetone-d_6, 600 MHz)
Figure S105. ^{13}C NMR spectrum of 6-methoxyflavone 3′-*O*-β-D-(4″-*O*-methyl)-glucopyranoside (5a) (Acetone-d_6, 151 MHz)
Figure S106. HSQC NMR spectrum of 6-methoxyflavone 3′-*O*-β-D-(4″-*O*-methyl)-glucopyranoside (5a) (Acetone-d_6, 151 MHz)
Figure S107. HSQC NMR spectrum of 6-methoxyflavone 3′-*O*-β-D-(4″-*O*-methyl)-glucopyranoside (5a) (Acetone-d_6, 151 MHz)
Figure S108. HMBC NMR spectrum of 6-methoxyflavone 3′-*O*-β-D-(4″-*O*-methyl)-glucopyranoside (5a) (Acetone-d_6, 151 MHz)

Figure S109. COSY NMR spectrum of 6-methoxyflavone 3′-*O*-β-D-(4″-*O*-methyl)-glucopyranoside (5a) (Acetone-d_6, 600 MHz)
Figure S110. ^{1}H NMR spectrum of 6-methoxyflavone 4′-*O*-β-D-(4″-*O*-methyl)-glucopyranoside (5b) (Acetone-d_6, 600 MHz)
Figure S111. ^{1}H NMR spectrum of 6-methoxyflavone 4′-*O*-β-D-(4″-*O*-methyl)-glucopyranoside (5b) (Acetone-d_6, 600 MHz)
Figure S112. ^{13}C NMR spectrum of 6-methoxyflavone 4′-*O*-β-D-(4″-*O*-methyl)-glucopyranoside (5b) (Acetone-d_6, 151 MHz)
Figure S113. HSQC NMR spectrum of 6-methoxyflavone 4′-*O*-β-D-(4″-*O*-methyl)-glucopyranoside (5b) (Acetone-d_6, 151 MHz)
Figure S114. HSQC NMR spectrum of 6-methoxyflavone 4′-*O*-β-D-(4″-*O*-methyl)-glucopyranoside (5b) (Acetone-d_6, 151 MHz)
Figure S115. HMBC NMR spectrum of 6-methoxyflavone 4′-*O*-β-D-(4″-*O*-methyl)-glucopyranoside (5b) (Acetone-d_6, 151 MHz)
Figure S116. COSY NMR spectrum of 6-methoxyflavone 4′-*O*-β-D-(4″-*O*-methyl)-glucopyranoside (5b) (Acetone-d_6, 600 MHz)
Figure S117. ^{1}H NMR spectrum of 3′-hydroxy-6-methoxyflavone 4′-*O*-β-D-(4″-*O*-methyl)-glucopyranoside (5c) (Acetone-d_6, 600 MHz)
Figure S118. ^{1}H NMR spectrum of 3′-hydroxy-6-methoxyflavone 4′-*O*-β-D-(4″-*O*-methyl)-glucopyranoside (5c) (Acetone-d_6, 600 MHz)
Figure S119. ^{13}C NMR spectrum of 3′-hydroxy-6-methoxyflavone 4′-*O*-β-D-(4″-*O*-methyl)-glucopyranoside (5c) (Acetone-d_6, 151 MHz)
Figure S120. HSQC NMR spectrum of 3′-hydroxy-6-methoxyflavone 4′-*O*-β-D-(4″-*O*-methyl)-glucopyranoside (5c) (Acetone-d_6, 151 MHz)
Figure S121. HSQC NMR spectrum of 3′-hydroxy-6-methoxyflavone 4′-*O*-β-D-(4″-*O*-methyl)-glucopyranoside (5c) (Acetone-d_6, 151 MHz)
Figure S122. HMBC NMR spectrum of 3′-hydroxy-6-methoxyflavone 4′-*O*-β-D-(4″-*O*-methyl)-glucopyranoside (5c) (Acetone-d_6, 151 MHz)
Figure S123. COSY NMR spectrum of 3′-hydroxy-6-methoxyflavone 4′-*O*-β-D-(4″-*O*-methyl)-glucopyranoside (5c) (Acetone-d_6, 600 MHz)

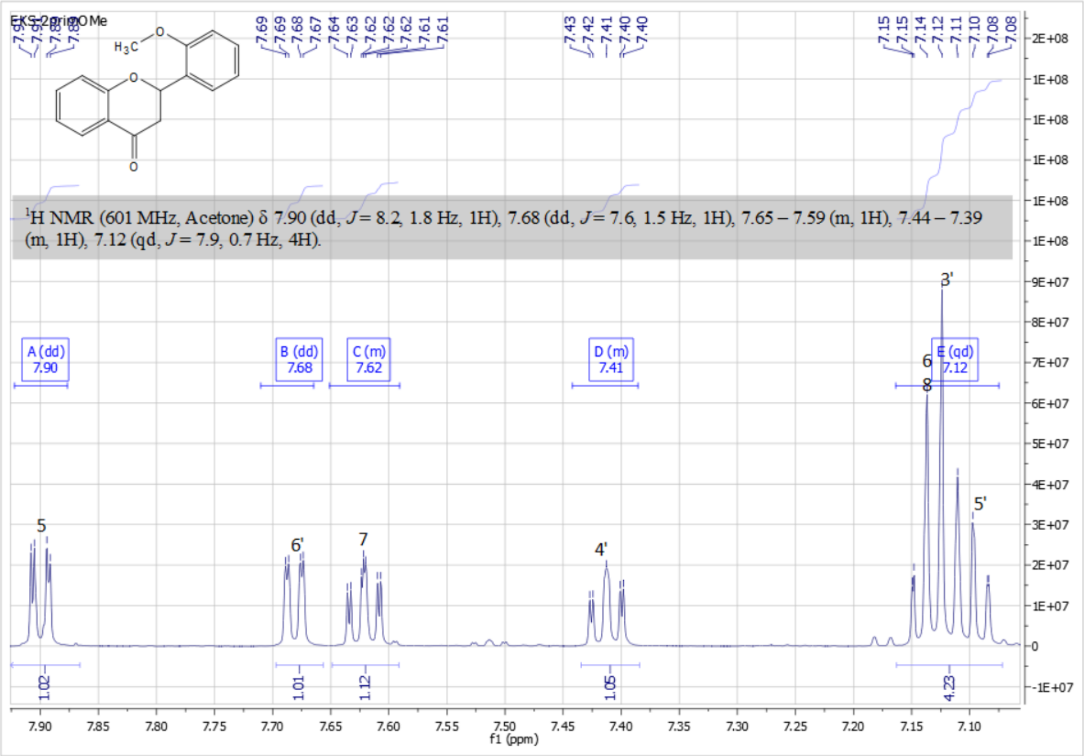

Figure S1. ^{1}H NMR spectrum of 2′-methoxyflavanone(1) (Acetone-d_6, 600 MHz)

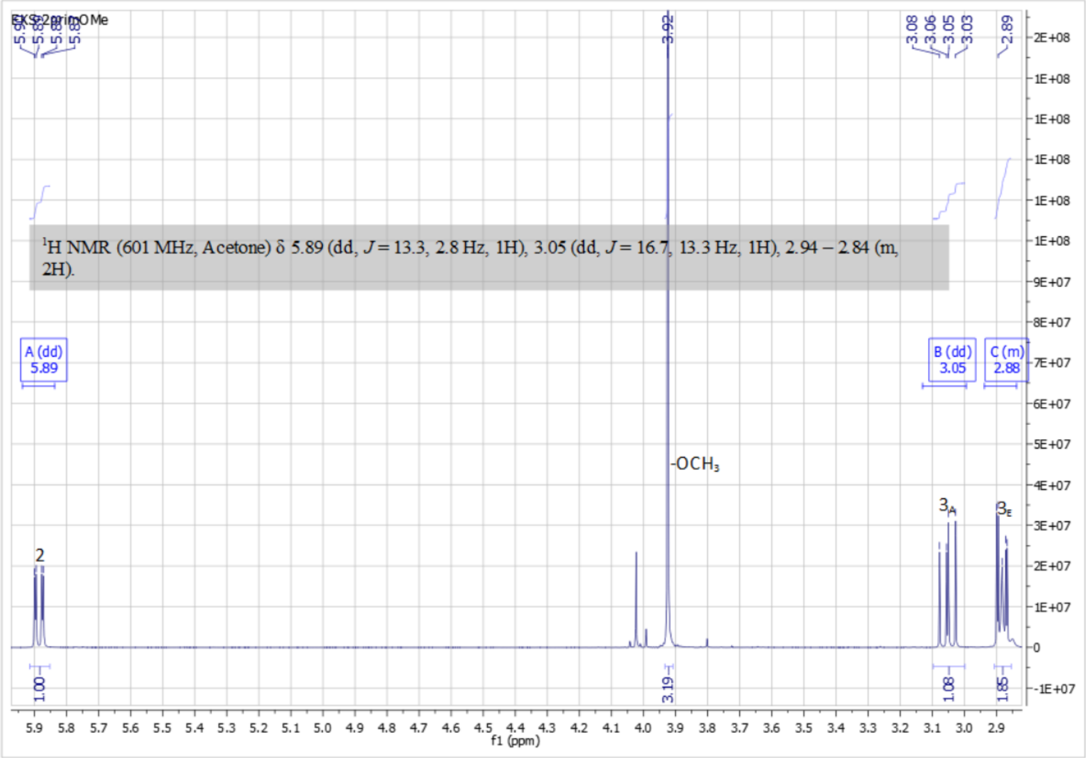

Figure S2. ^{1}H NMR spectrum of 2′-methoxyflavanone(1) (Acetone-d_6, 600 MHz)

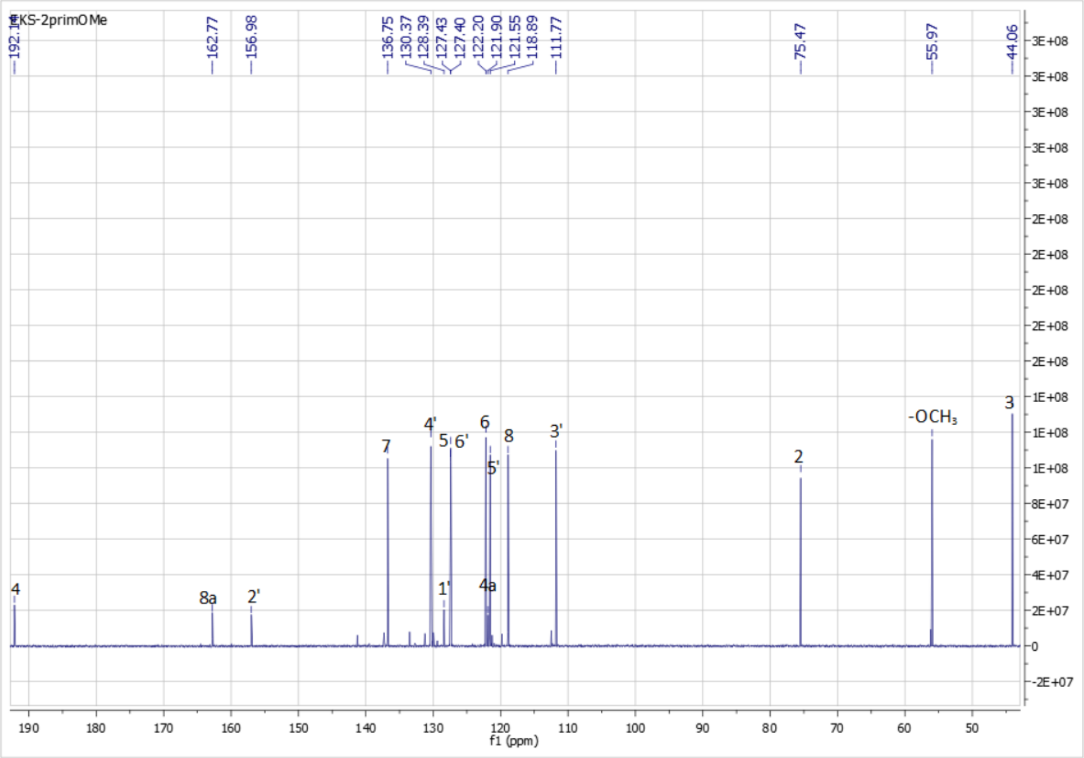

Figure S3. ^{13}C NMR spectrum of 2′-methoxyflavanone (1) (Acetone-d6, 151 MHz)

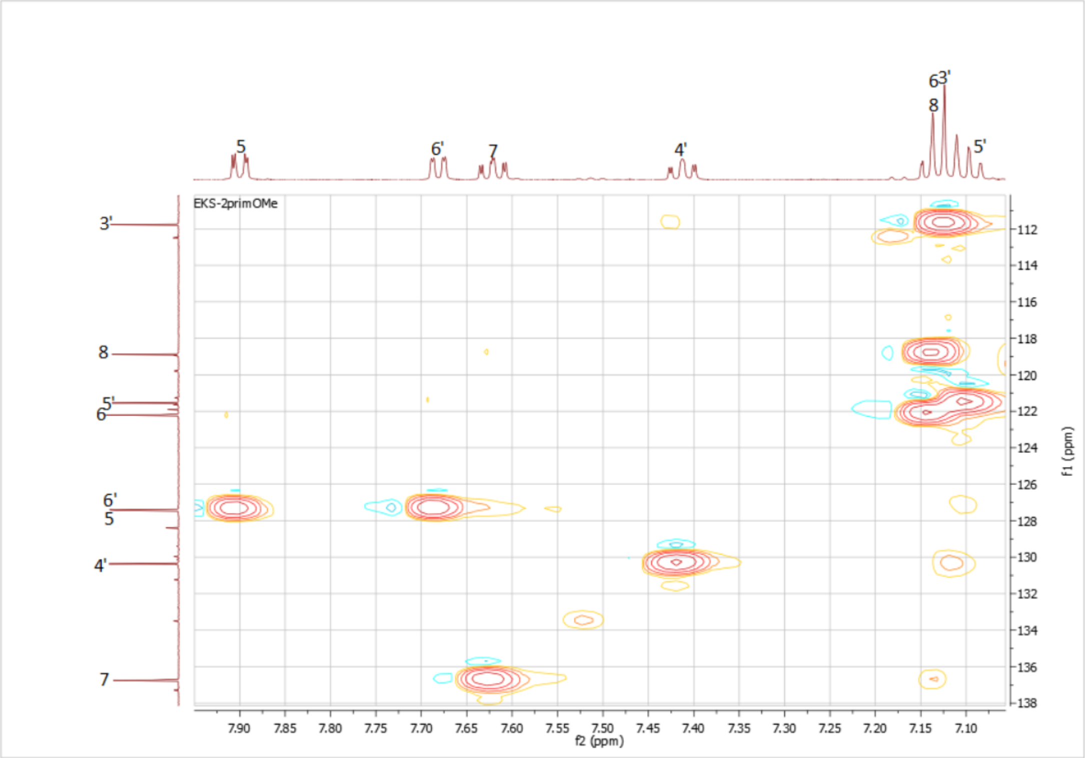

Figure S4. HSQC NMR spectrum of 2′-methoxyflavanone (1) (Acetone-d6, 151 MHz)

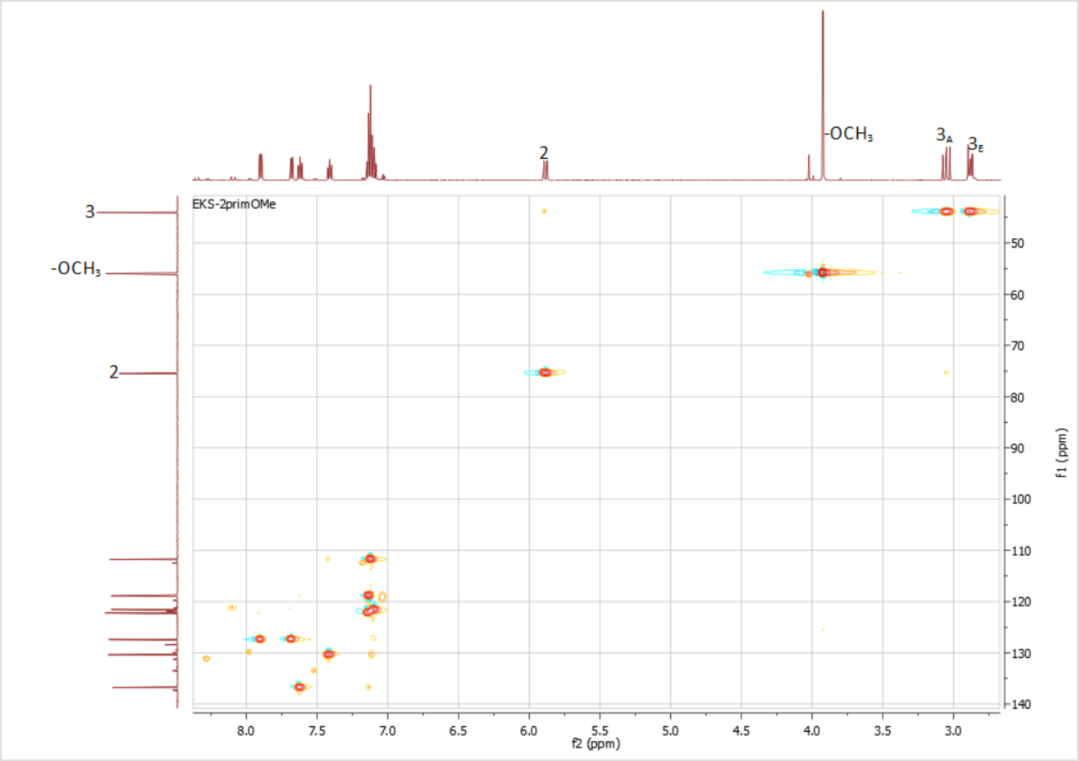

Figure S5. HSQC NMR spectrum of 2′-methoxyflavanone (1) (Acetone-d6, 151 MHz)

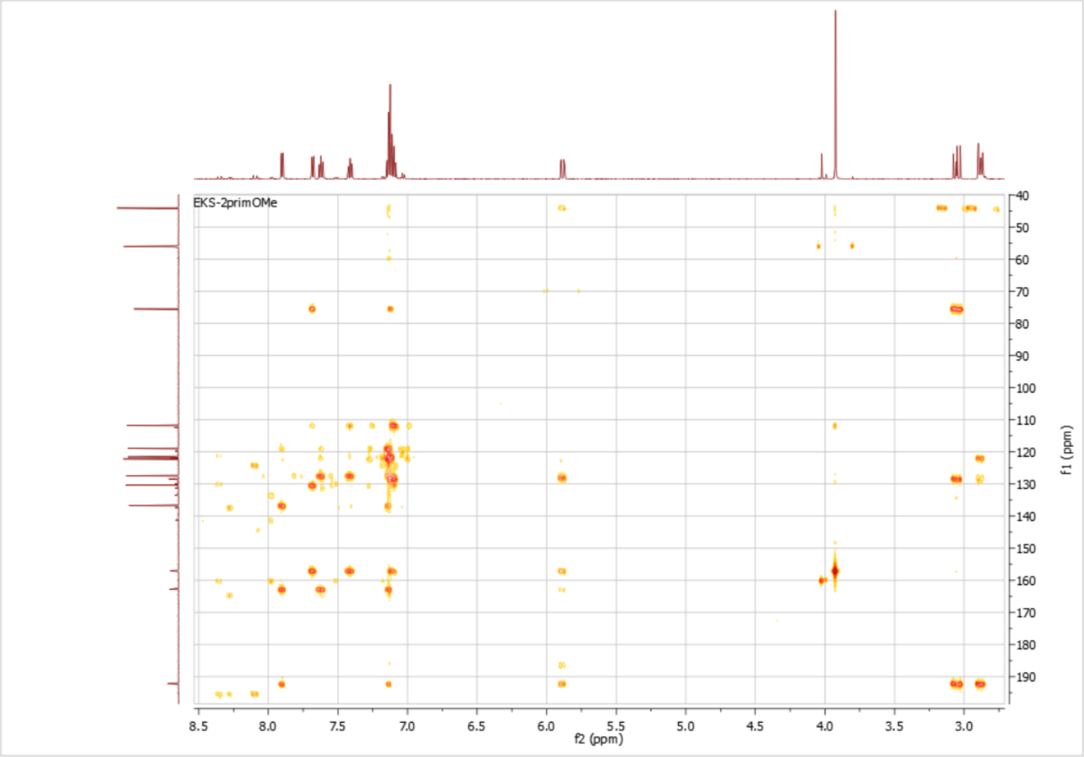

Figure S6. HMBC NMR spectrum of 2′-methoxyflavanone (1) (Acetone-d6, 151 MHz)

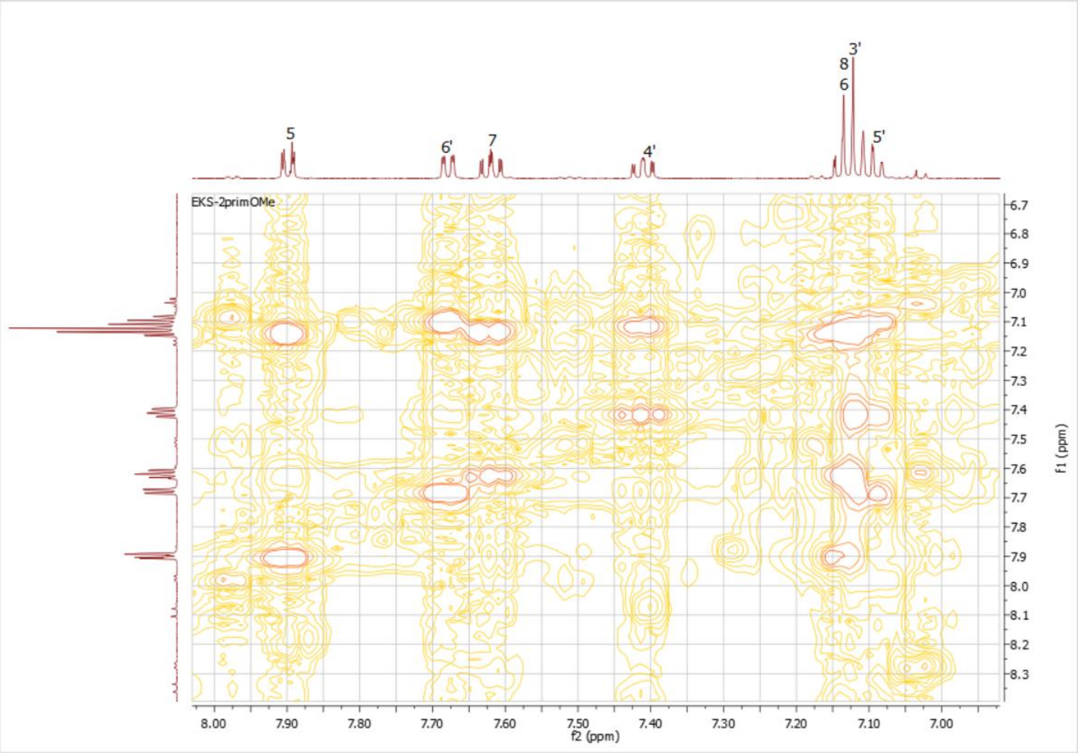

Figure S7. COSY NMR spectrum of 2′-methoxyflavanone (1) (Acetone-d_6, 600 MHz)

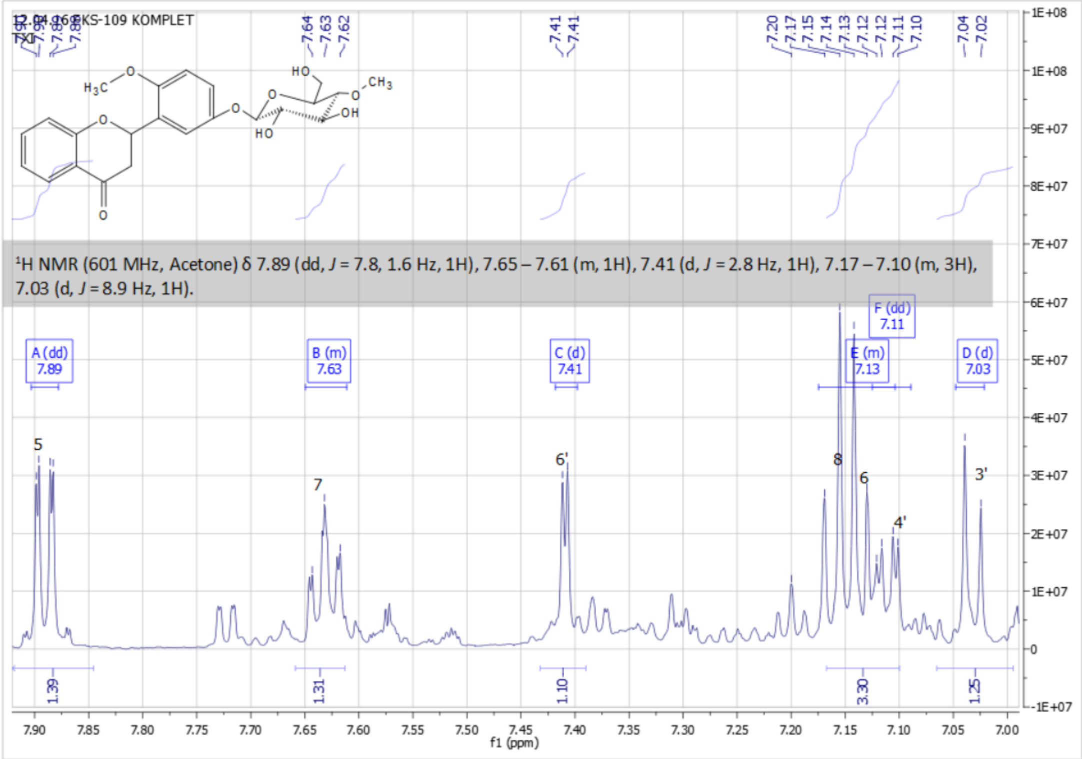

Figure S8. ^{1}H NMR spectrum of 2′-methoxyflavanone 5′-*O*-β-D-(4″-*O*-methyl)-glucopyranoside (1a) (Acetone-d_6, 600 MHz)

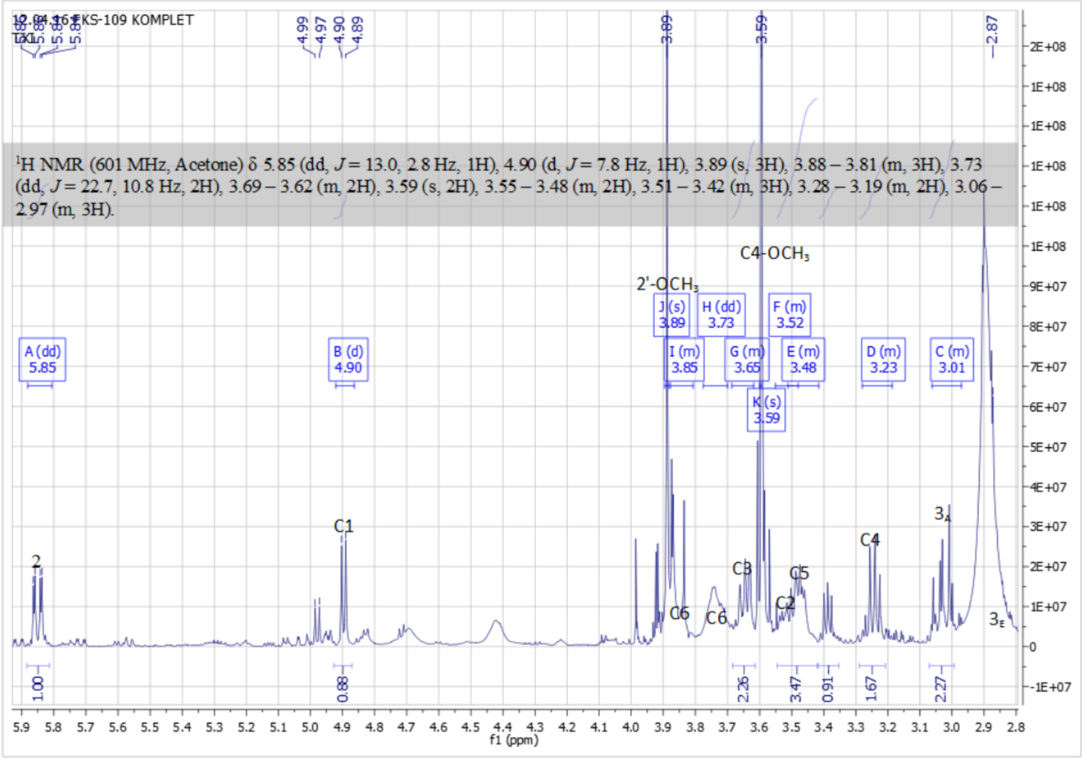

Figure S9. ^{1}H NMR spectrum of 2′-methoxyflavanone 5′-*O*-β-D-(4″-*O*-methyl)-glucopyranoside (1a) (Acetone-d$_6$, 600 MHz)

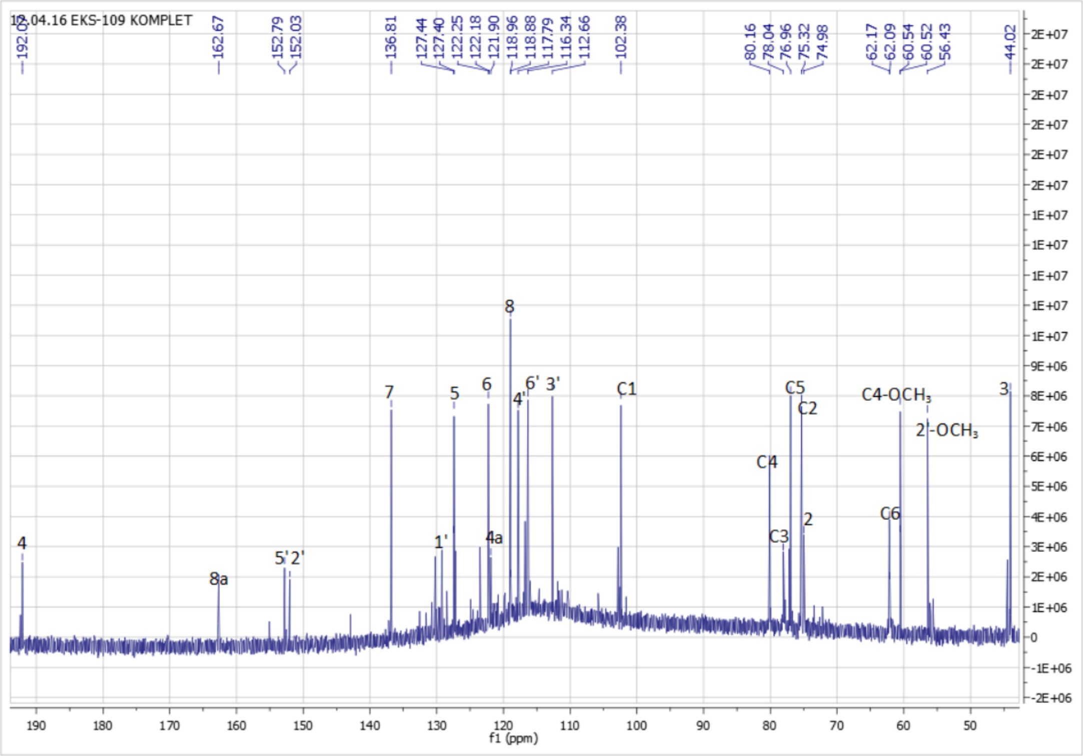

Figure S10. ^{13}C NMR spectrum of 2′-methoxyflavanone 5′-*O*-β-D-(4″-*O*-methyl)-glucopyranoside (1a) (Acetone-d$_6$, 151 MHz)

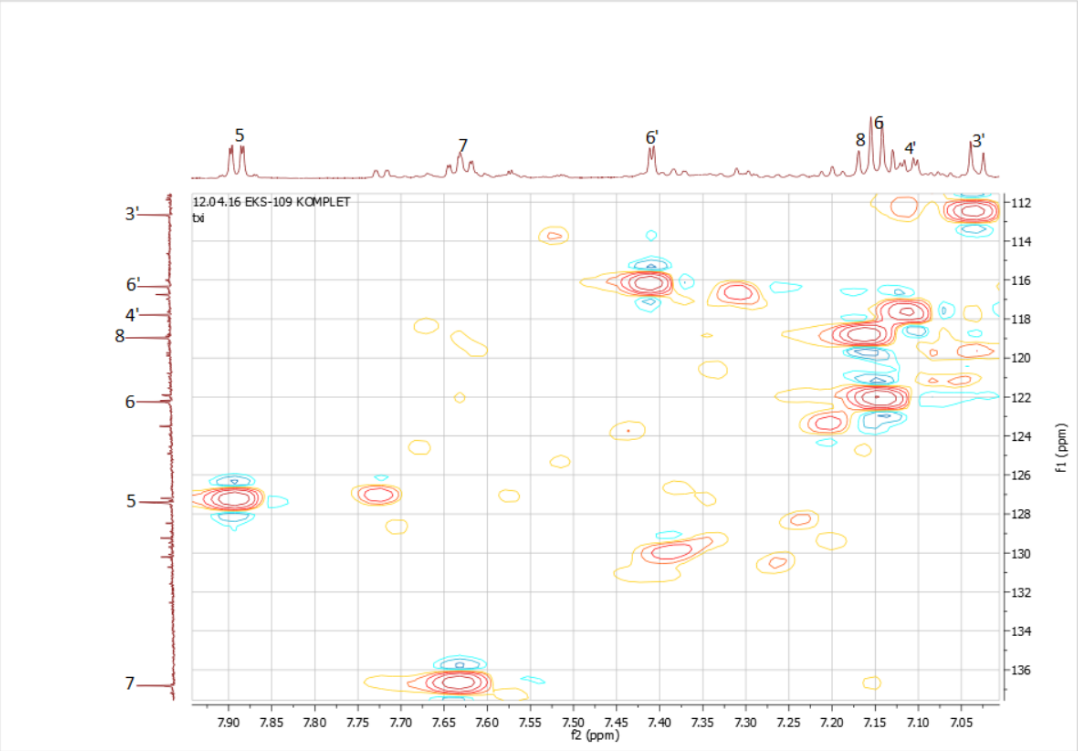

Figure S11. HSQC NMR spectrum of 2′-methoxyflavanone 5′-*O*-β-D-(4″-*O*-methyl)-glucopyranoside (1a) (Acetone-d6, 151 MHz)

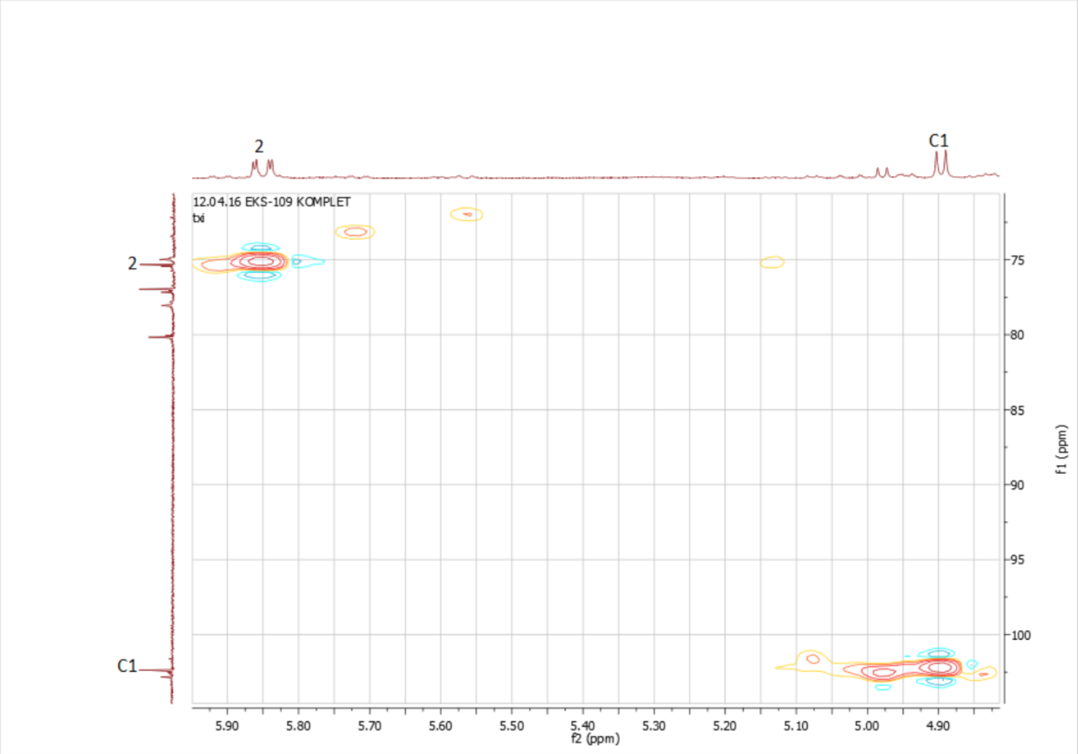

Figure S12. HSQC NMR spectrum of 2′-methoxyflavanone 5′-*O*-β-D-(4″-*O*-methyl)-glucopyranoside (1a) (Acetone-d6, 151 MHz)

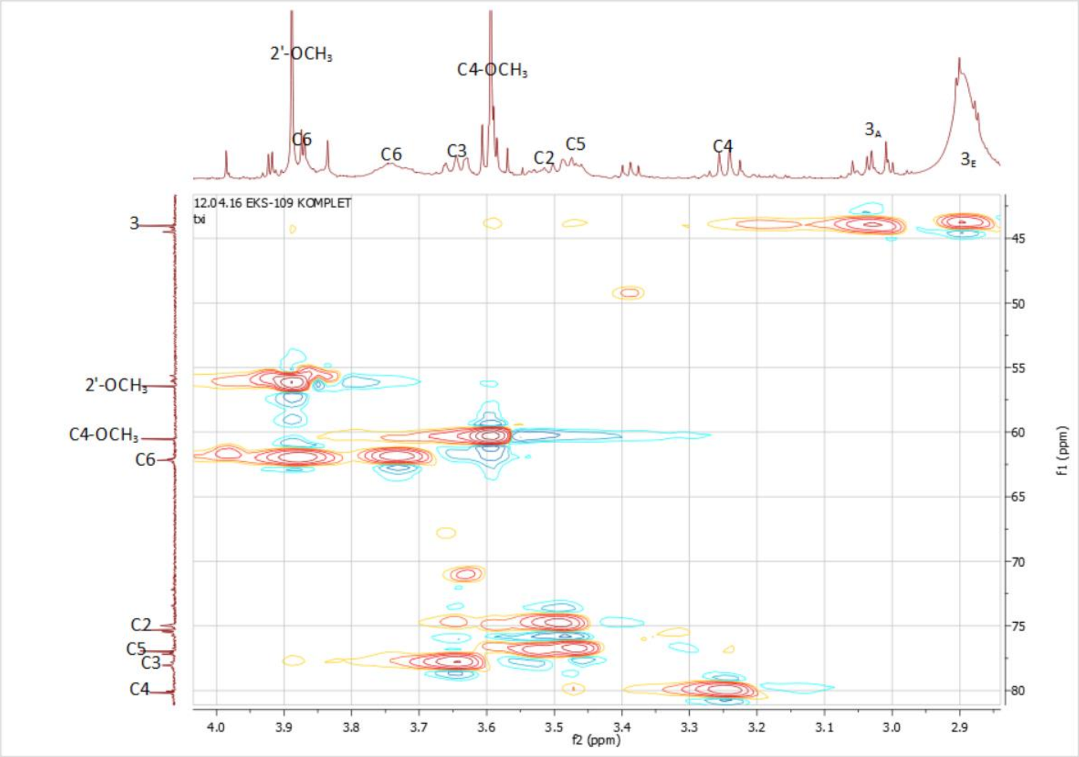

Figure S13. HSQC NMR spectrum of 2′-methoxyflavanone 5′-*O*-β-D-(4″-*O*-methyl)-glucopyranoside (1a) (Acetone-d₆, 151 MHz)

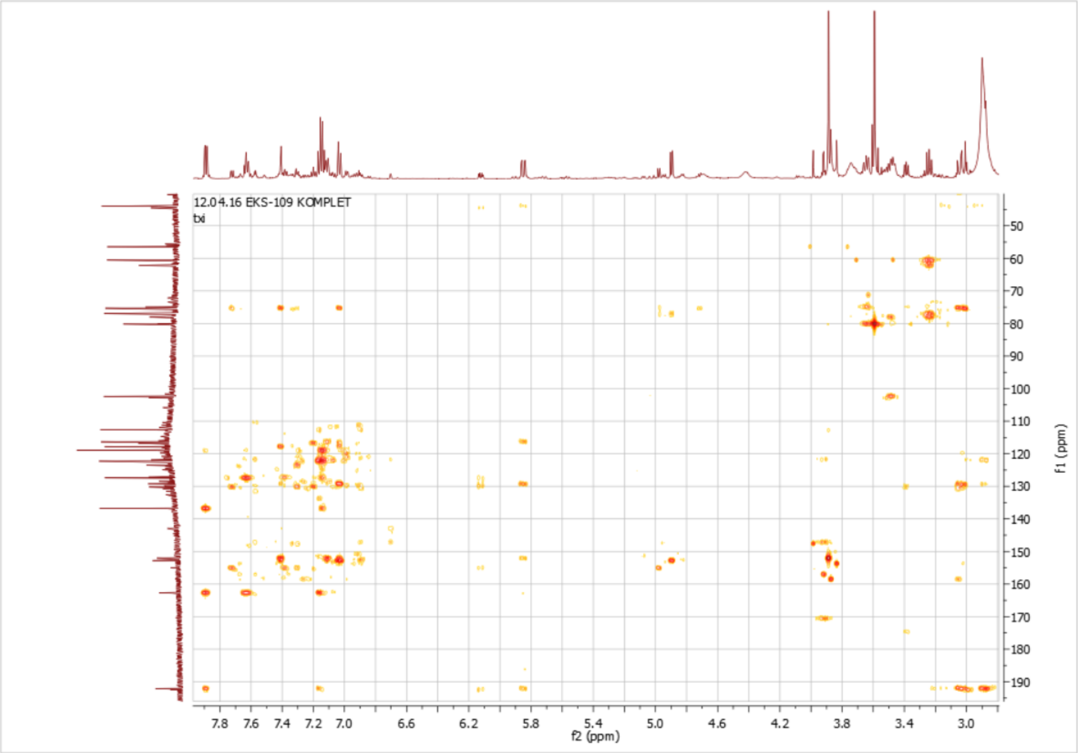

Figure S14. HMBC NMR spectrum 2′-methoxyflavanone 5′-*O*-β-D-(4″-*O*-methyl)-glucopyranoside (1a) (Acetone-d₆, 151 MHz)

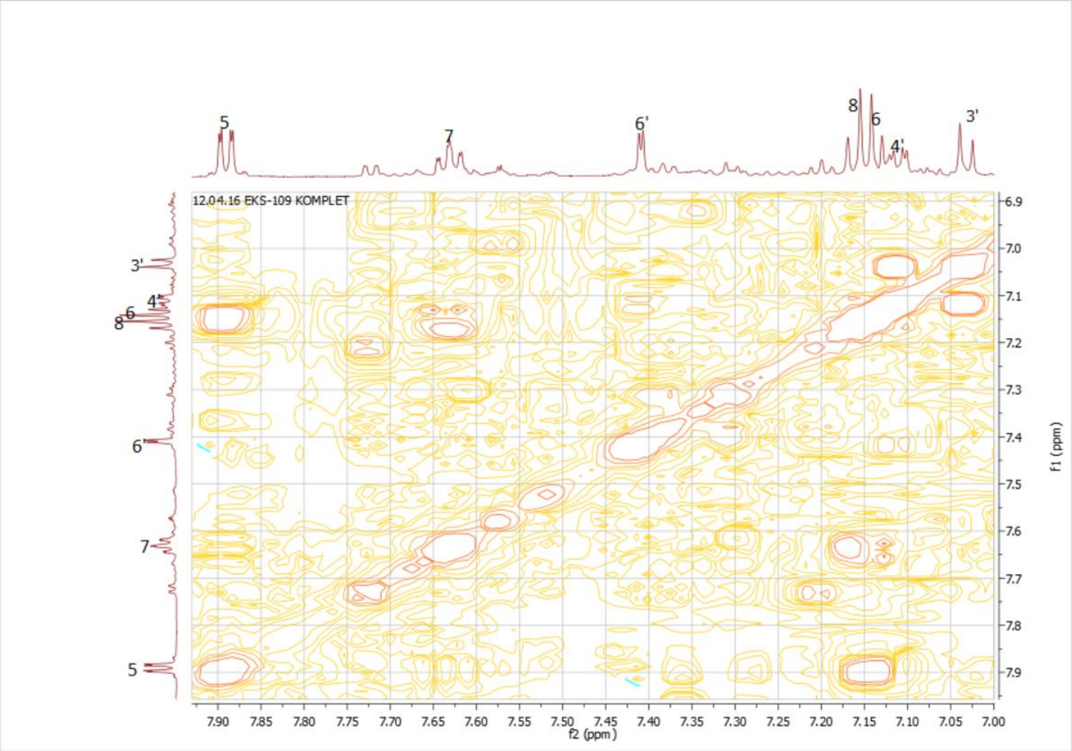

Figure S15. COSY NMR spectrum of 2′-methoxyflavanone 5′-*O*-β-D-(4″-*O*-methyl)-glucopyranoside (1a) (Acetone-d$_6$, 600 MHz)

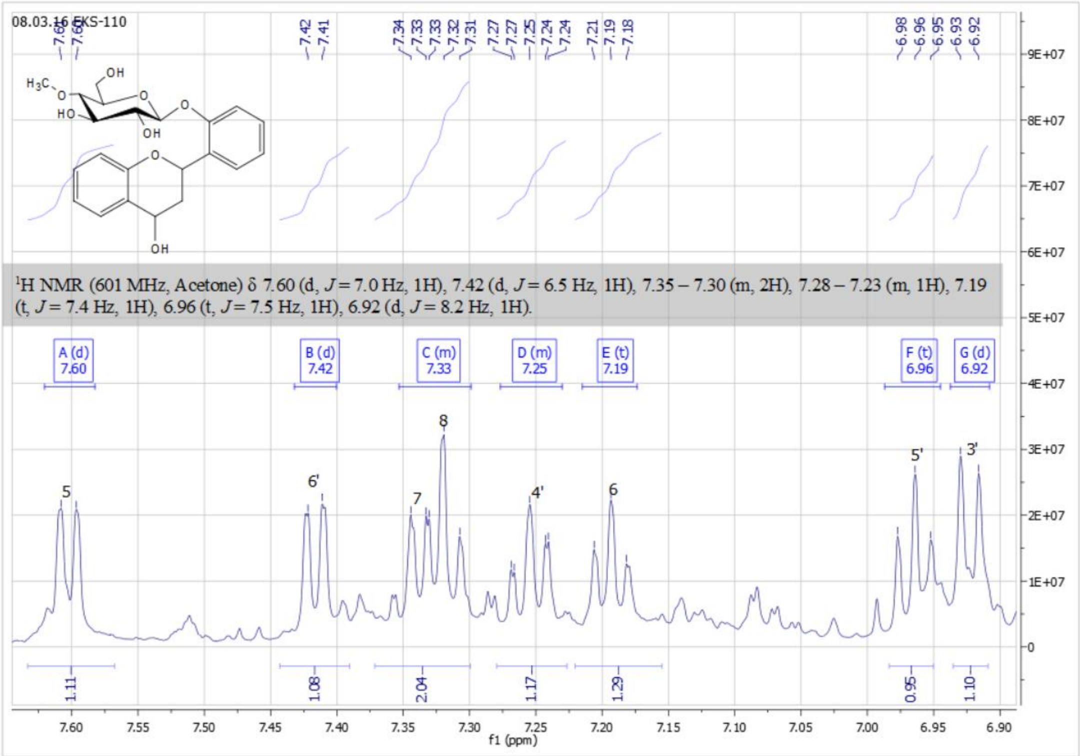

Figure S16. ¹H NMR spectrum of flavan-4-ol 2′-*O*-β-D-(4″-*O*-methyl)-glucopyranoside (1b) (Acetone-d$_6$, 600 MHz)

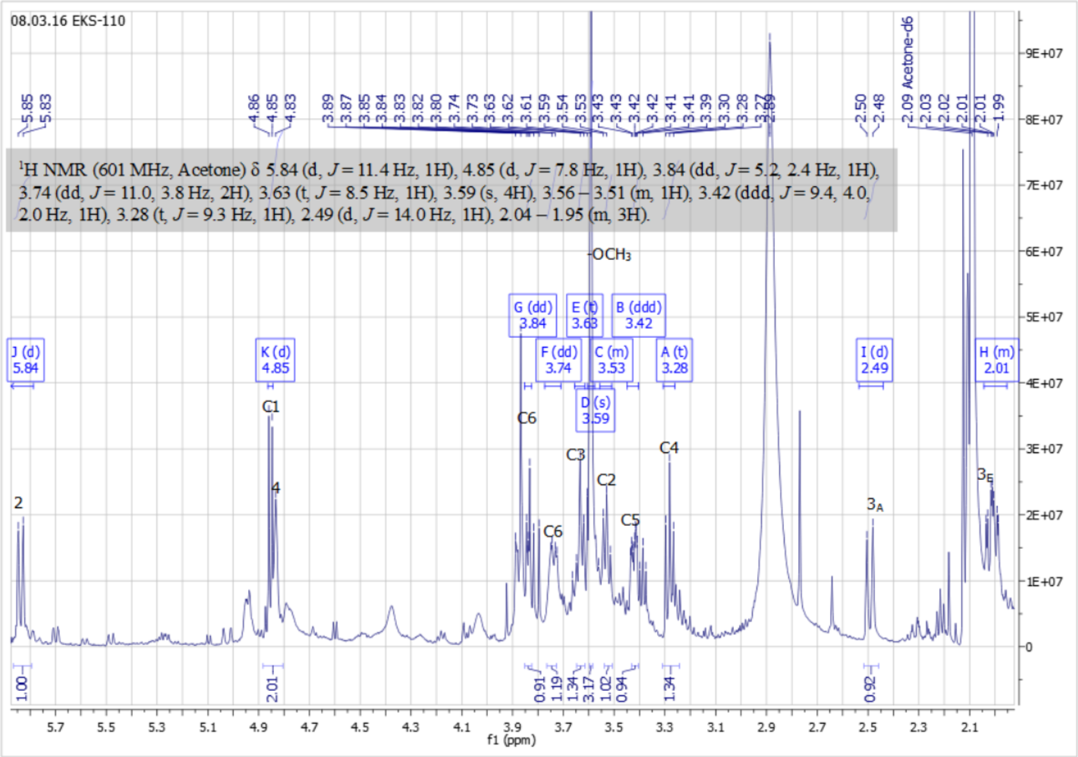

Figure S17. ^{1}H NMR spectrum of flavan-4-ol 2′-*O*-β-D-(4″-*O*-methyl)-glucopyranoside (1b) (Acetone-d_6, 600 MHz)

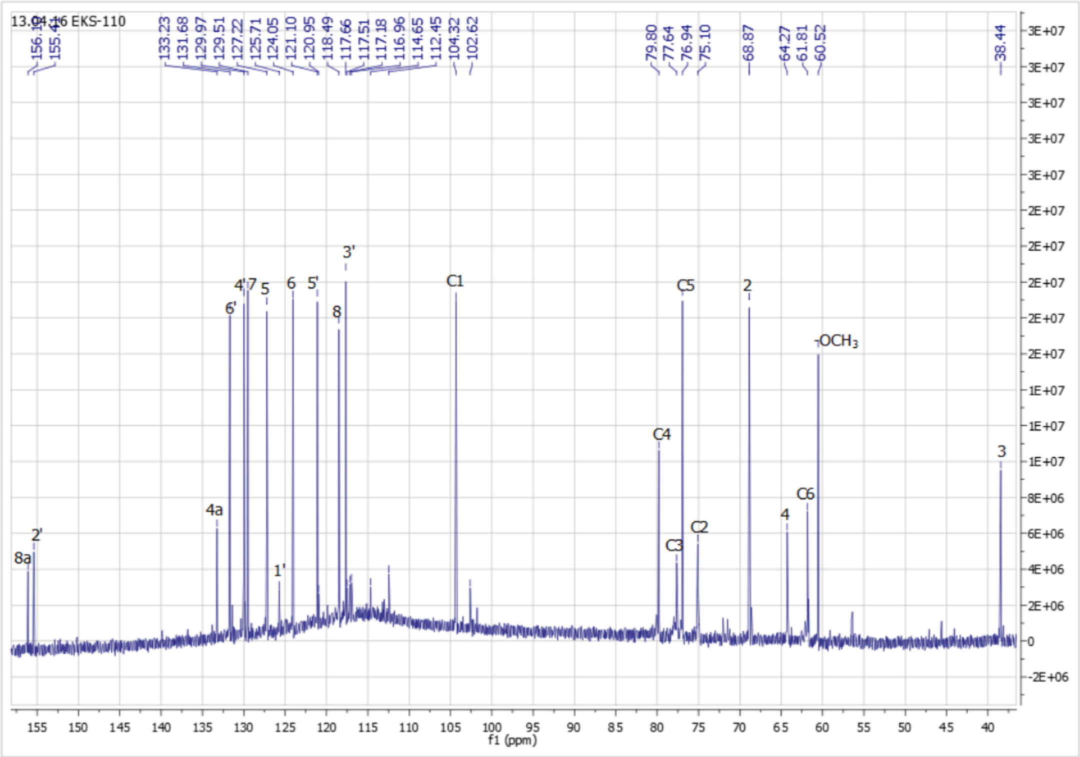

Figure S18. ^{13}C NMR spectrum of flavan-4-ol 2′-*O*-β-D-(4″-*O*-methyl)-glucopyranoside (1b) (Acetone-d_6, 151 MHz)

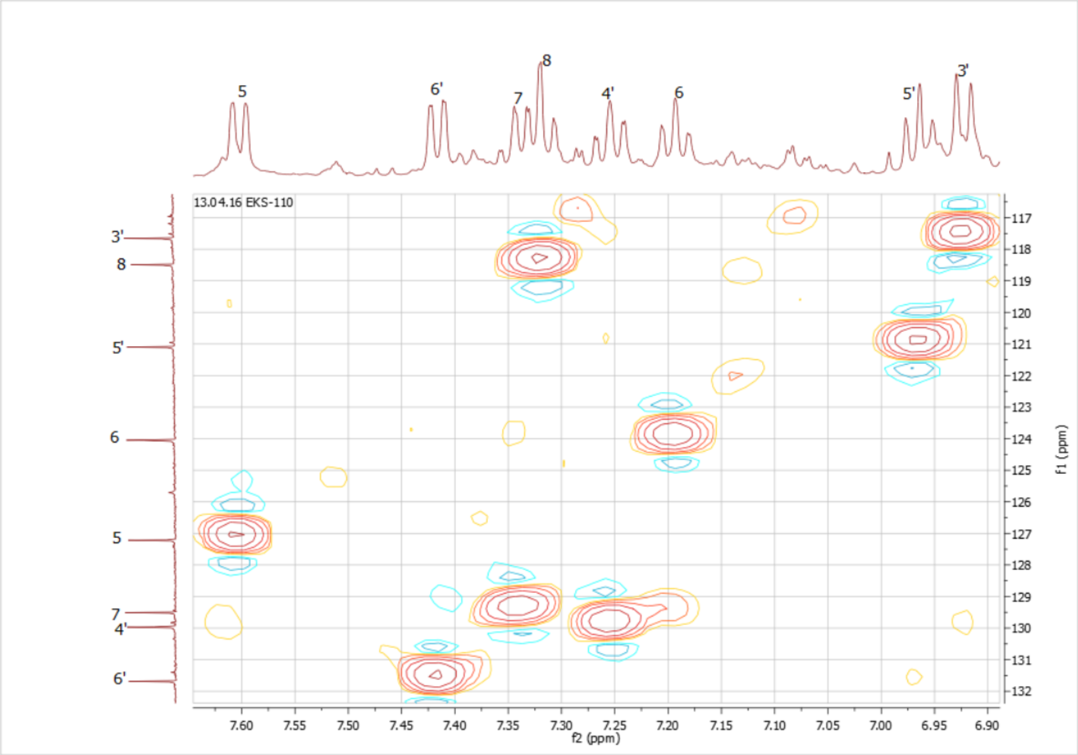

Figure S19. HSQC NMR spectrum of flavan-4-ol 2′-*O*-β-D-(4′′-*O*-methyl)-glucopyranoside (1b) (Acetone-d_6, 151 MHz)

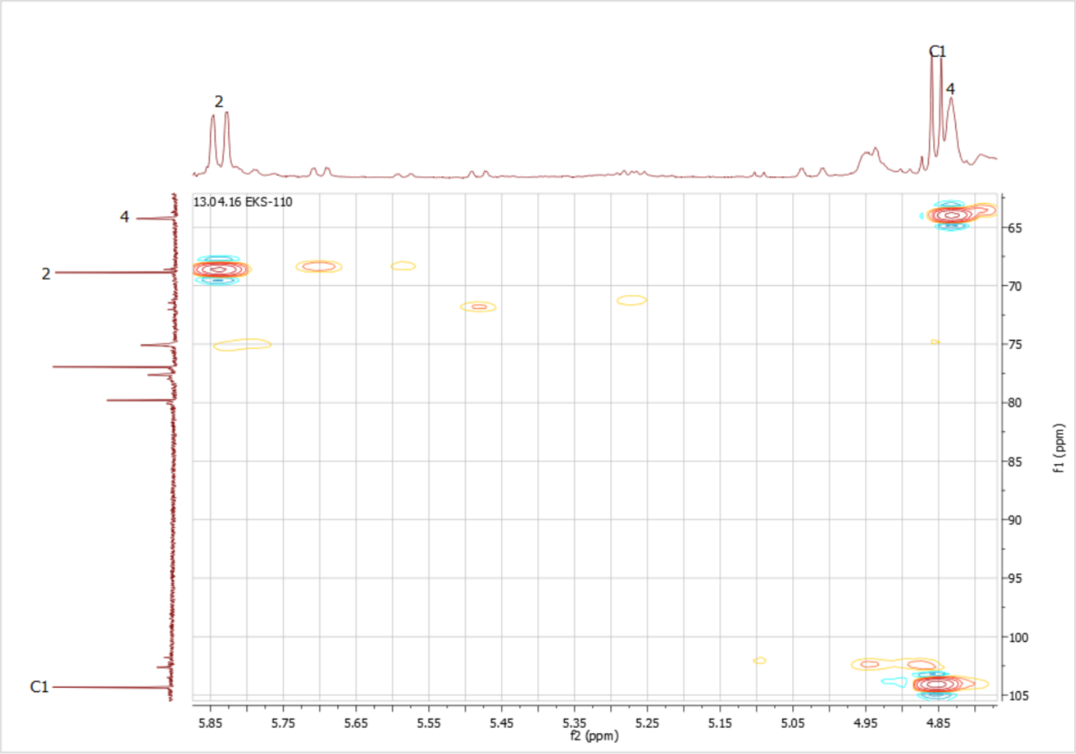

Figure S20. HSQC NMR spectrum of flavan-4-ol 2′-*O*-β-D-(4′′-*O*-methyl)-glucopyranoside (1b) (Acetone-d_6, 151 MHz)

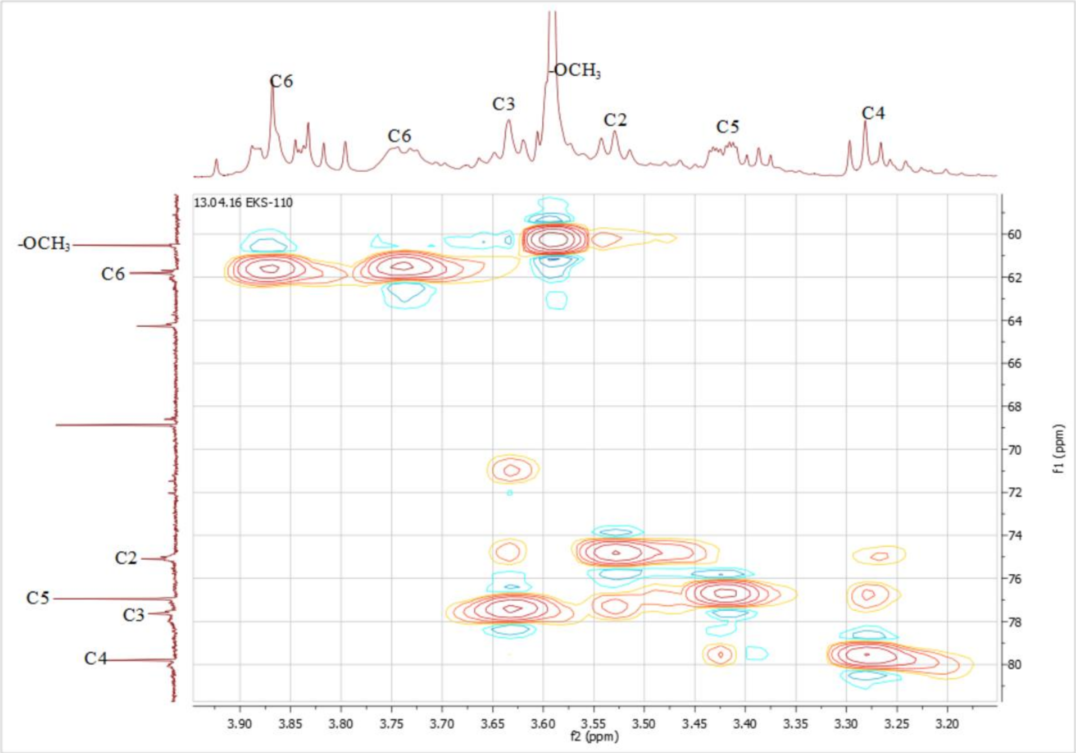

Figure S21. HSQC NMR spectrum of flavan-4-ol 2′-*O*-β-D-(4′′-*O*-methyl)-glucopyranoside (1b) (Acetone-d6, 151 MHz)

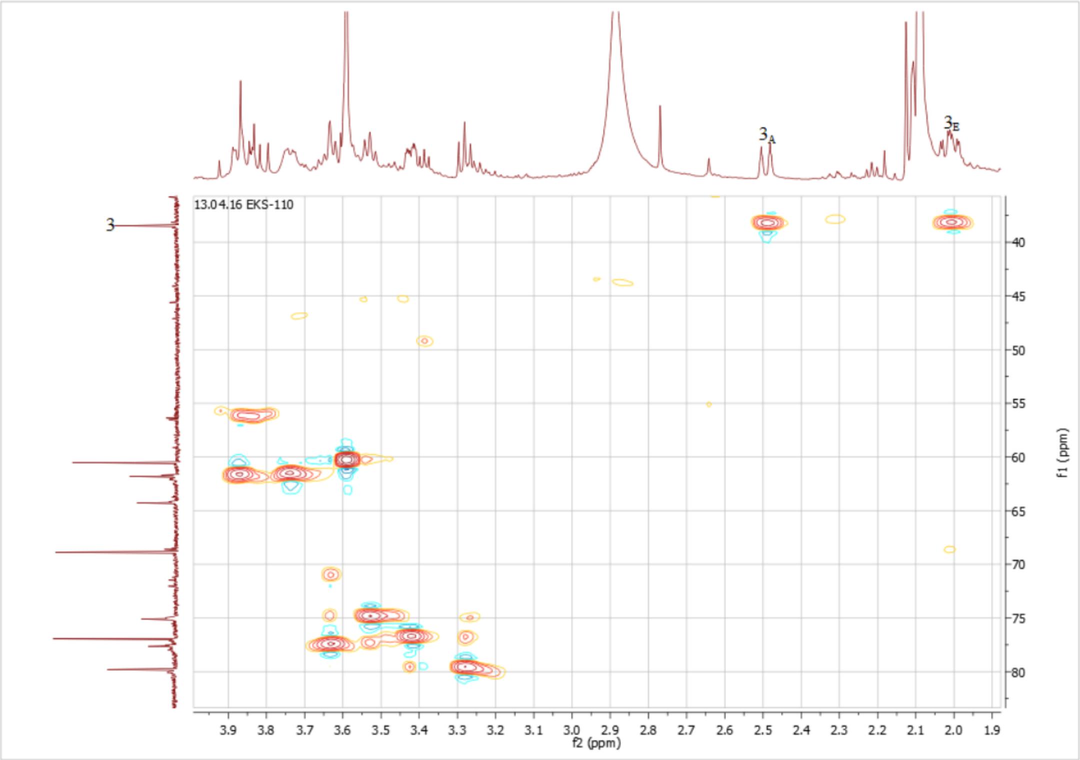

Figure S22. HSQC NMR spectrum of flavan-4-ol 2′-*O*-β-D-(4′′-*O*-methyl)-glucopyranoside (1b) (Acetone-d6, 151 MHz)

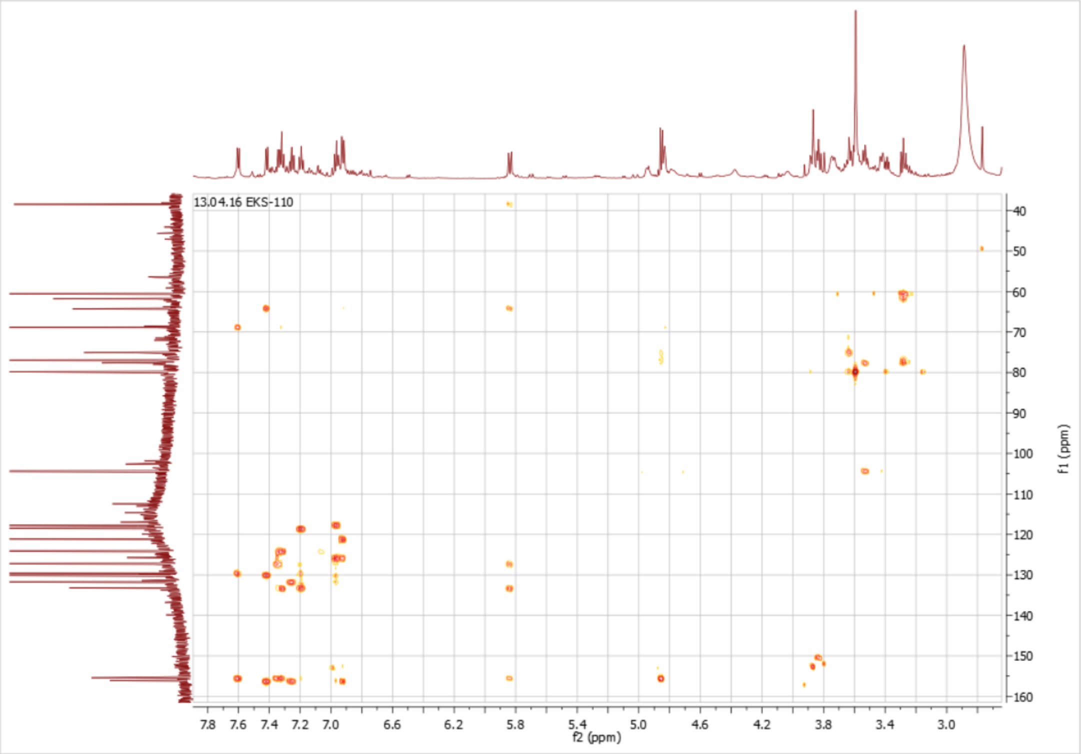

Figure S23. HMBC NMR spectrum of flavan-4-ol 2′-*O*-β-D-(4″-*O*-methyl)-glucopyranoside (1b) (Acetone-d6, 151 MHz)

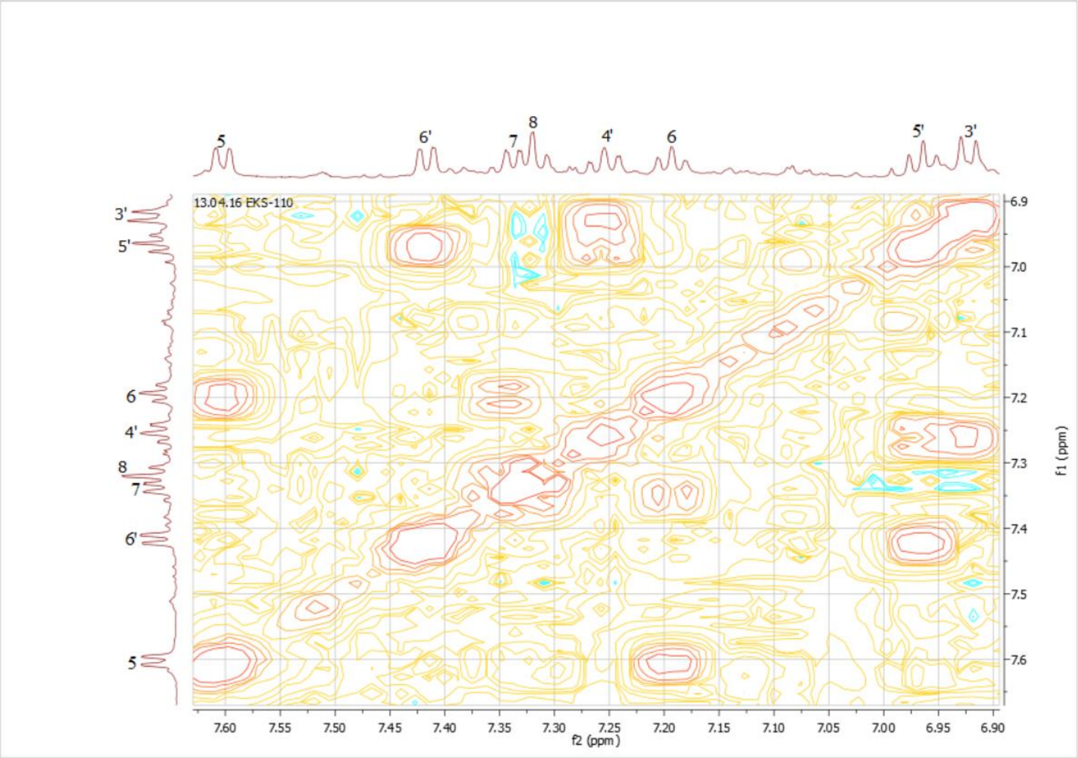

Figure S24. COSY NMR spectrum of flavan-4-ol 2′-*O*-β-D-(4″-*O*-methyl)-glucopyranoside (1b) (Acetone-d6, 600 MHz)

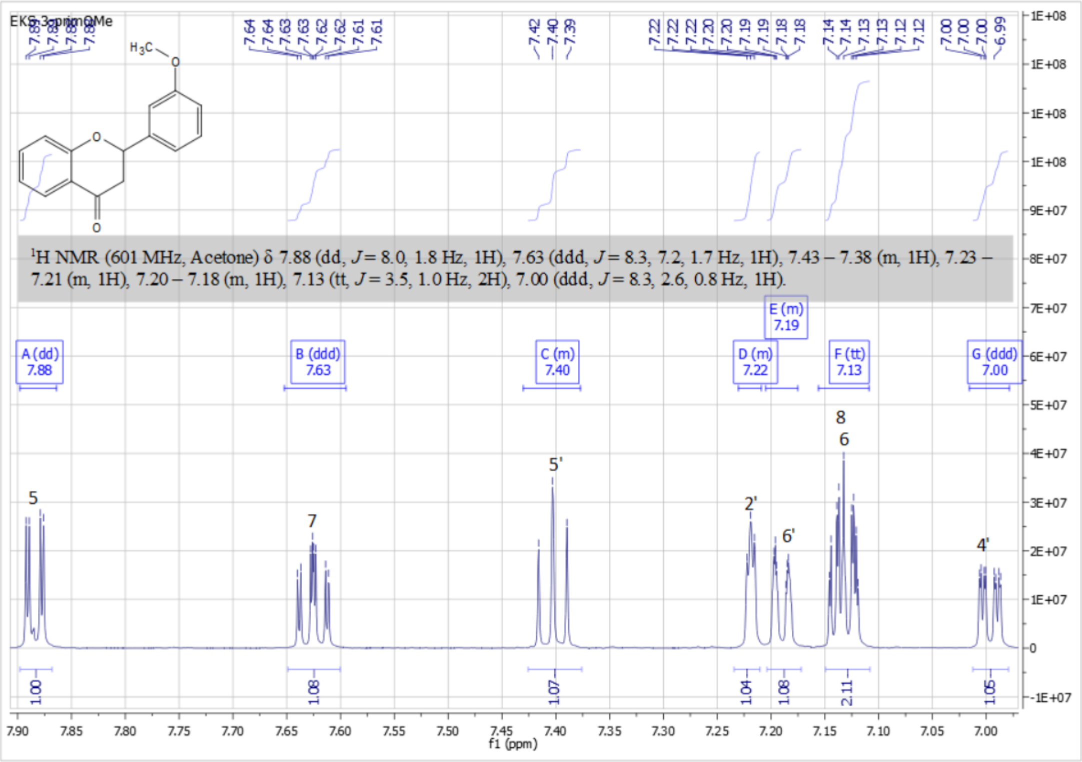

Figure S25. ¹H NMR spectrum of 3′-methoxyflavanone (2) (Acetone-d₆, 600 MHz)

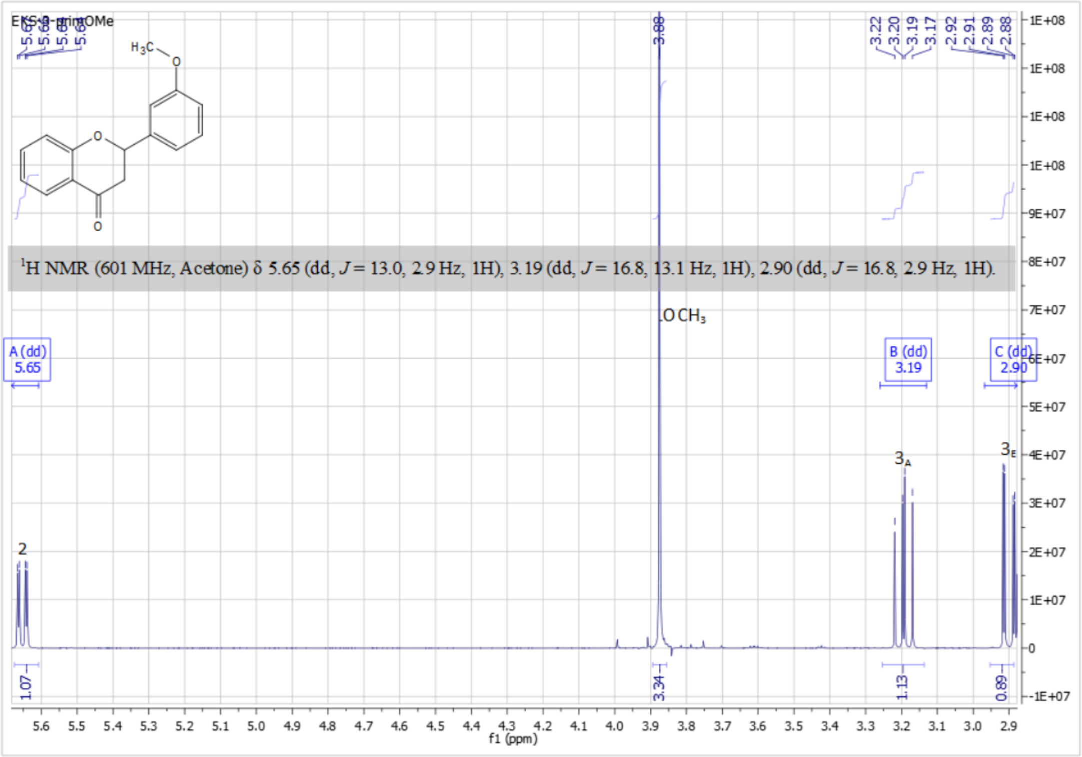

Figure S26. ¹H NMR spectrum of 3′-methoxyflavanone (2) (Acetone-d₆, 600 MHz)

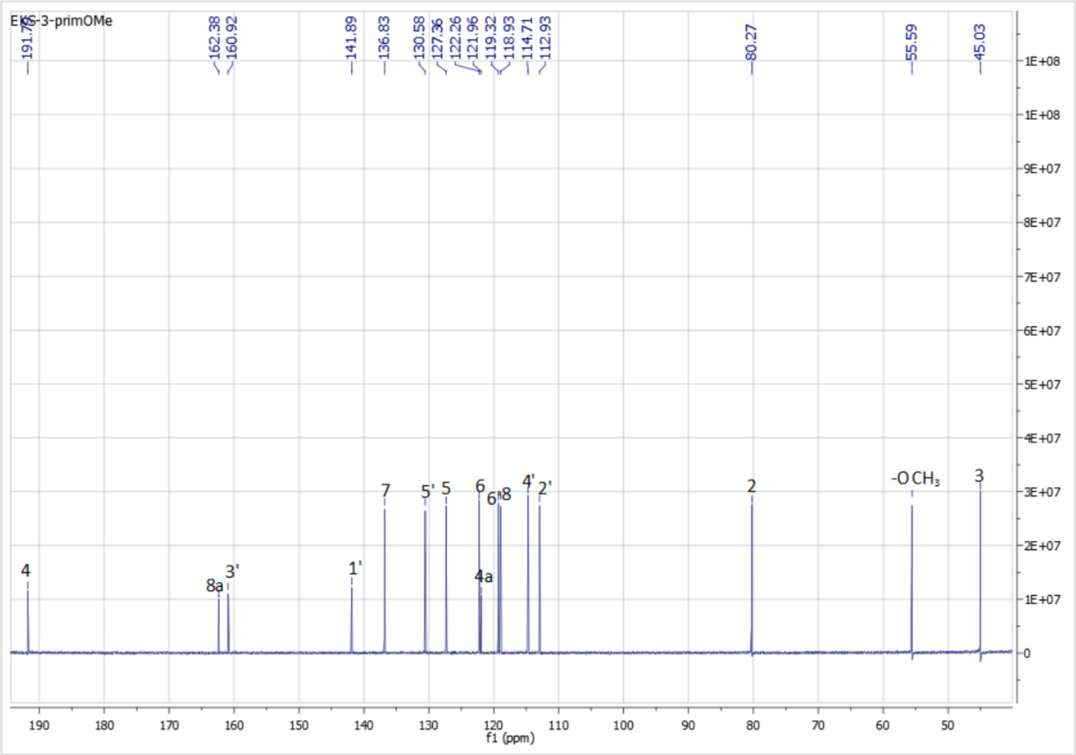

Figure S27. ^{13}C NMR spectrum of 3′-methoxyflavanone (2) (Acetone-d$_6$, 151 MHz)

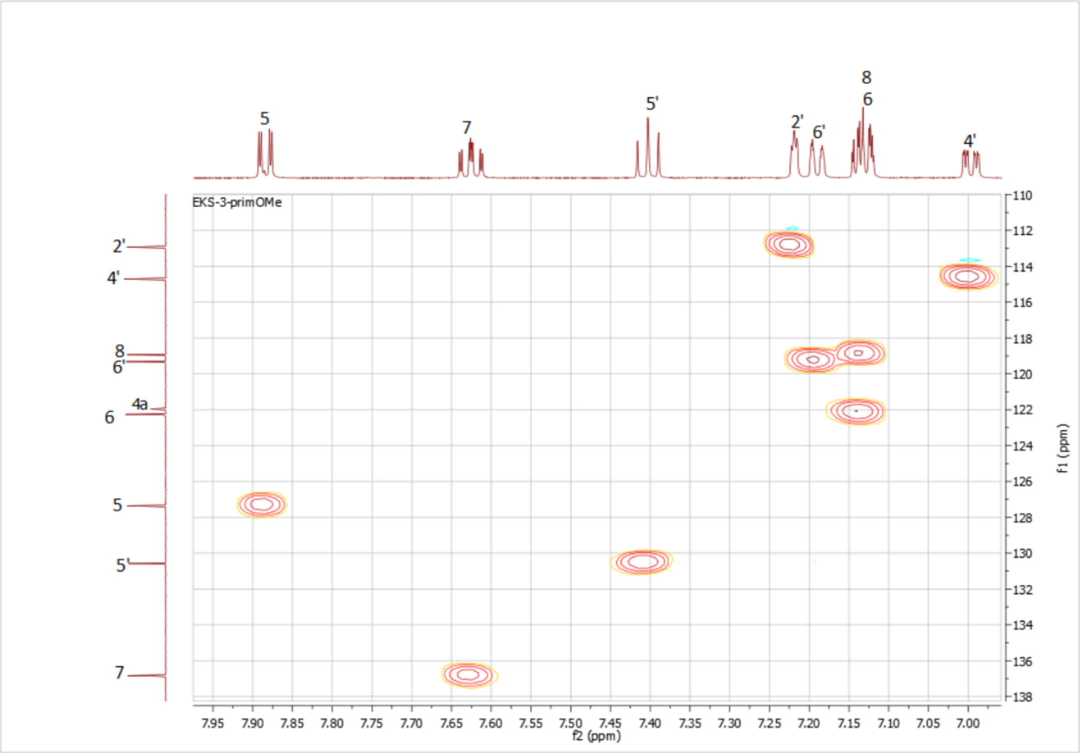

Figure S28. HSQC NMR spectrum of 3′-methoxyflavanone (2) (Acetone-d$_6$, 151 MHz)

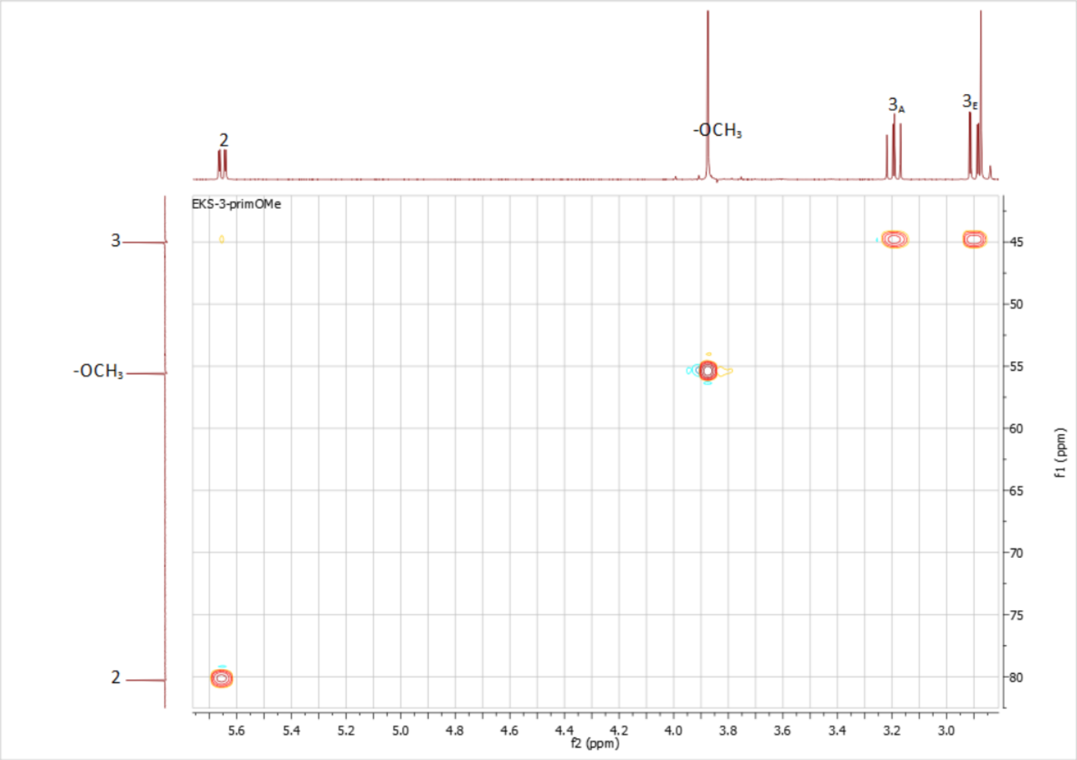

Figure S29. HSQC NMR spectrum of 3′-methoxyflavanone (2) (Acetone-d_6, 151 MHz)

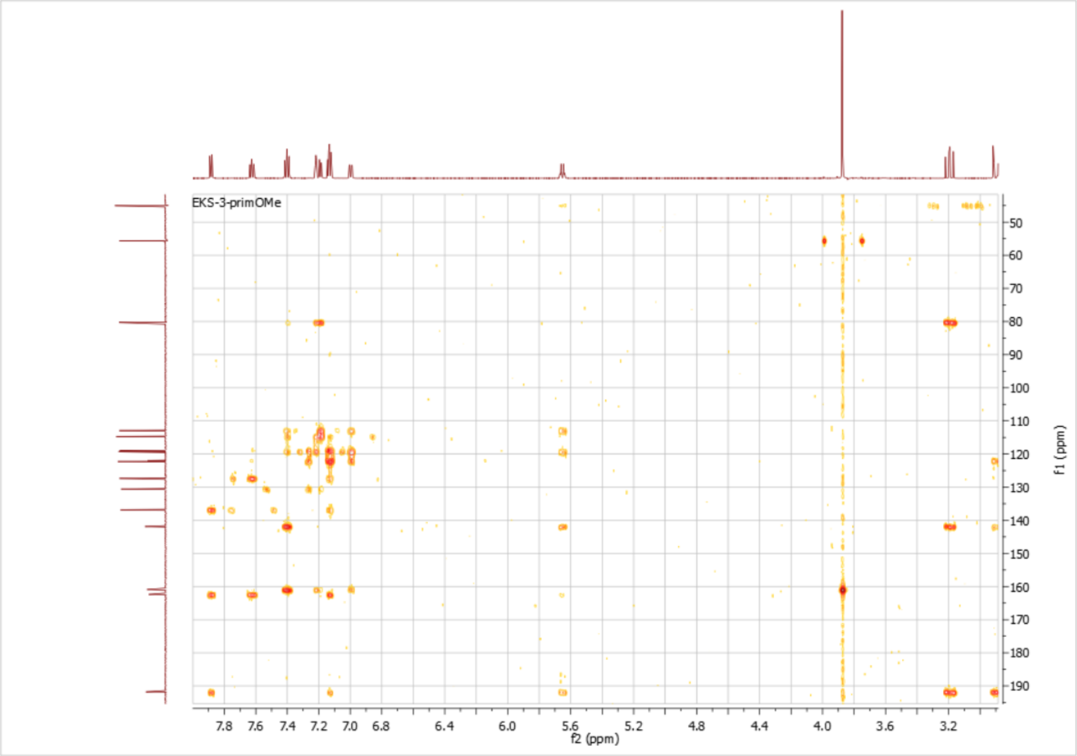

Figure S30. HMBC NMR spectrum of 3′-methoxyflavanone (2) (Acetone-d_6, 151 MHz)

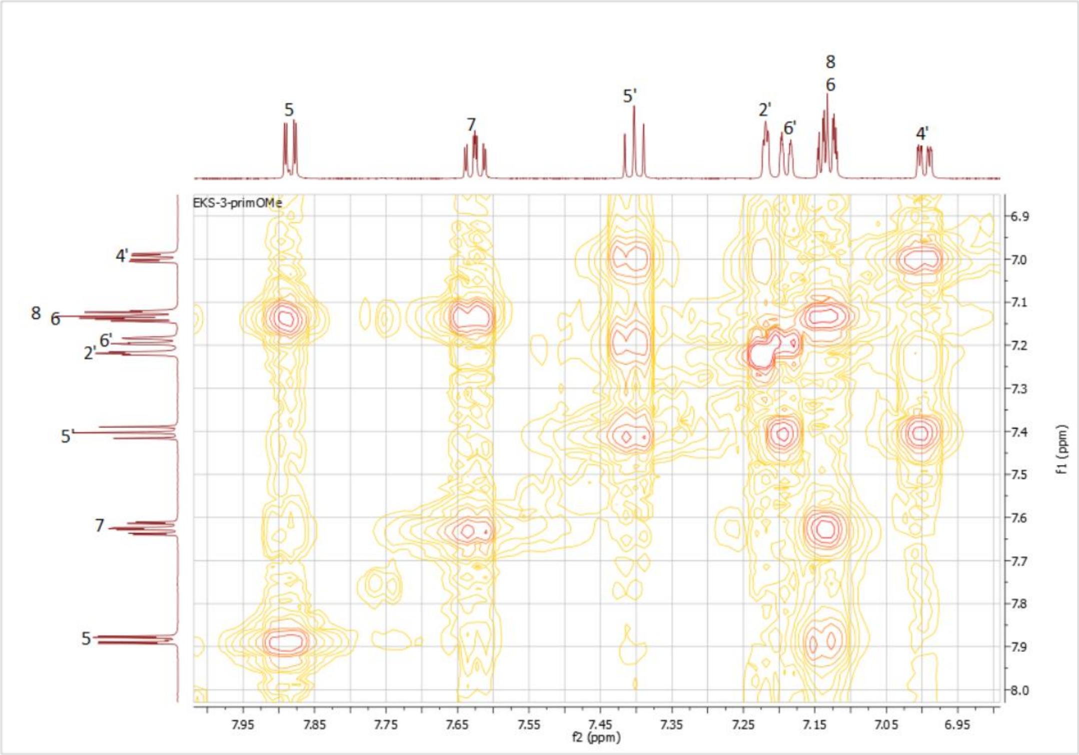

Figure S31. COSY NMR spectrum of 3′-methoxyflavanone (2) (Acetone-d6, 600 MHz)

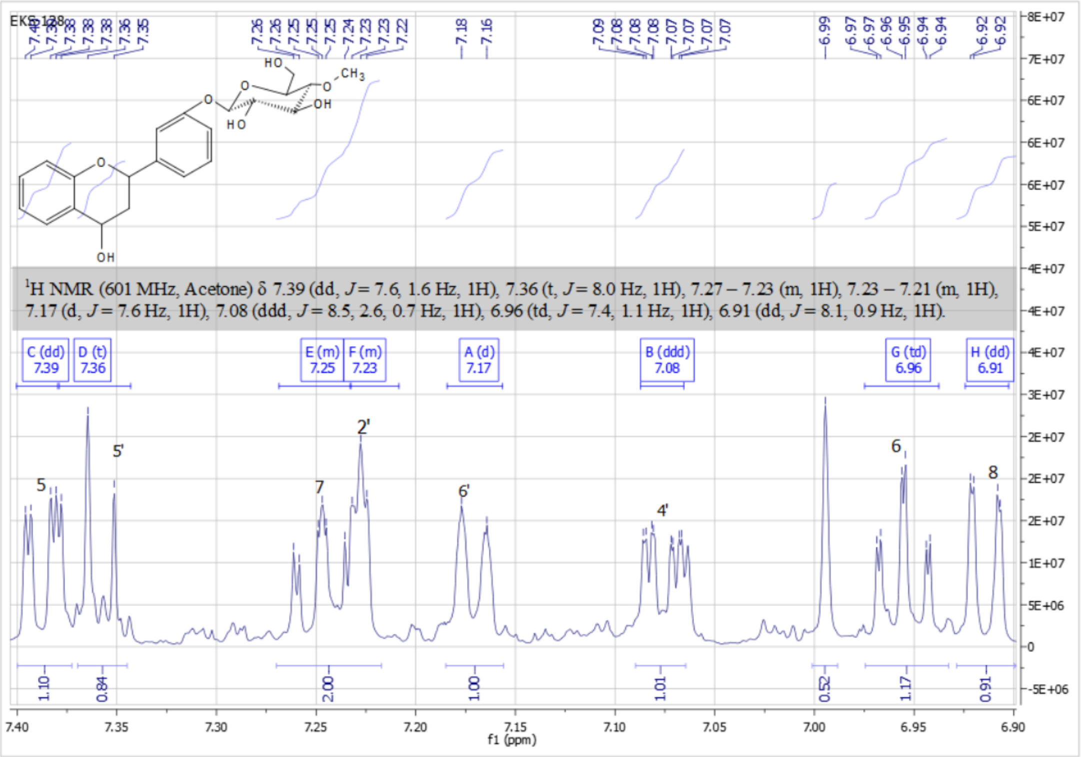

Figure S32. ^{1}H NMR spectrum of flavan-4-ol 3′-*O*-β-D-(4′′-*O*-methyl)-glucopyranoside (2a) (Acetone-d6, 600 MHz)

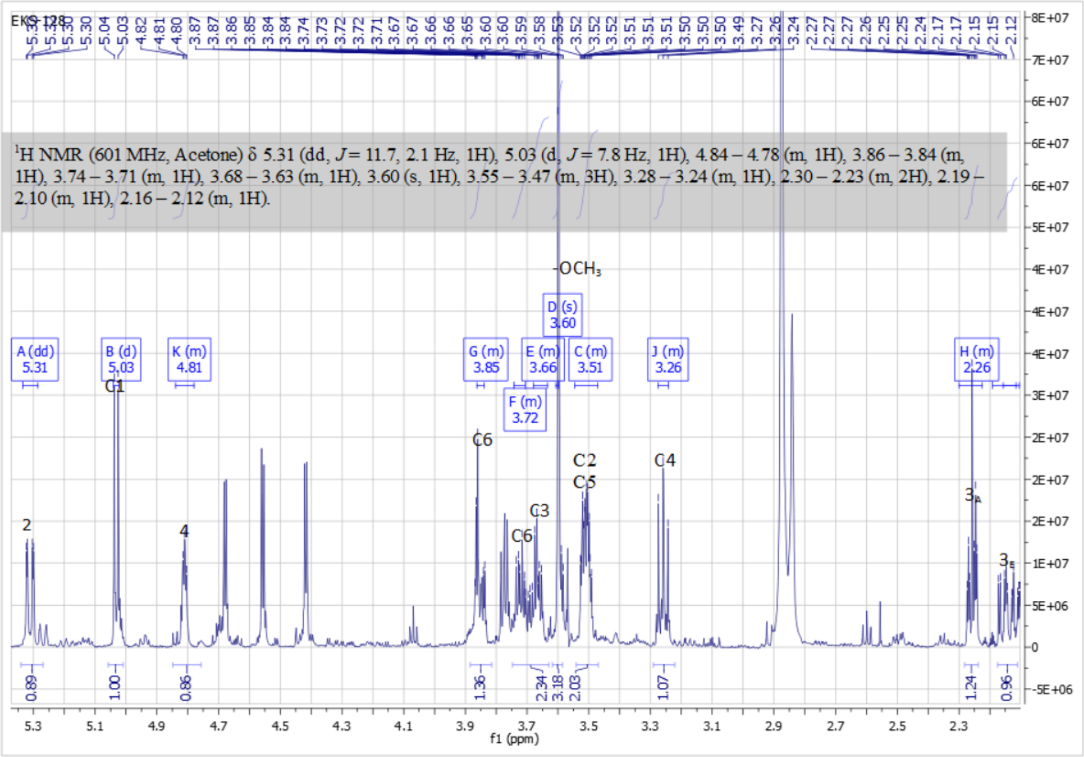

Figure S33. ¹H NMR spectrum of flavan-4-ol 3′-*O*-β-D-(4″-*O*-methyl)-glucopyranoside (2a) (Acetone-d₆, 600 MHz)

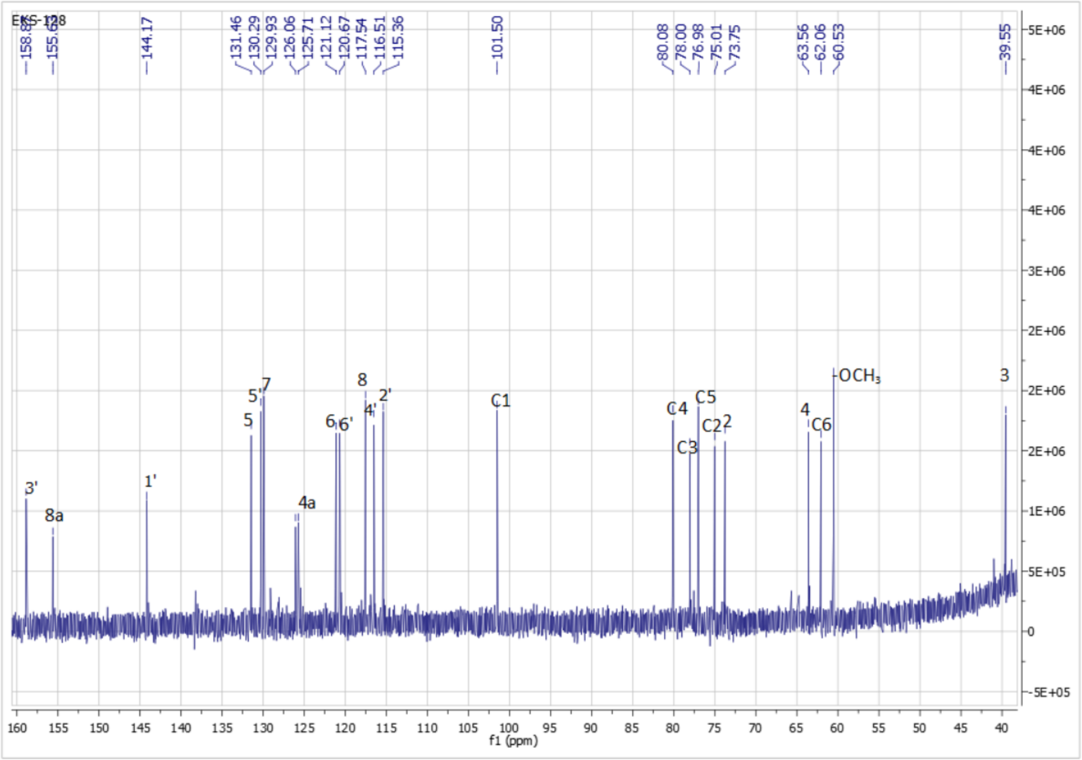

Figure S34. ¹³C NMR spectrum of flavan-4-ol 3′-*O*-β-D-(4″-*O*-methyl)-glucopyranoside (2a) (Acetone-d₆, 151 MHz)

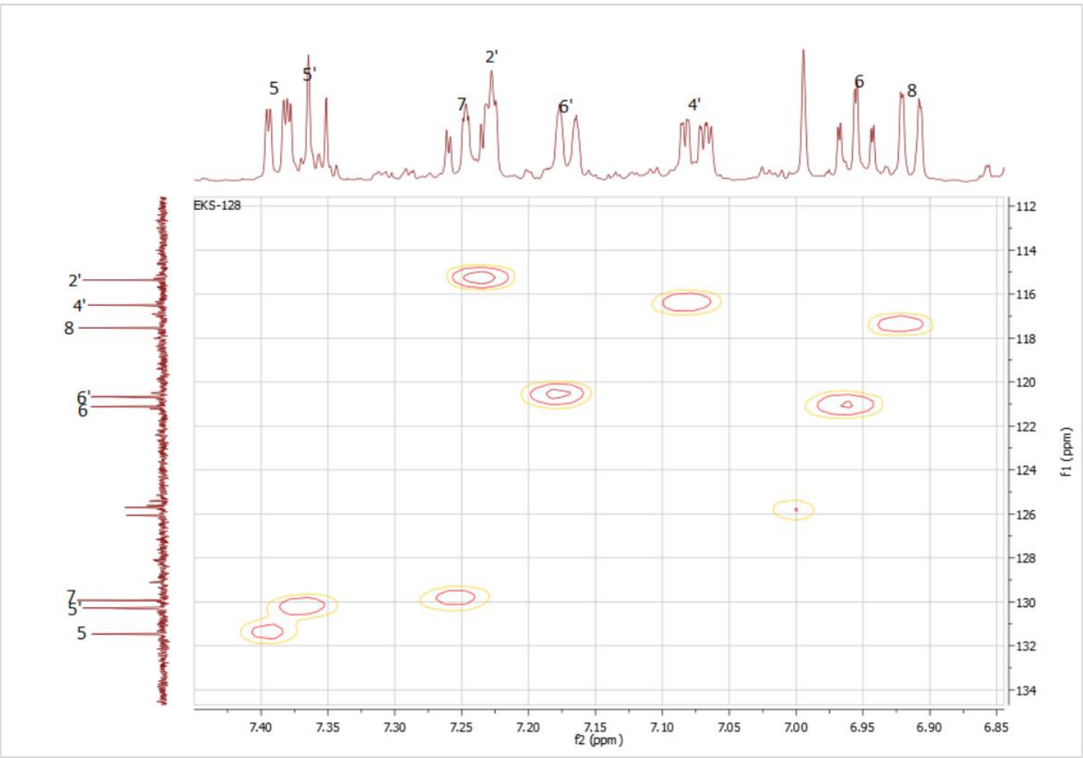

Figure S35. HSQC NMR spectrum of flavan-4-ol 3′-*O*-β-D-(4′′-*O*-methyl)-glucopyranoside (2a) (Acetone-d6, 151 MHz)

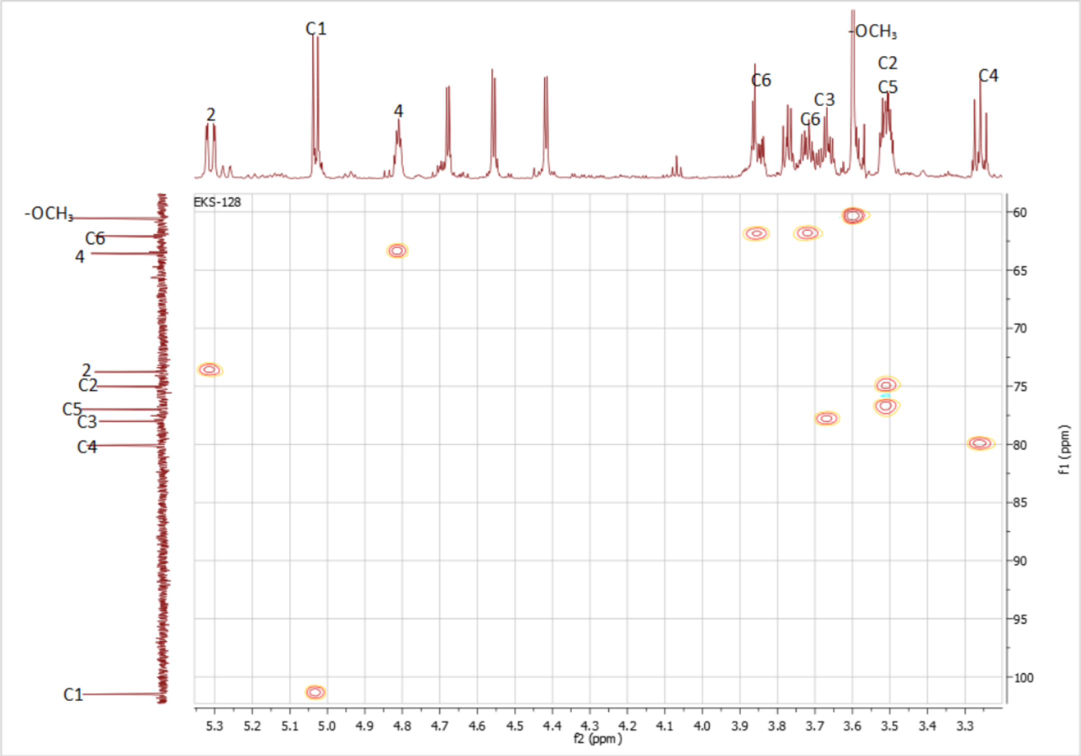

Figure S36. HSQC NMR spectrum of flavan-4-ol 3′-*O*-β-D-(4′′-*O*-methyl)-glucopyranoside (2a) (Acetone-d6, 151 MHz)

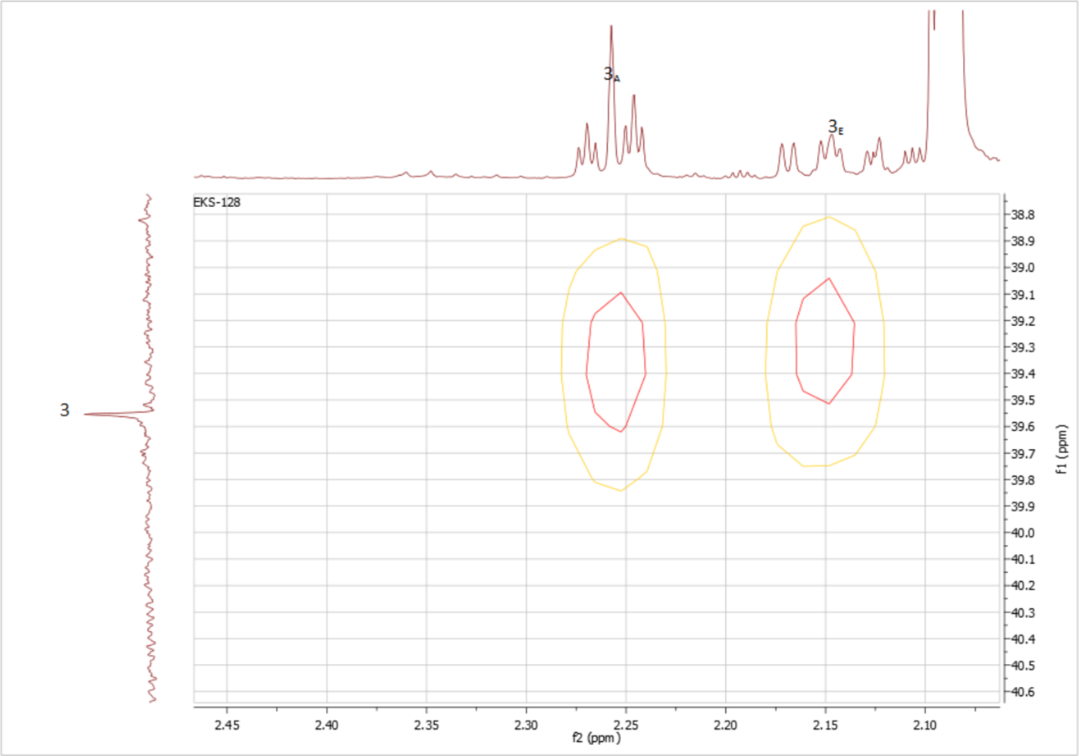

Figure S37. HSQC NMR spectrum of flavan-4-ol 3′-*O*-β-D-(4″-*O*-methyl)-glucopyranoside (2a) (Acetone-d_6, 151 MHz)

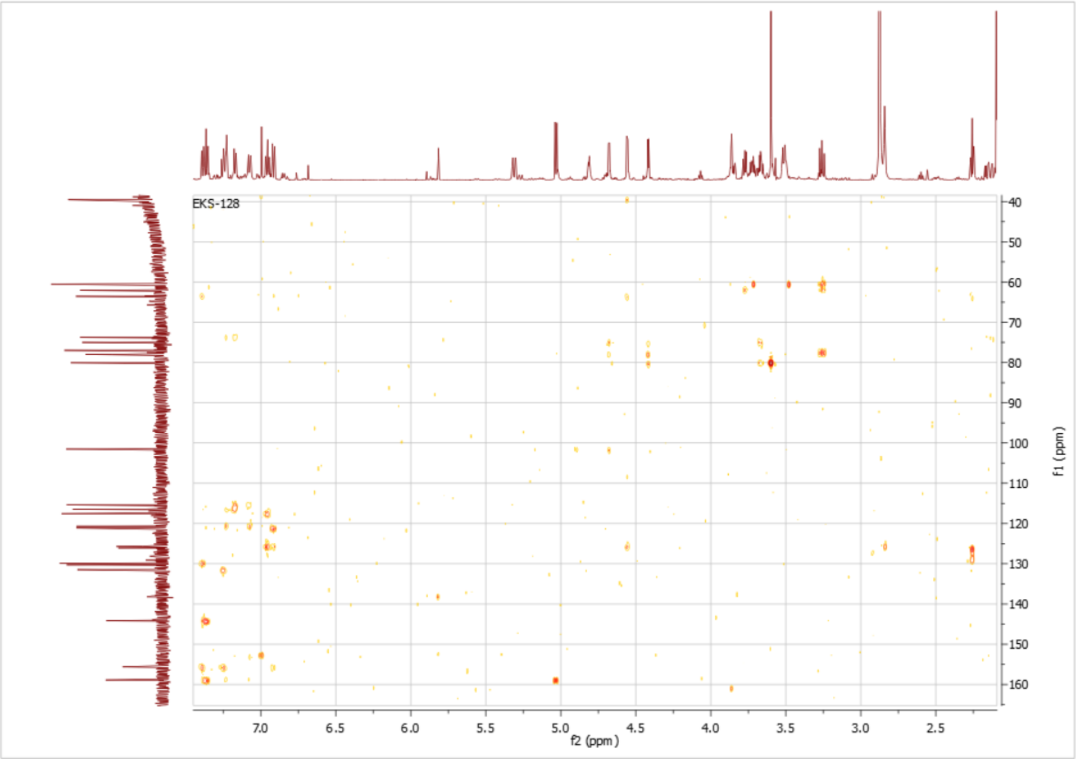

Figure S38. HMBC NMR spectrum of flavan-4-ol 3′-*O*-β-D-(4″-*O*-methyl)-glucopyranoside (2a) (Acetone-d_6, 151 MHz)

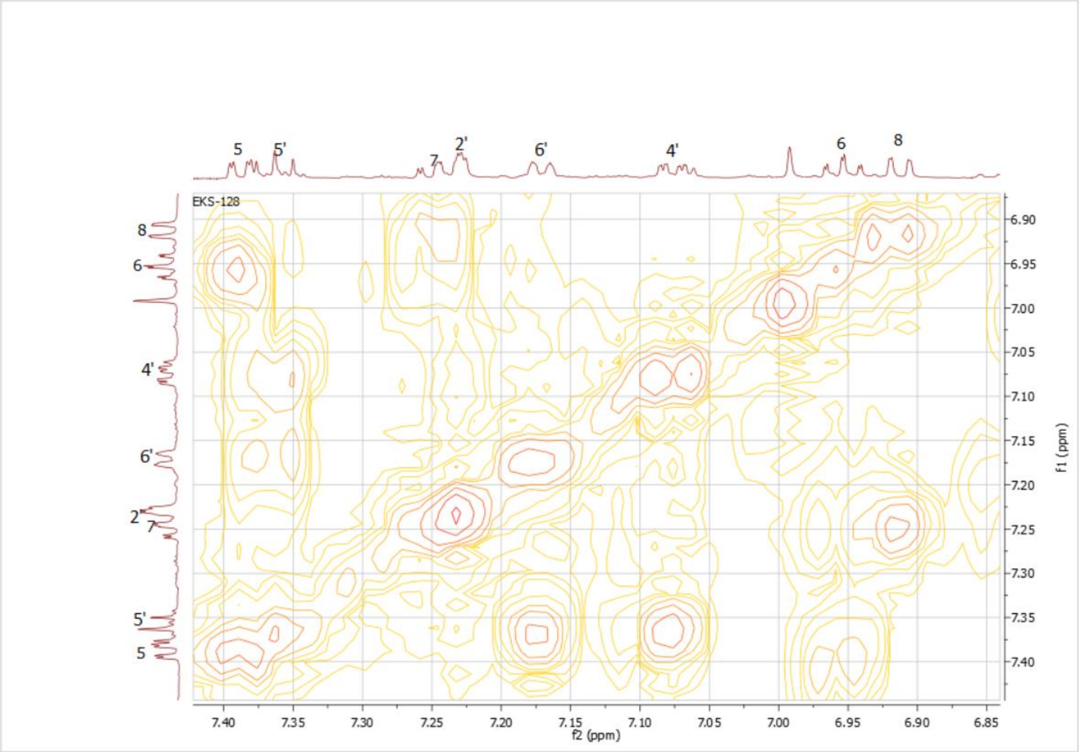

Figure S39. COSY NMR spectrum of flavan-4-ol 3′-*O*-β-D-(4″-*O*-methyl)-glucopyranoside (2a) (Acetone-d_6, 600 MHz)

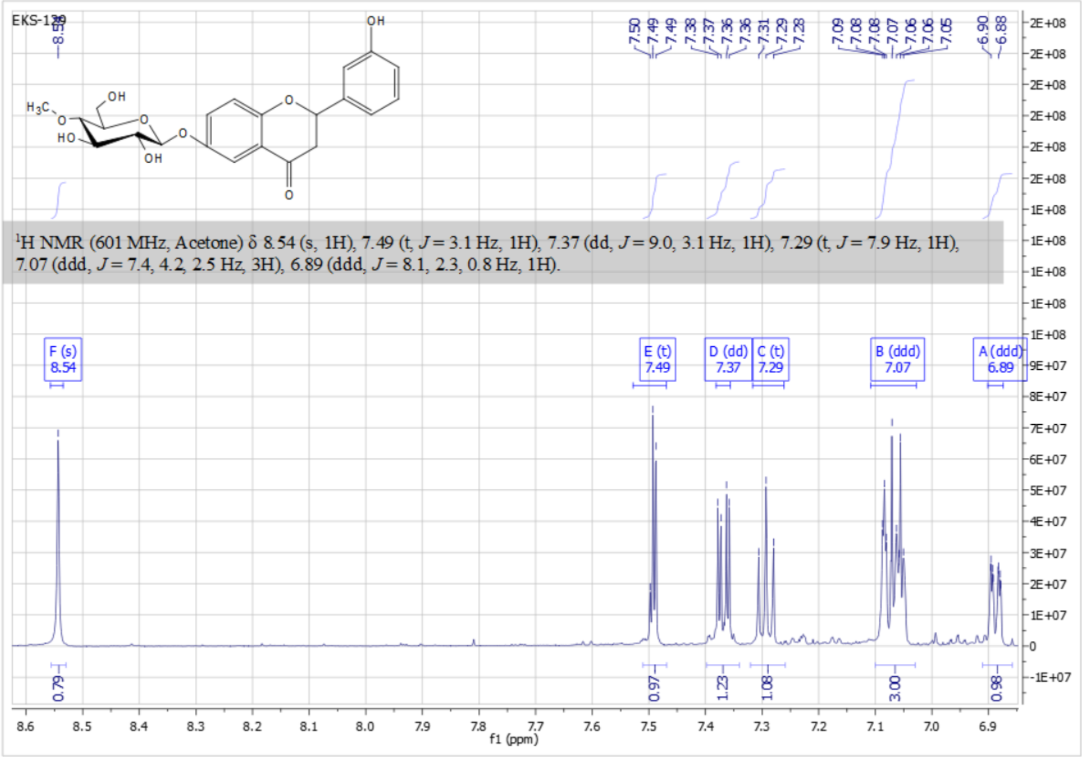

Figure S40. ^{1}H NMR spectrum of 3′-hydroxyflavanone 6-*O*-β-D-(4″-*O*-methyl)-glucopyranoside (2b) (Acetone-d_6, 600 MHz)

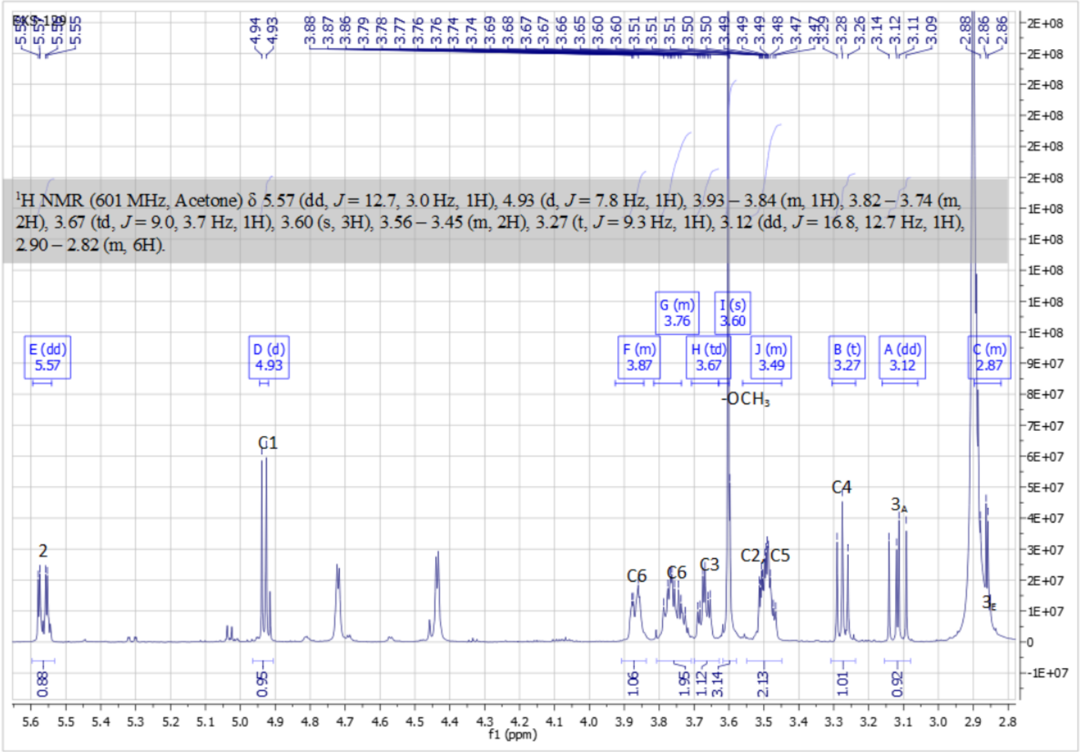

Figure S41. ¹H NMR spectrum of 3′-hydroxyflavanone 6-*O*-β-D-(4″-*O*-methyl)-glucopyranoside (2b) (Acetone-d₆, 600 MHz)

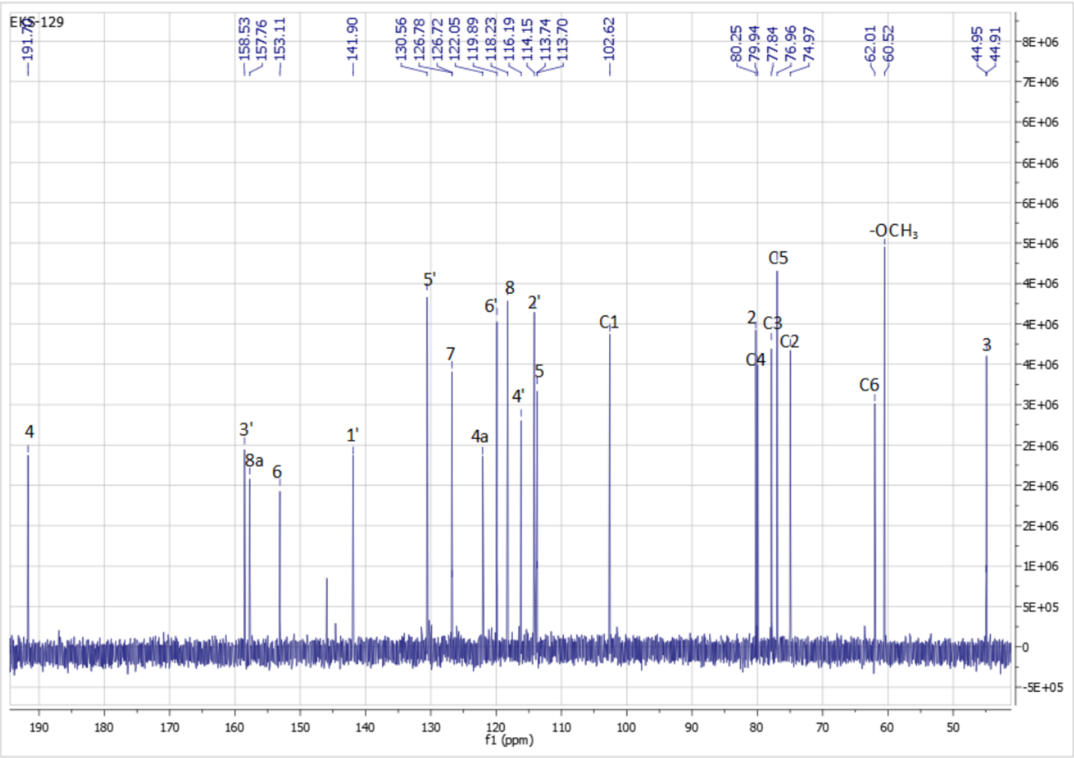

Figure S42. ¹³C NMR spectrum of 3′-hydroxyflavanone 6-*O*-β-D-(4″-*O*-methyl)-glucopyranoside (2b) (Acetone-d₆, 151 MHz)

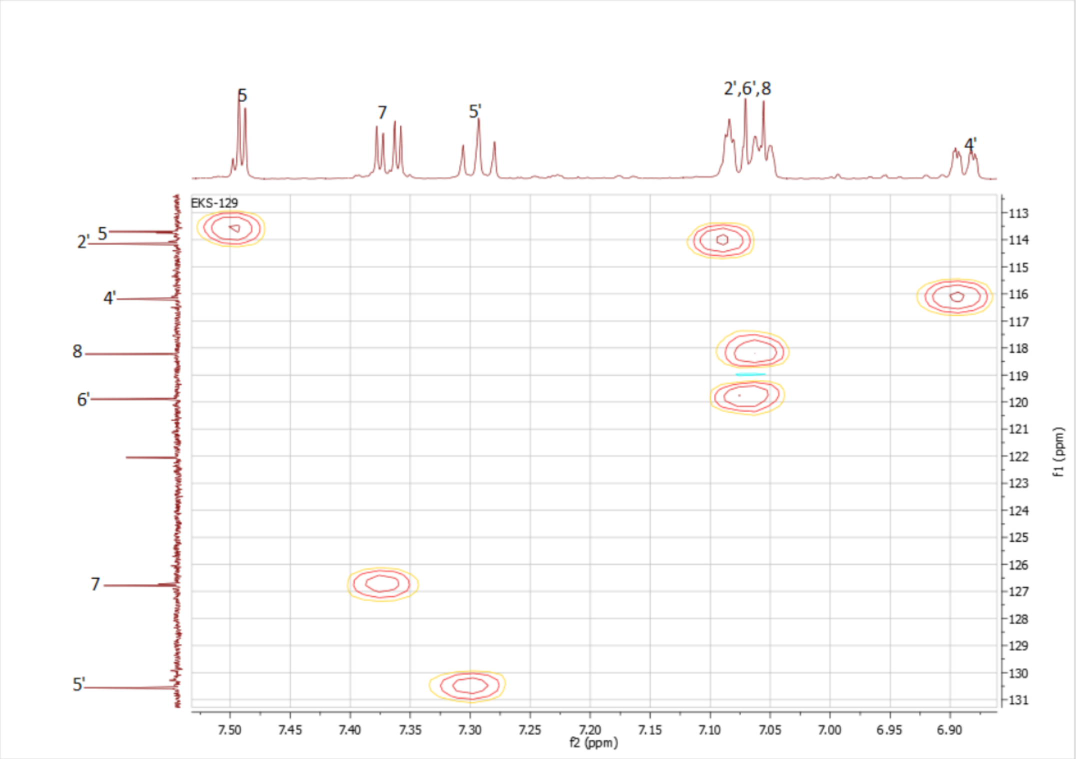

Figure S43. HSQC NMR spectrum of 3′-hydroxyflavanone 6-*O*-β-D-(4″-*O*-methyl)-glucopyranoside (2b) (Acetone-d$_6$, 151 MHz)

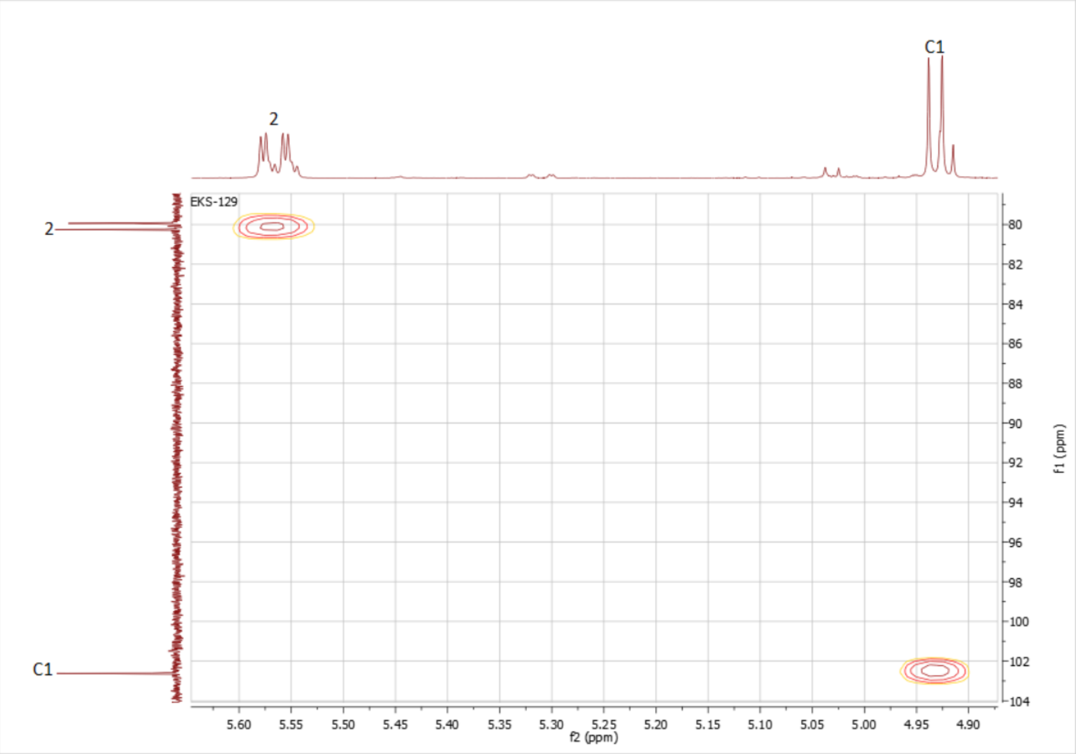

Figure S44. HSQC NMR spectrum of 3′-hydroxyflavanone 6-*O*-β-D-(4″-*O*-methyl)-glucopyranoside (2b) (Acetone-d$_6$, 151 MHz)

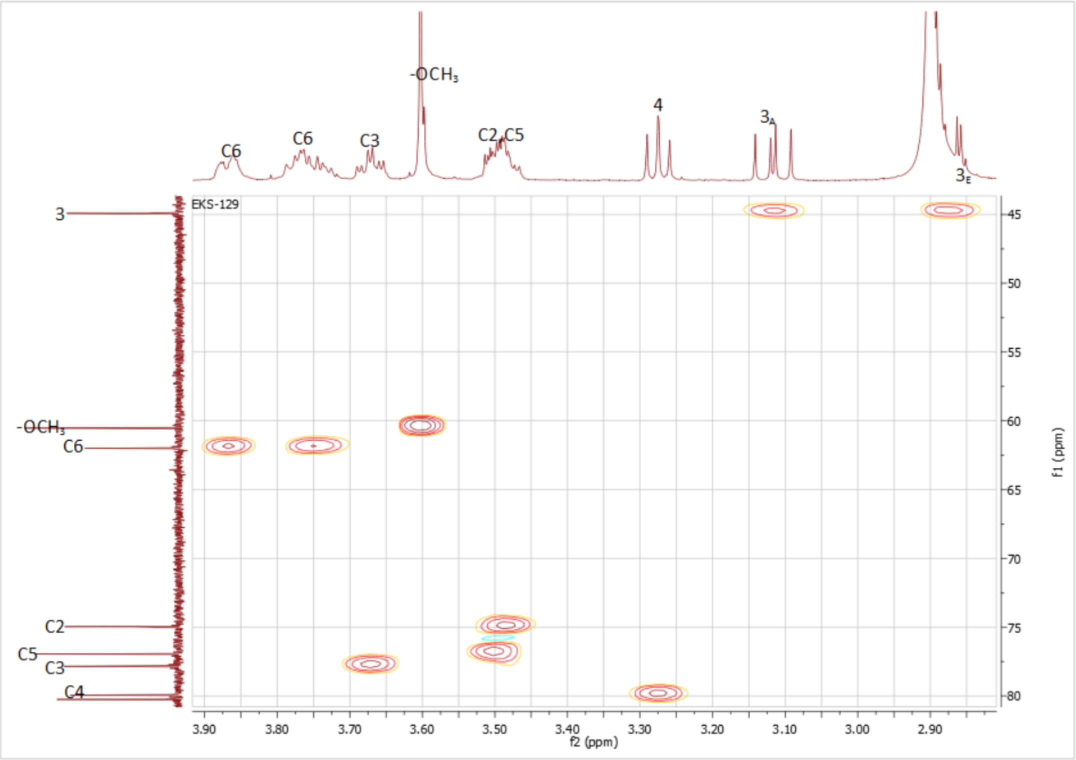

Figure S45. HSQC NMR spectrum of 3′-hydroxyflavanone 6-*O*-β-D-(4″-*O*-methyl)-glucopyranoside (2b) (Acetone-d6, 151 MHz)

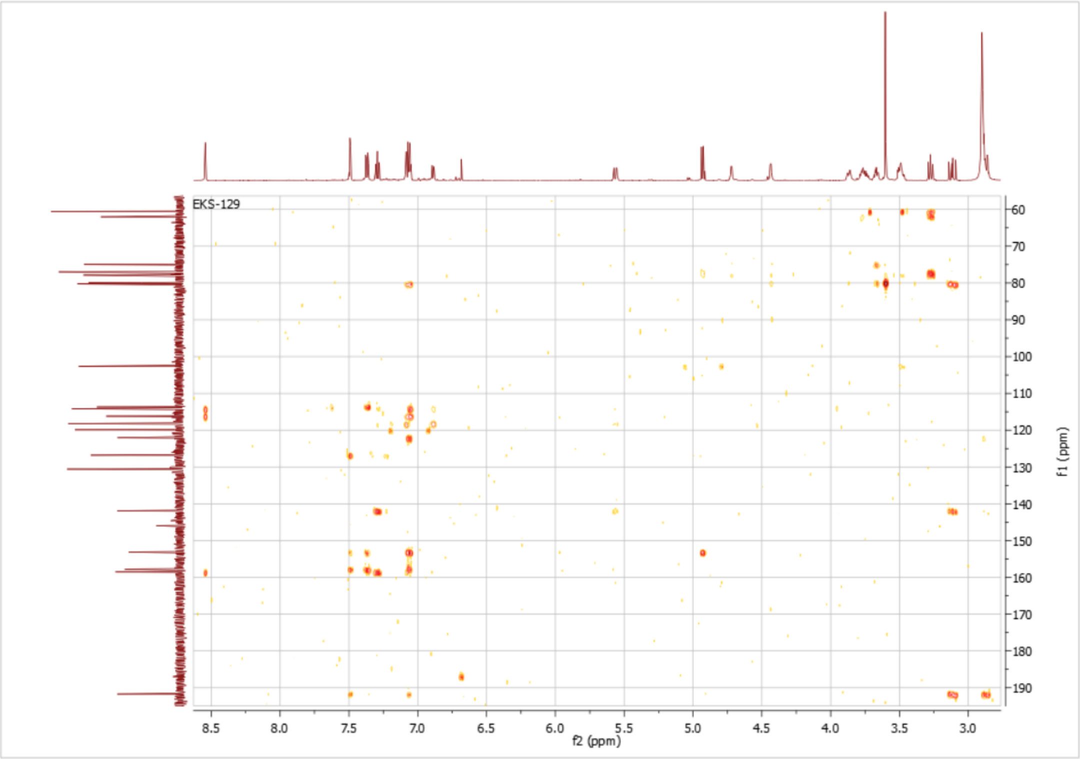

Figure S46. HMBC NMR spectrum of 3′-hydroxyflavanone 6-*O*-β-D-(4″-*O*-methyl)-glucopyranoside (2b) (Acetone-d6, 151 MHz)

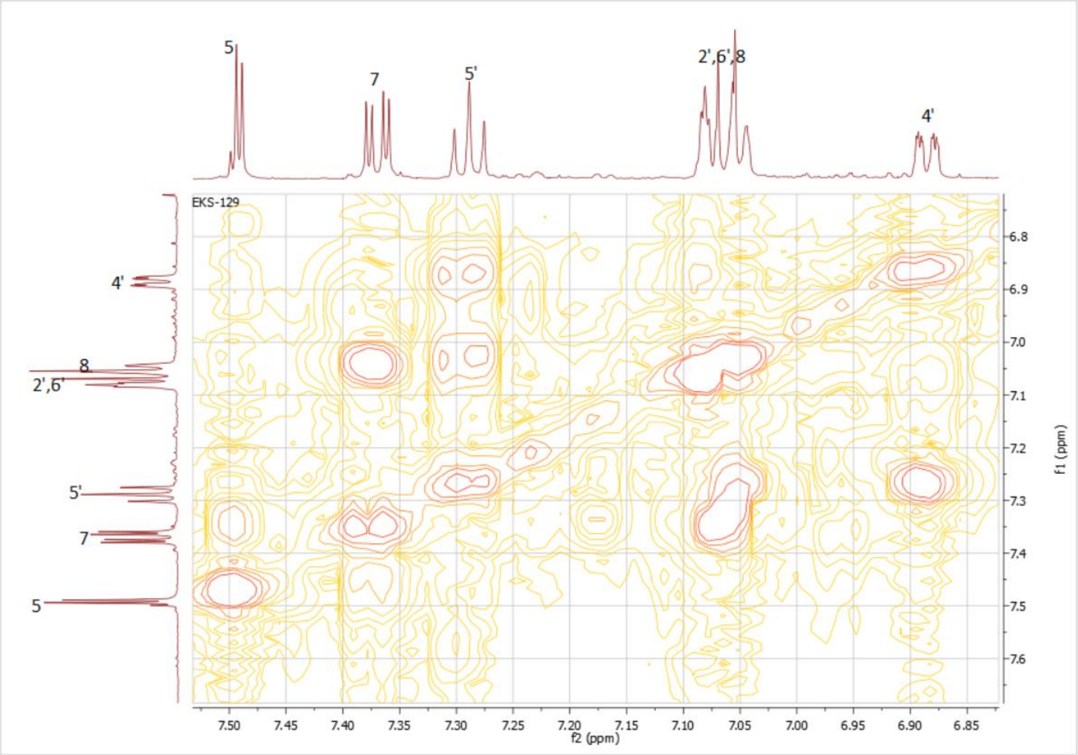

Figure S47. COSY NMR spectrum of 3′-hydroxyflavanone 6-*O*-β-D-(4′′-*O*-methyl)-glucopyranoside (2b) (Acetone-d$_6$, 600 MHz)

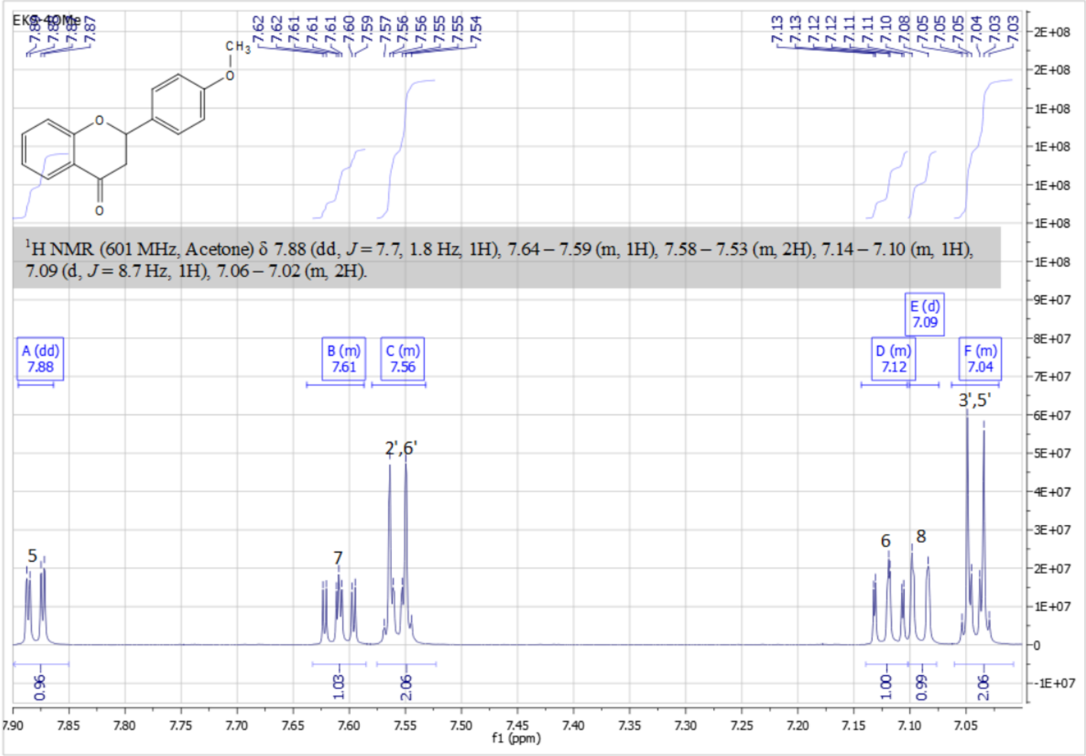

Figure S48. ^{1}H NMR spectrum of 4′-methoxyflavanone (3) (Acetone-d$_6$, 600 MHz)

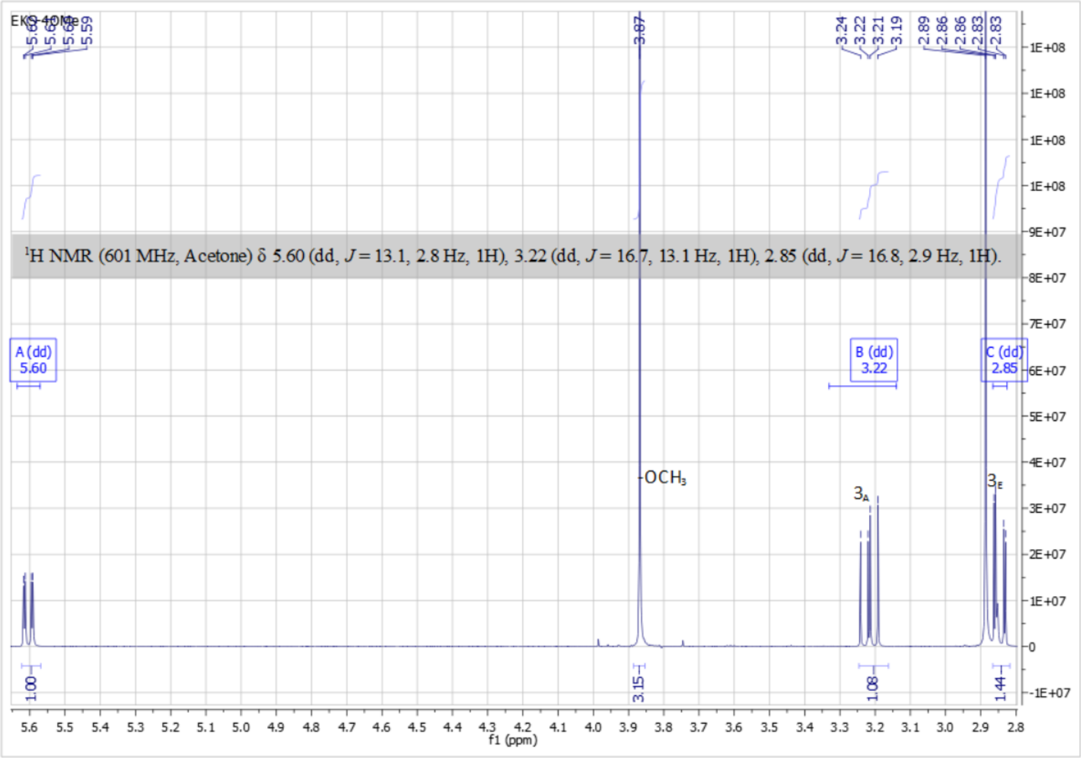

Figure S49. ^{1}H NMR spectrum of 4′-methoxyflavanone (3) (Acetone-d_6, 600 MHz)

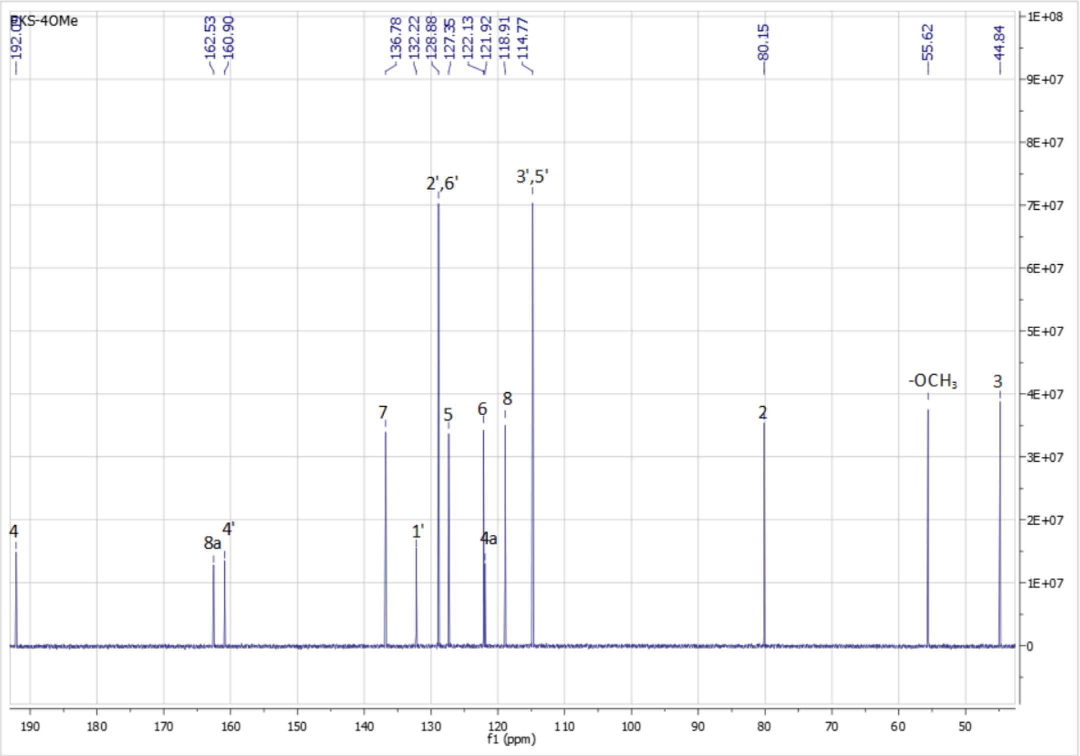

Figure S50. ^{13}C NMR spectrum of 4′-methoxyflavanone (3) (Acetone-d_6, 151 MHz)

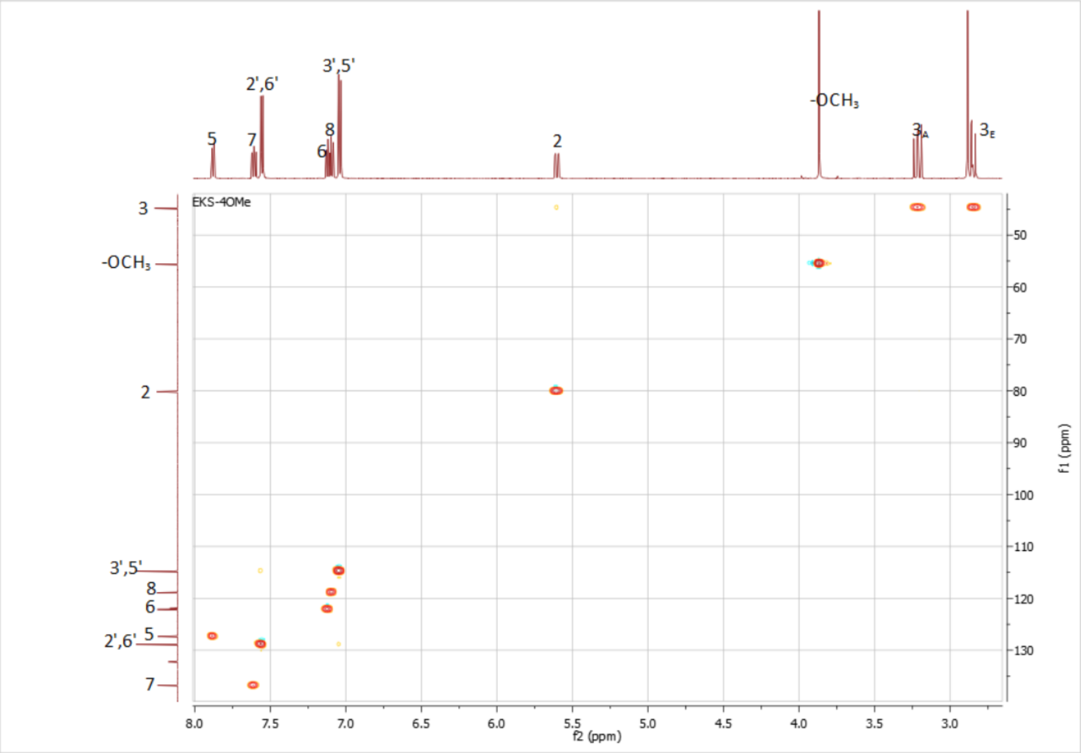

Figure S51. HSQC NMR spectrum of 4′-methoxyflavanone (3) (Acetone-d6, 151 MHz)

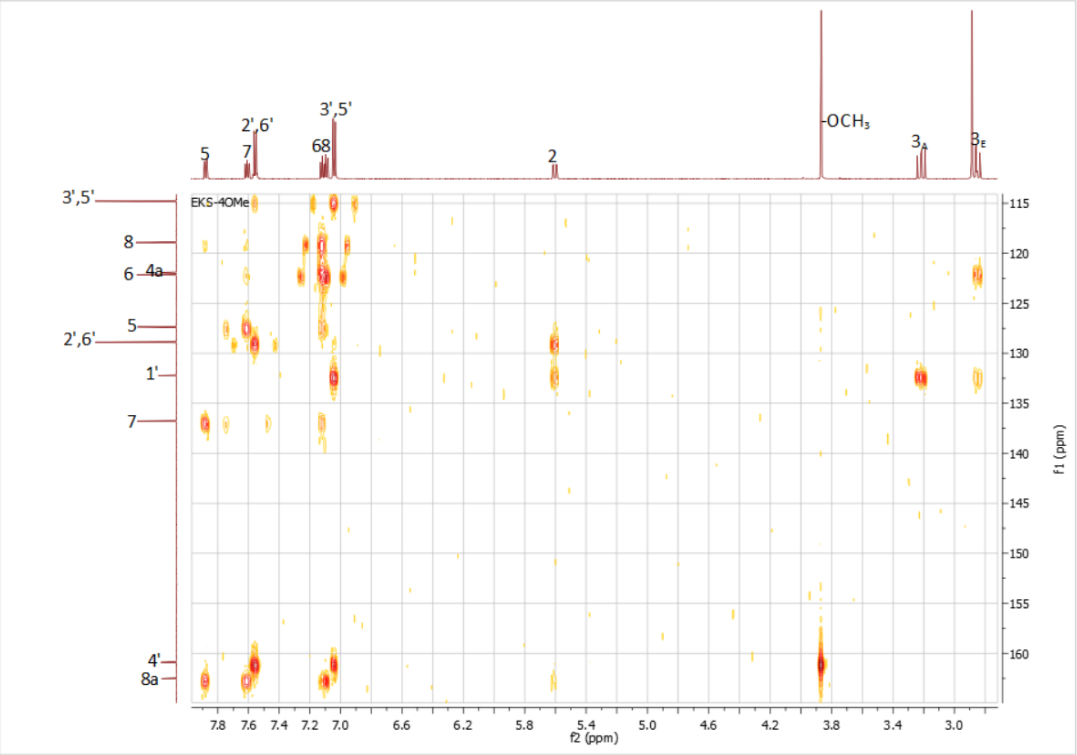

Figure S52. HMBC NMR spectrum of 4′-methoxyflavanone (3) (Acetone-d6, 151 MHz)

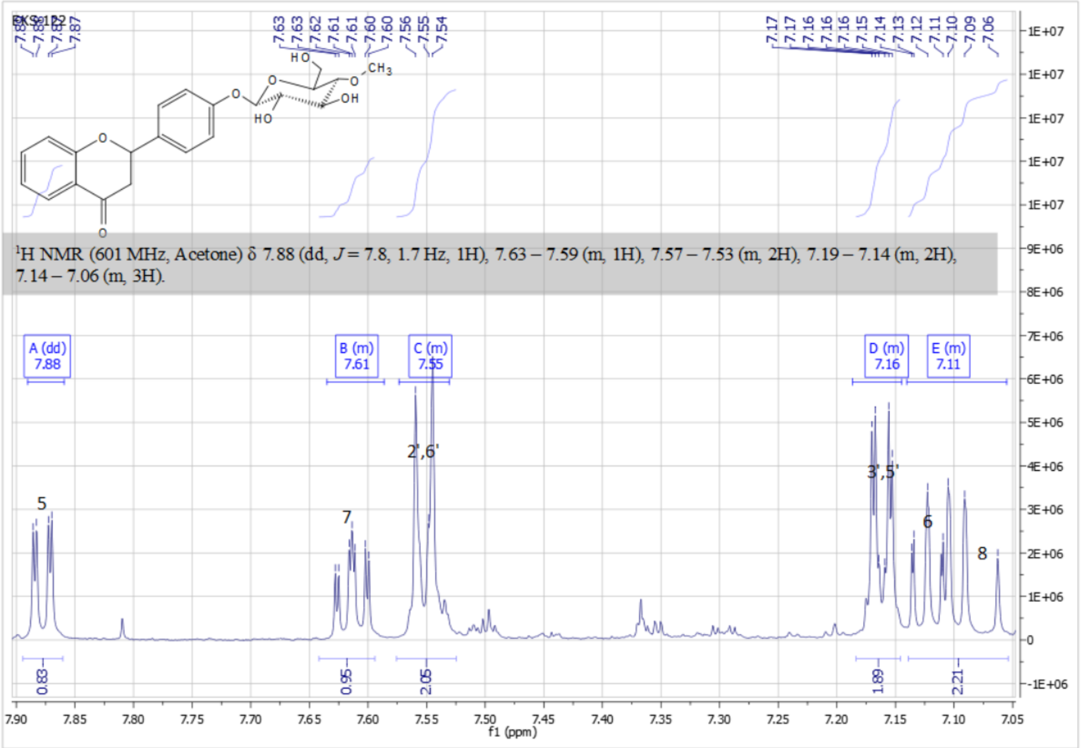

Figure S53. ¹H NMR spectrum of flavanone 4′-*O*-β-D-(4″-*O*-methyl)-glucopyranoside (3a) (Acetone-d₆, 600 MHz)

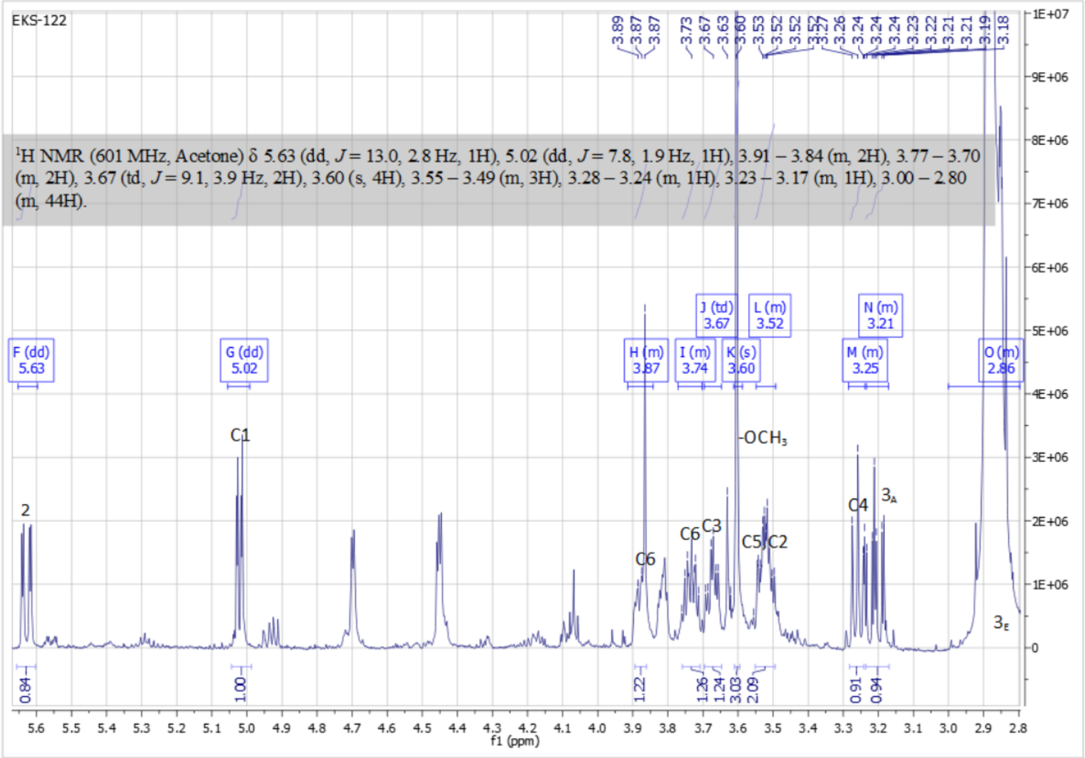

Figure S54. ¹H NMR spectrum of flavanone 4′-*O*-β-D-(4″-*O*-methyl)-glucopyranoside (3a) (Acetone-d₆, 600 MHz)

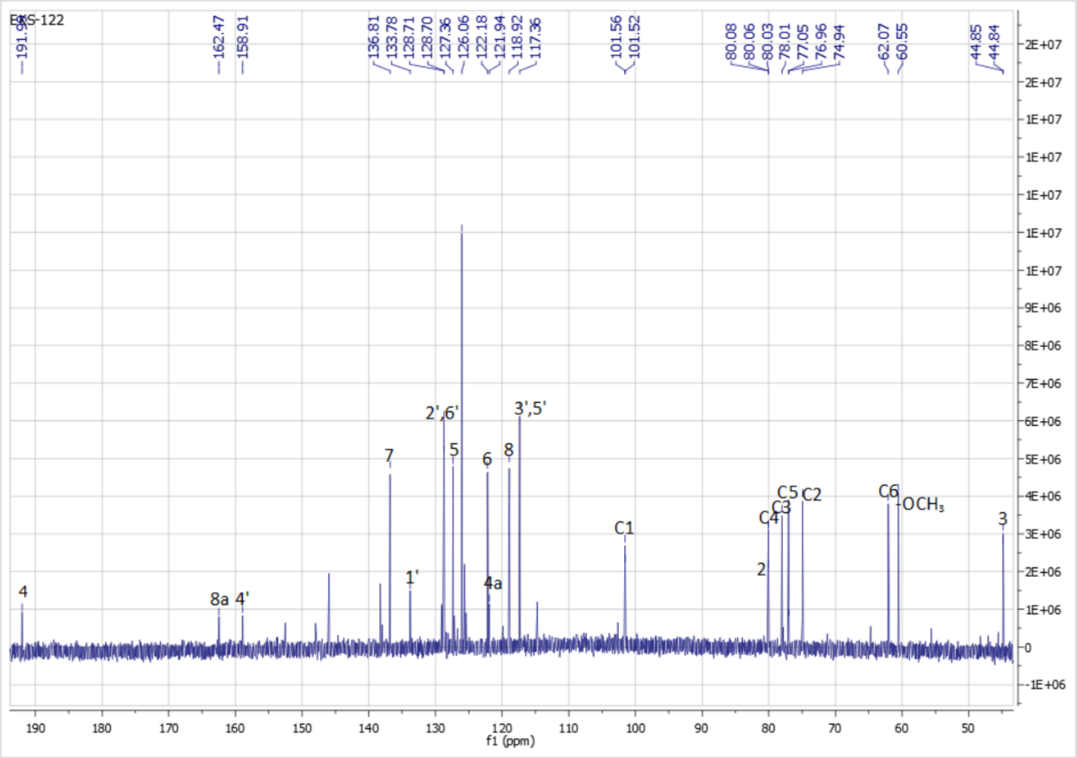

Figure S55. ^{13}C NMR spectrum of flavanone 4′-*O*-β-D-(4″-*O*-methyl)-glucopyranoside (3a) (Acetone-d$_6$, 151 MHz)

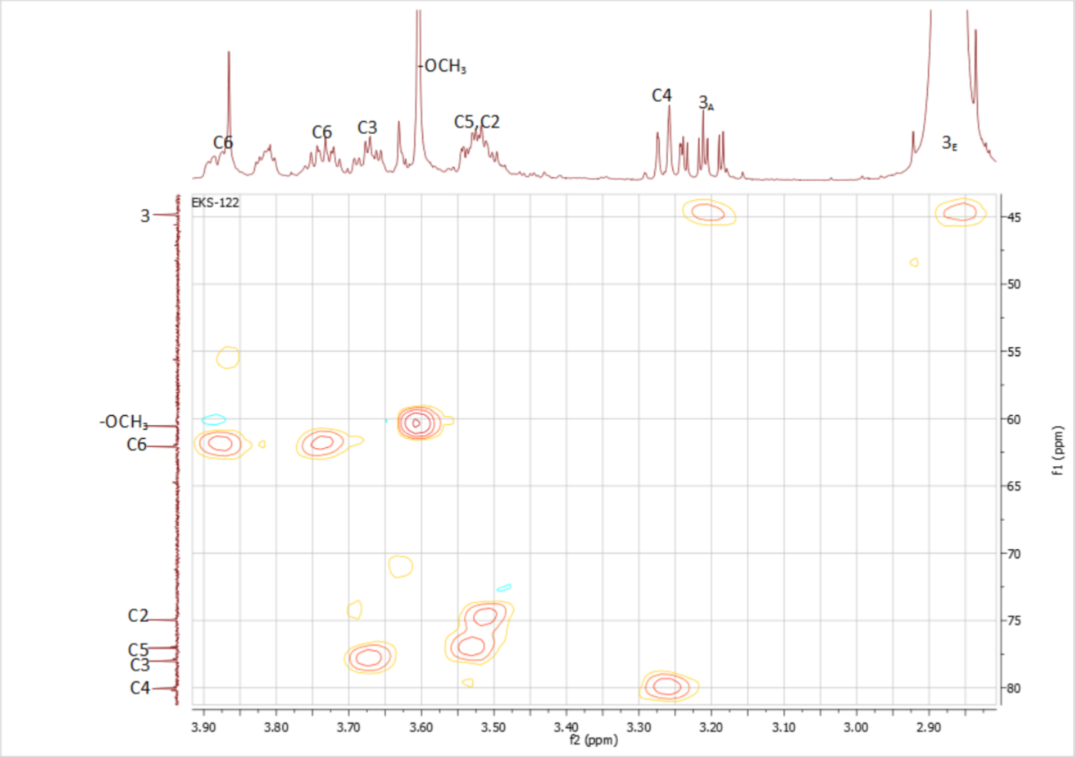

Figure S56. HSQC NMR spectrum of flavanone 4′-*O*-β-D-(4″-*O*-methyl)-glucopyranoside (3a) (Acetone-d$_6$, 151 MHz)

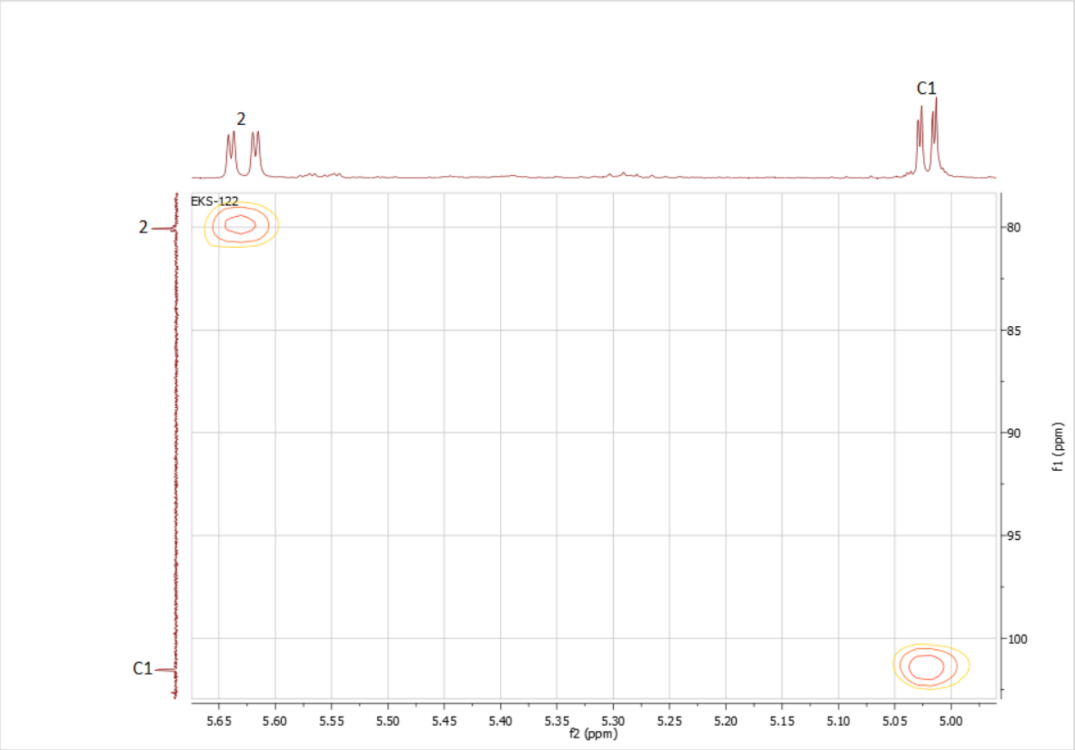

Figure S57. HSQC NMR spectrum of flavanone 4′-*O*-β-D-(4″-*O*-methyl)-glucopyranoside (3a) (Acetone-d_6, 151 MHz)

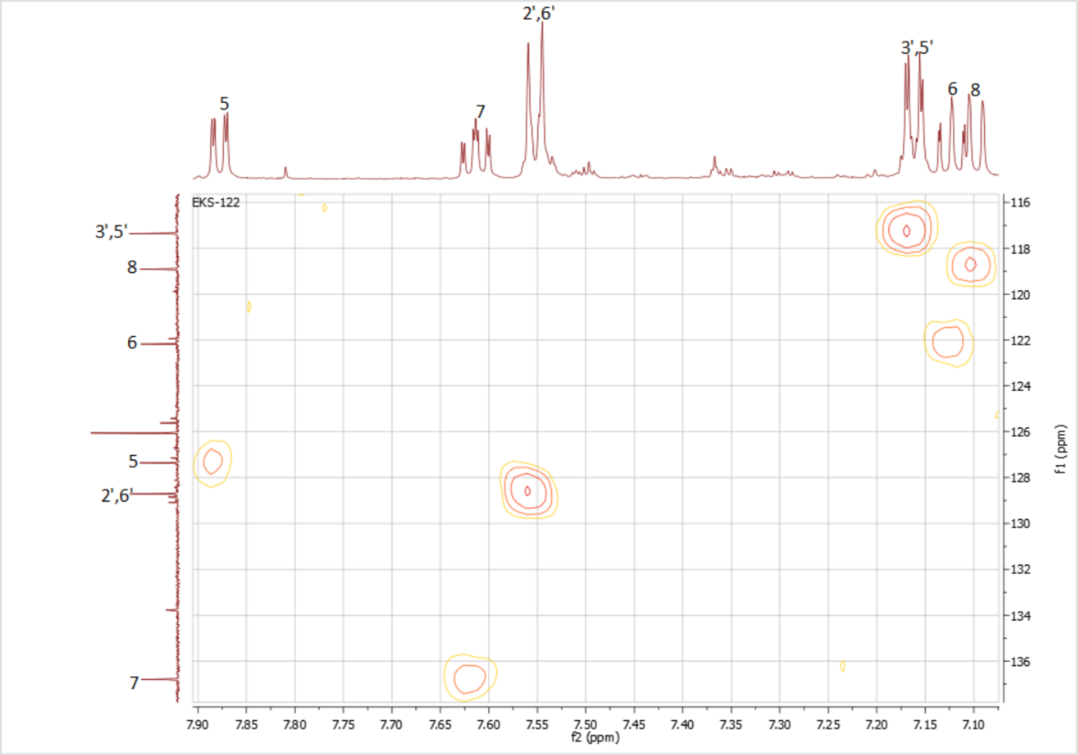

Figure S58. HSQC NMR spectrum of flavanone 4′-*O*-β-D-(4″-*O*-methyl)-glucopyranoside (3a) (Acetone-d_6, 151 MHz)

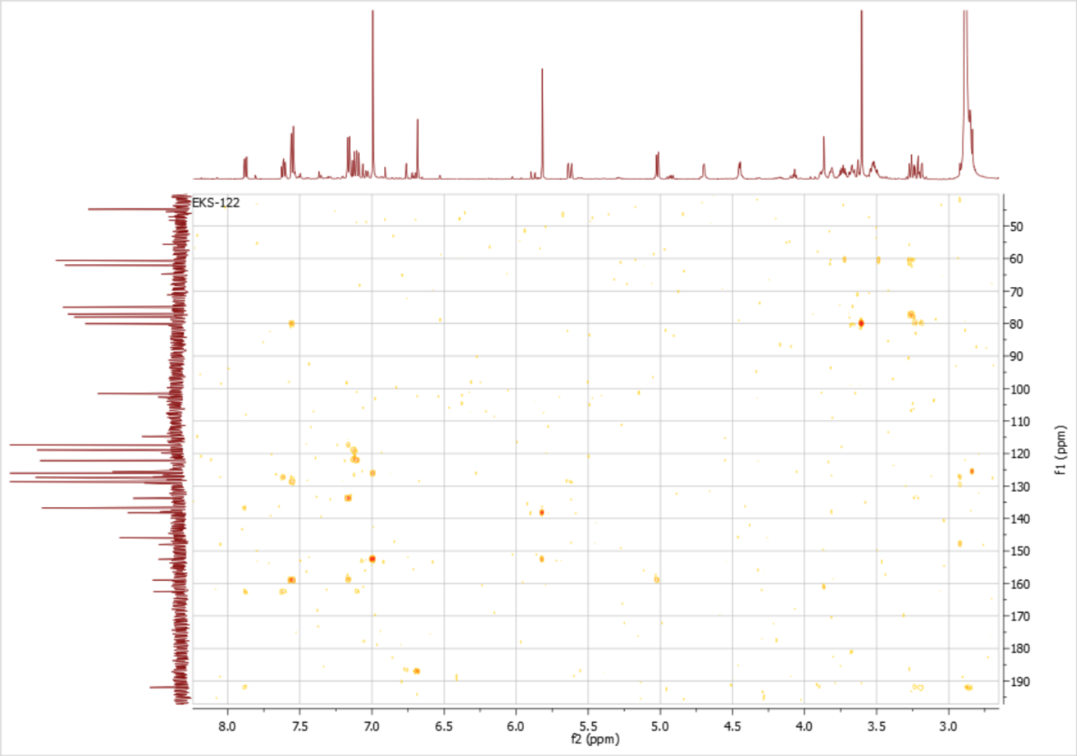

Figure S59. HMBC NMR spectrum of flavanone 4′-*O*-β-D-(4″-*O*-methyl)-glucopyranoside (3a) (Acetone-d6, 151 MHz)

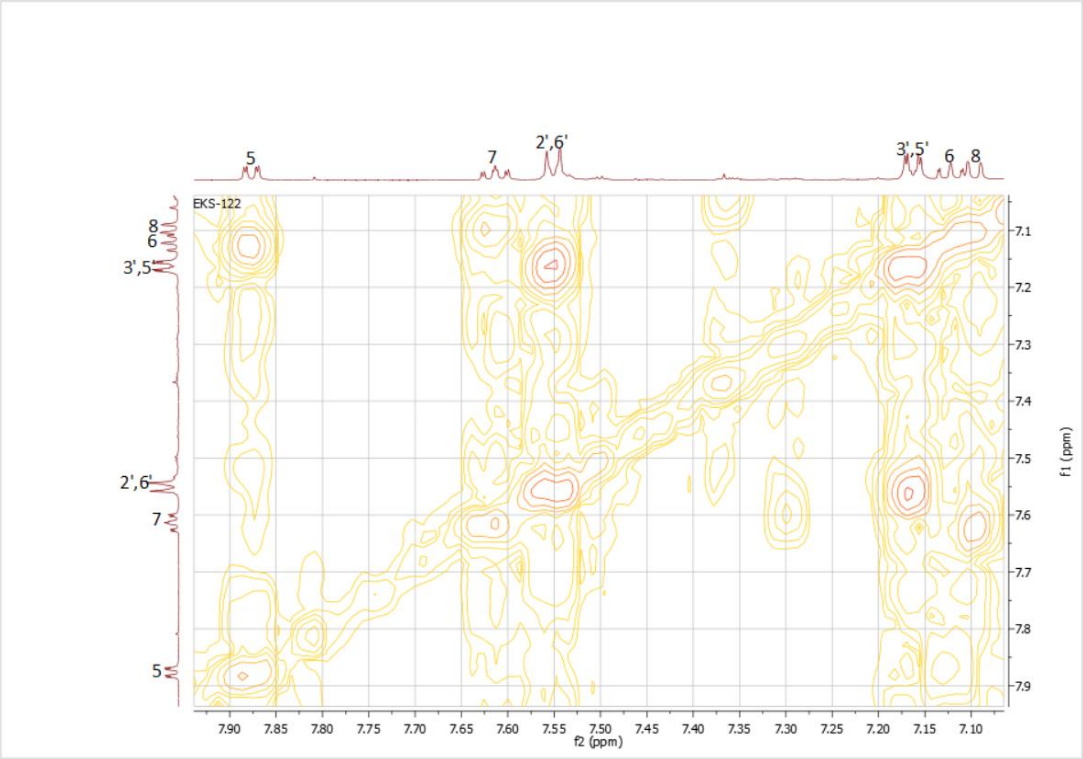

Figure S60. COSY NMR spectrum of flavanone 4′-*O*-β-D-(4″-*O*-methyl)-glucopyranoside (3a) (Acetone-d6, 600 MHz)

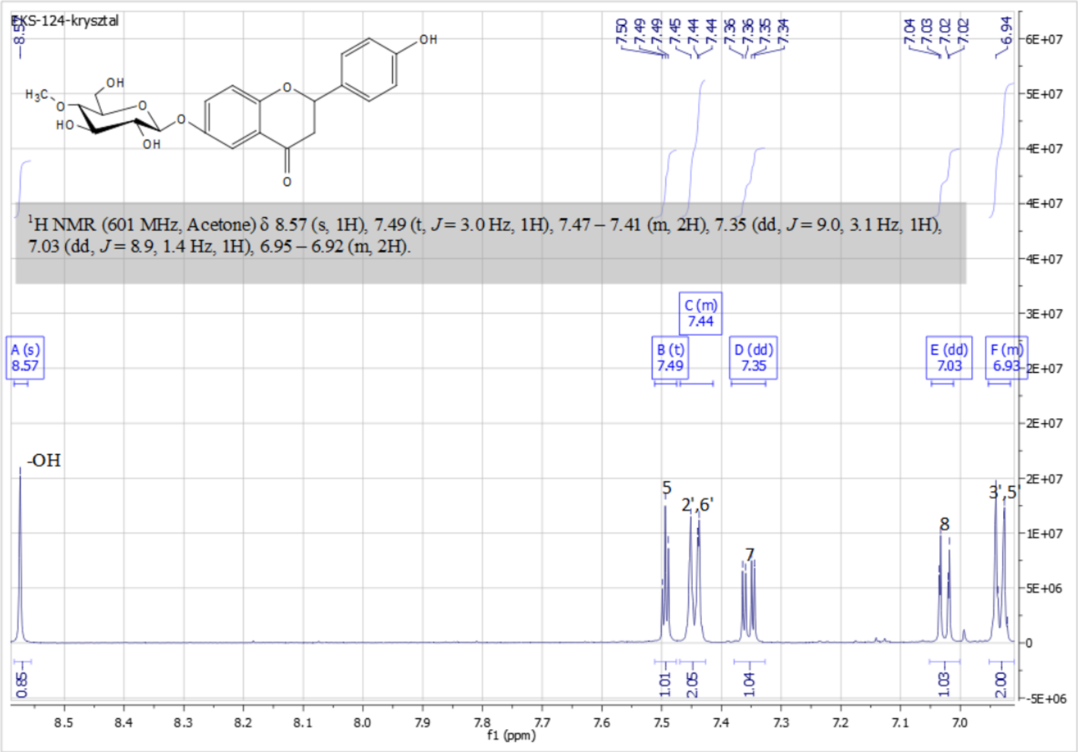

Figure S61. ¹H NMR spectrum of 4′-hydroxyflavanone 6-*O*-β-D-(4″-*O*-methyl)-glucopyranoside (3b) (Acetone-d₆, 600 MHz)

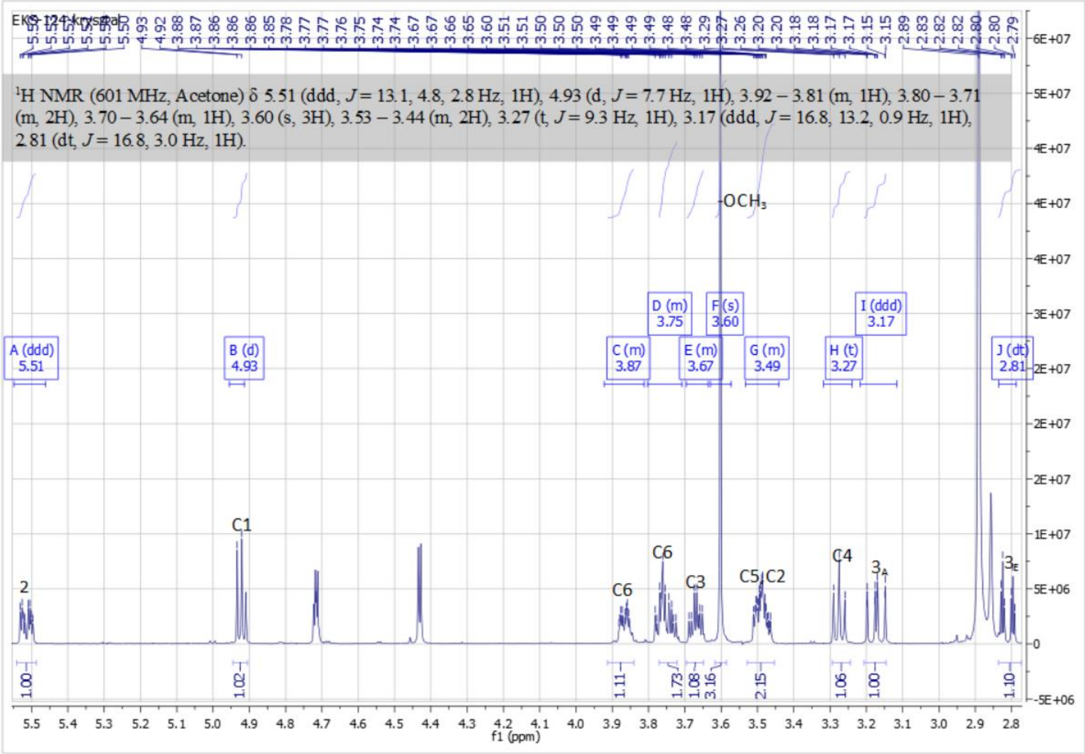

Figure S62. ¹HNMR spectrum of 4′-hydroxyflavanone 6-*O*-β-D-(4″-*O*-methyl)-glucopyranoside (3b) (Acetone-d₆, 600 MHz)

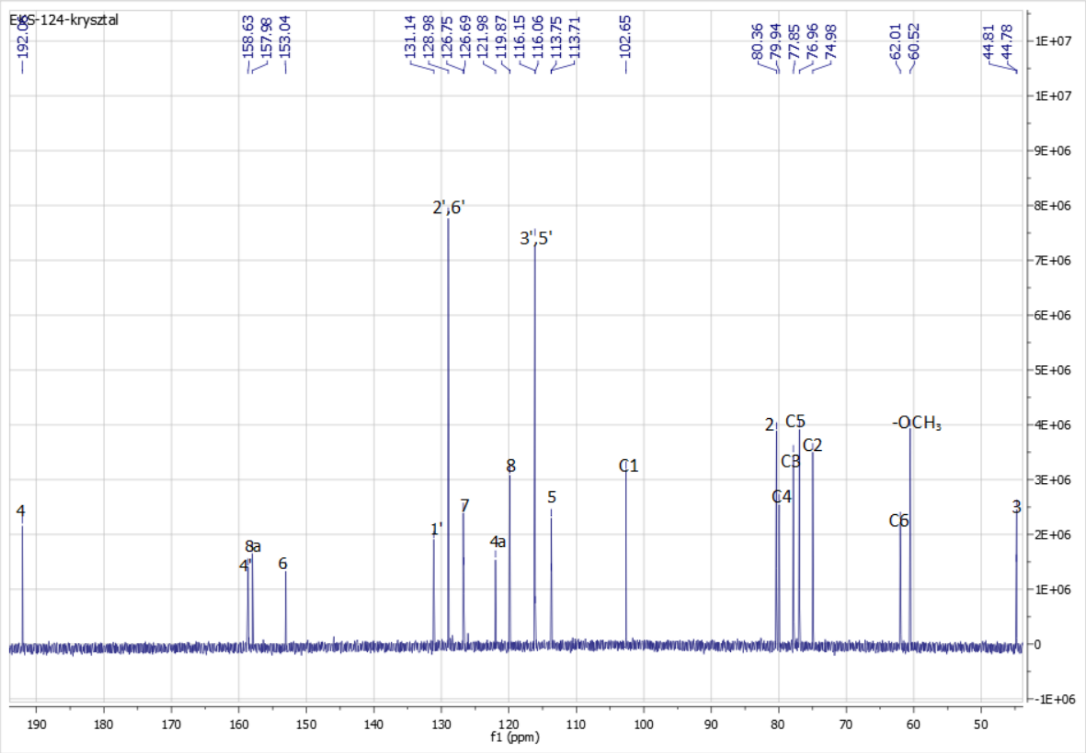

Figure S63. ^{13}C NMR spectrum of 4′-hydroxyflavanone 6-*O*-β-D-(4″-*O*-methyl)-glucopyranoside (3b) (Acetone-d$_6$, 151 MHz)

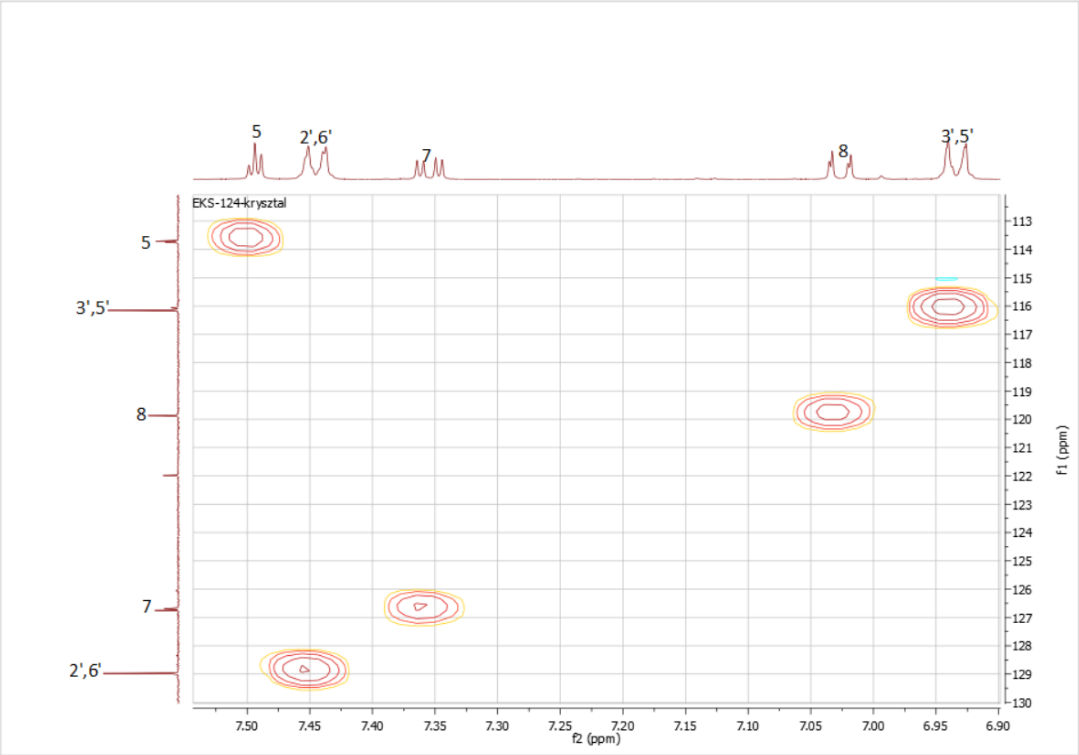

Figure S64. HSQC NMR spectrum of 4′-hydroxyflavanone 6-*O*-β-D-(4″-*O*-methyl)-glucopyranoside (3b) (Acetone-d$_6$, 151 MHz)

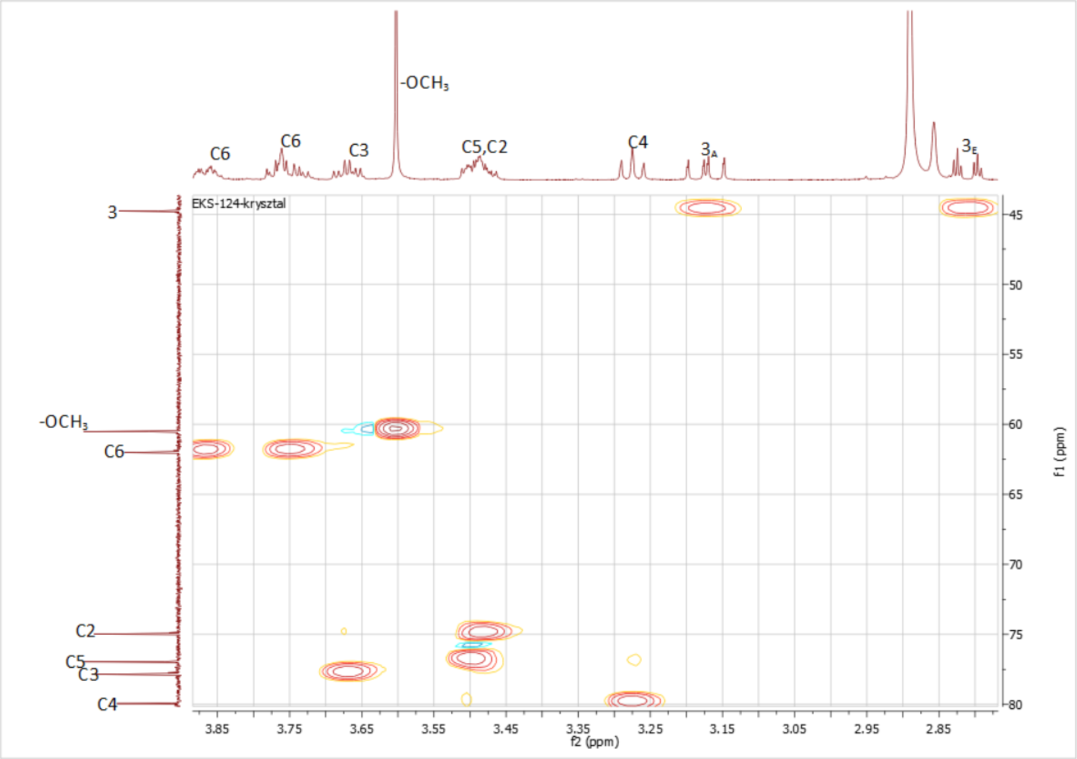

Figure S65. HSQC NMR spectrum of 4′-hydroxyflavanone 6-*O*-β-D-(4″-*O*-methyl)-glucopyranoside (3b) (Acetone-d6, 151 MHz)

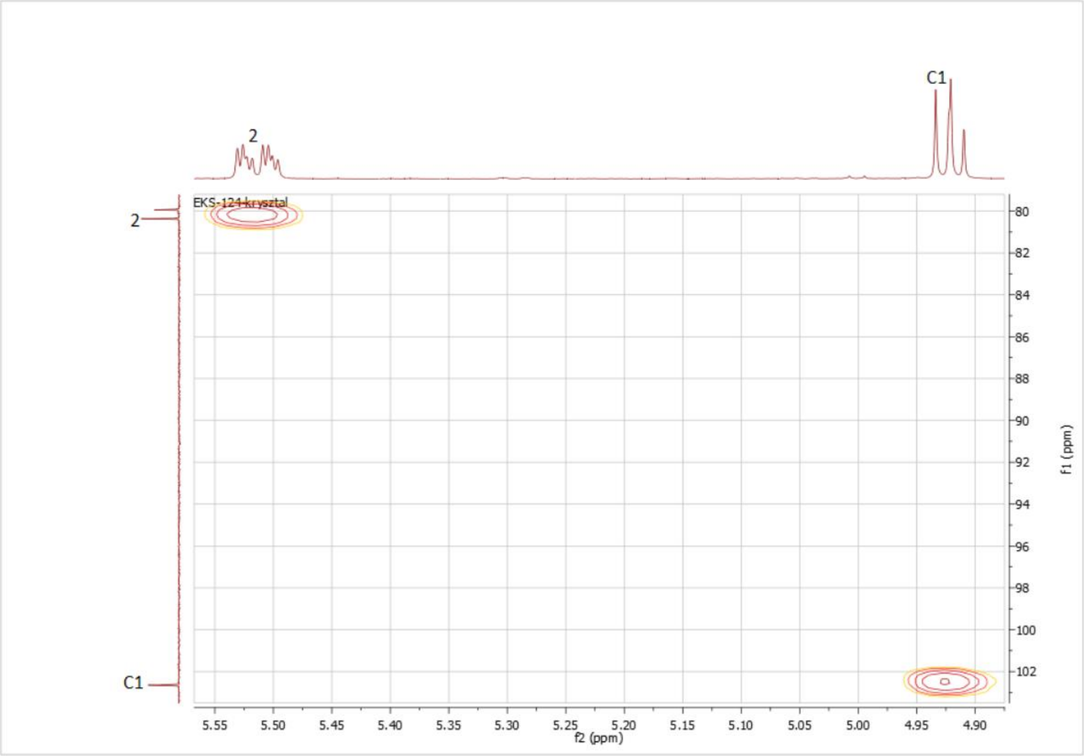

Figure S66. HSQC NMR spectrum of 4′-hydroxyflavanone 6-*O*-β-D-(4″-*O*-methyl)-glucopyranoside (3b) (Acetone-d6, 151 MHz)

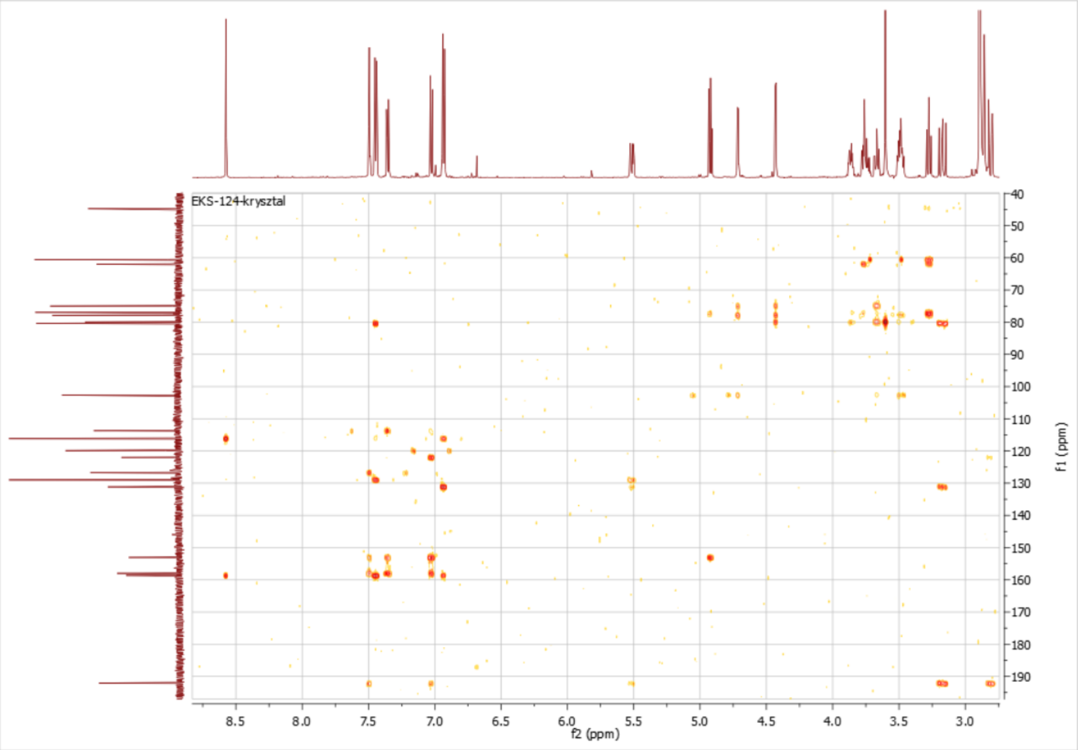

Figure S67. HMBC NMR spectrum of 4′-hydroxyflavanone 6-*O*-β-D-(4″-*O*-methyl)-glucopyranoside (3b) (Acetone-d$_6$, 151 MHz)

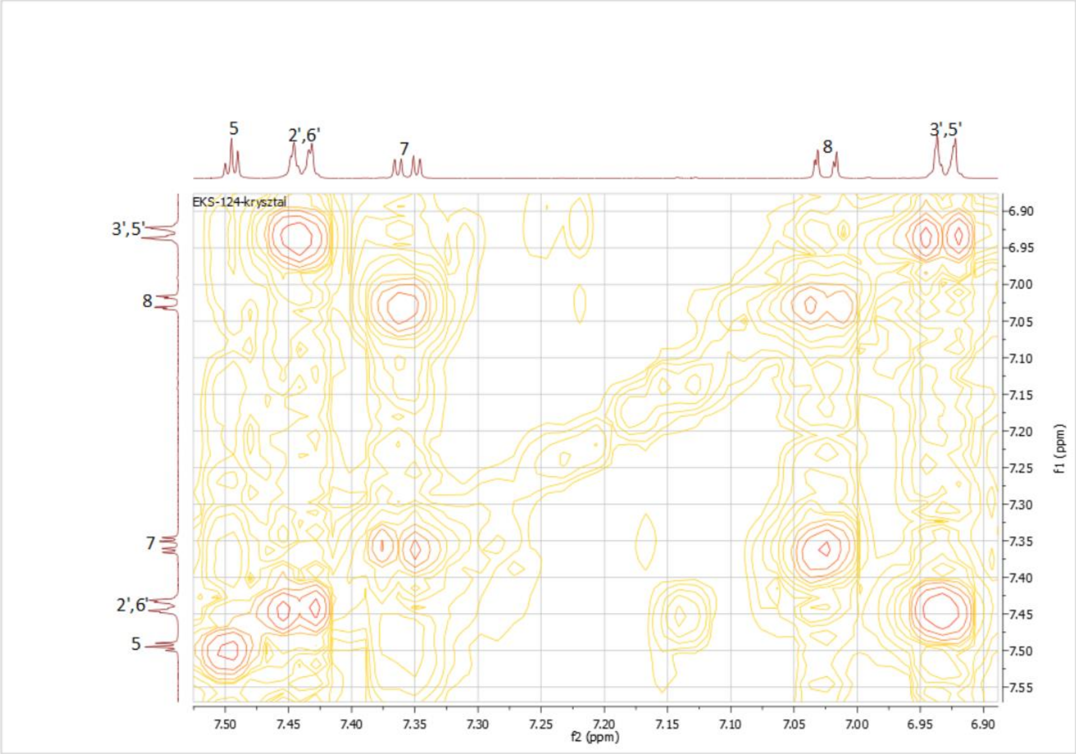

Figure S68. COSY NMR spectrum of 4′-hydroxyflavanone 6-*O*-β-D-(4″-*O*-methyl)-glucopyranoside (3b) (Acetone-d$_6$, 600 MHz)

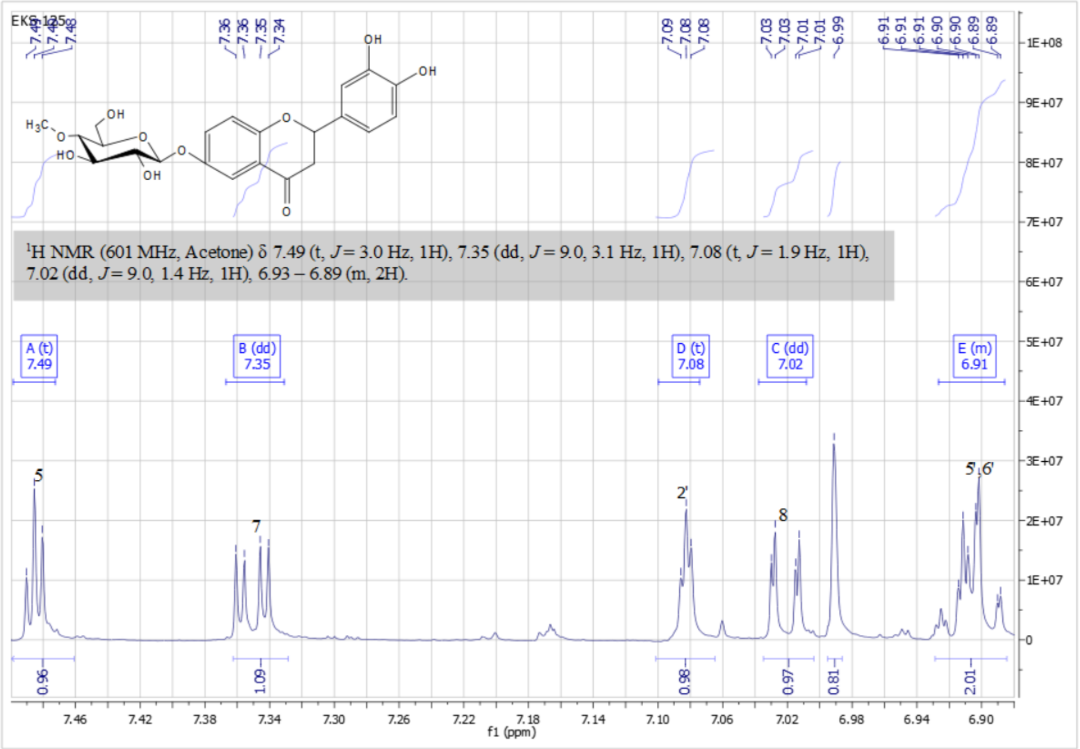

Figure S69. ¹H NMR spectrum of 3′,4′-dihydroxyflavanone 6-*O*-β-D-(4″-*O*-methyl)-glucopyranoside (3c) (Acetone-d₆, 600 MHz)

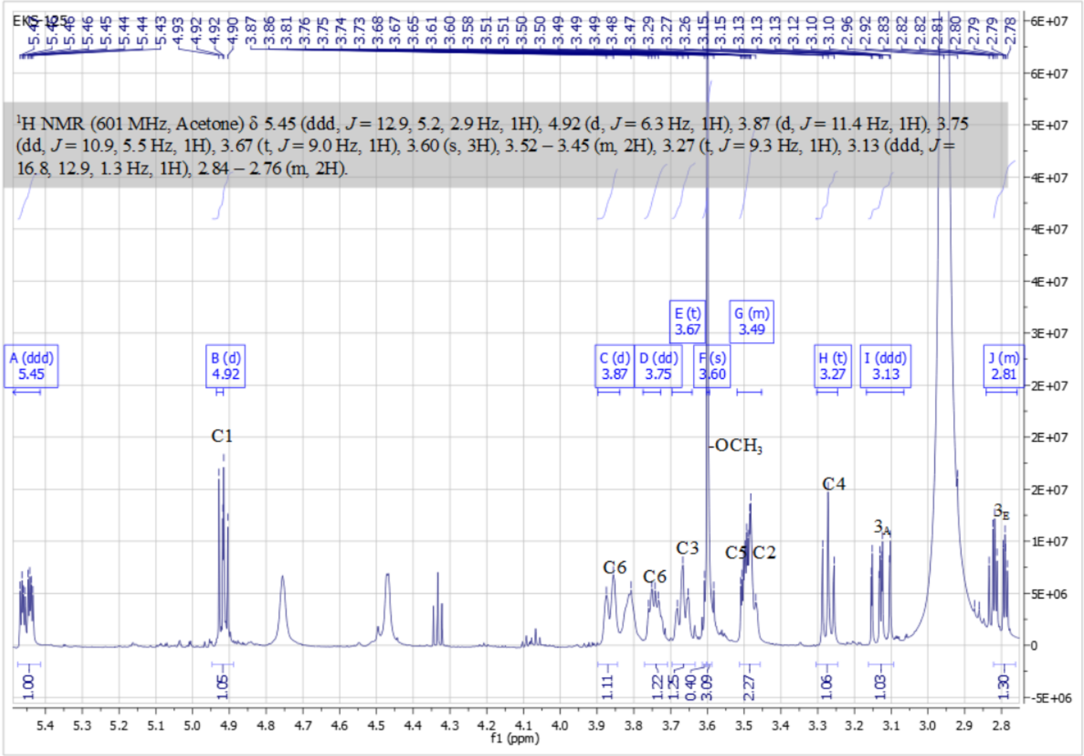

Figure S70. ¹H NMR spectrum of 3′,4′-dihydroxyflavanone 6-*O*-β-D-(4″-*O*-methyl)-glucopyranoside (3c) (Acetone-d₆, 600 MHz)

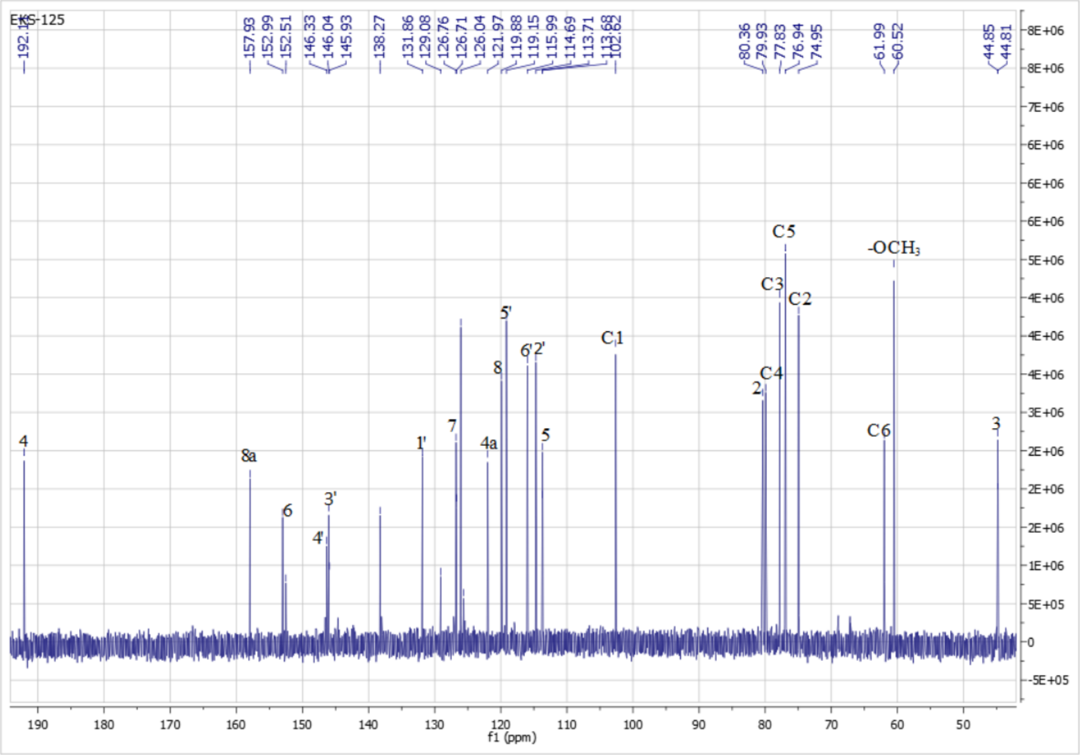

Figure S71. ^{13}C NMR spectrum of 3′,4′-dihydroxyflavanone 6-*O*-β-D-(4′′-*O*-methyl)-glucopyranoside (3c) (Acetone-d$_6$, 151 MHz)

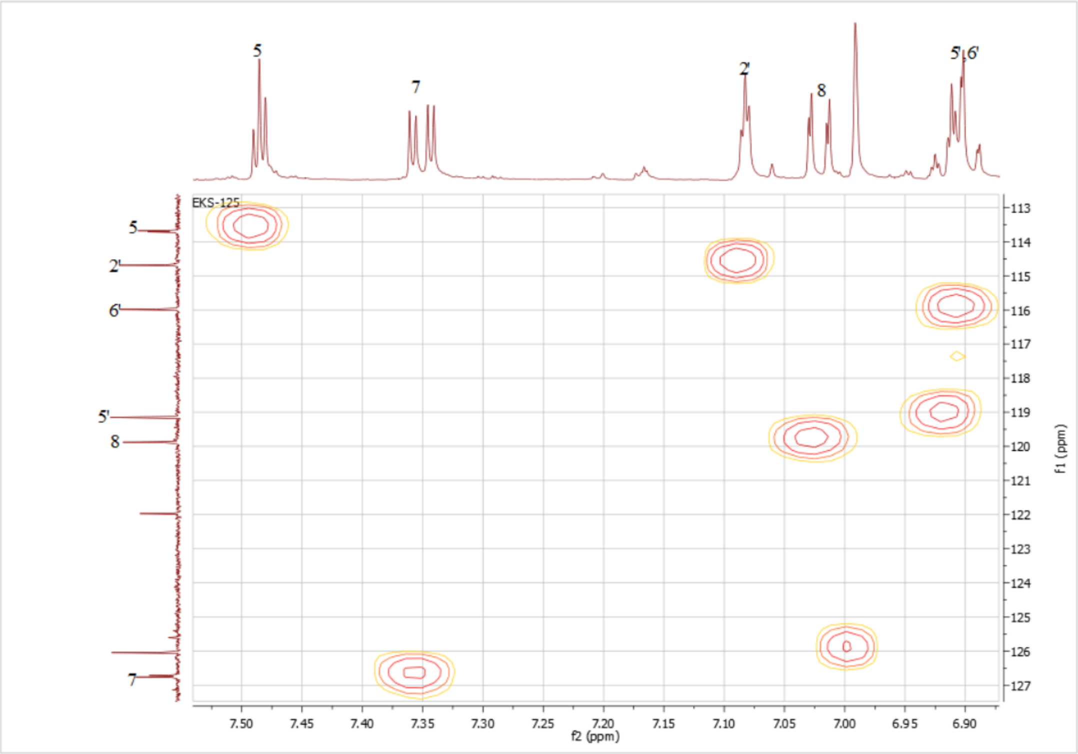

Figure S72. HSQC NMR spectrum of 3′,4′-dihydroxyflavanone 6-*O*-β-D-(4′′-*O*-methyl)-glucopyranoside (3c) (Acetone-d$_6$, 151 MHz)

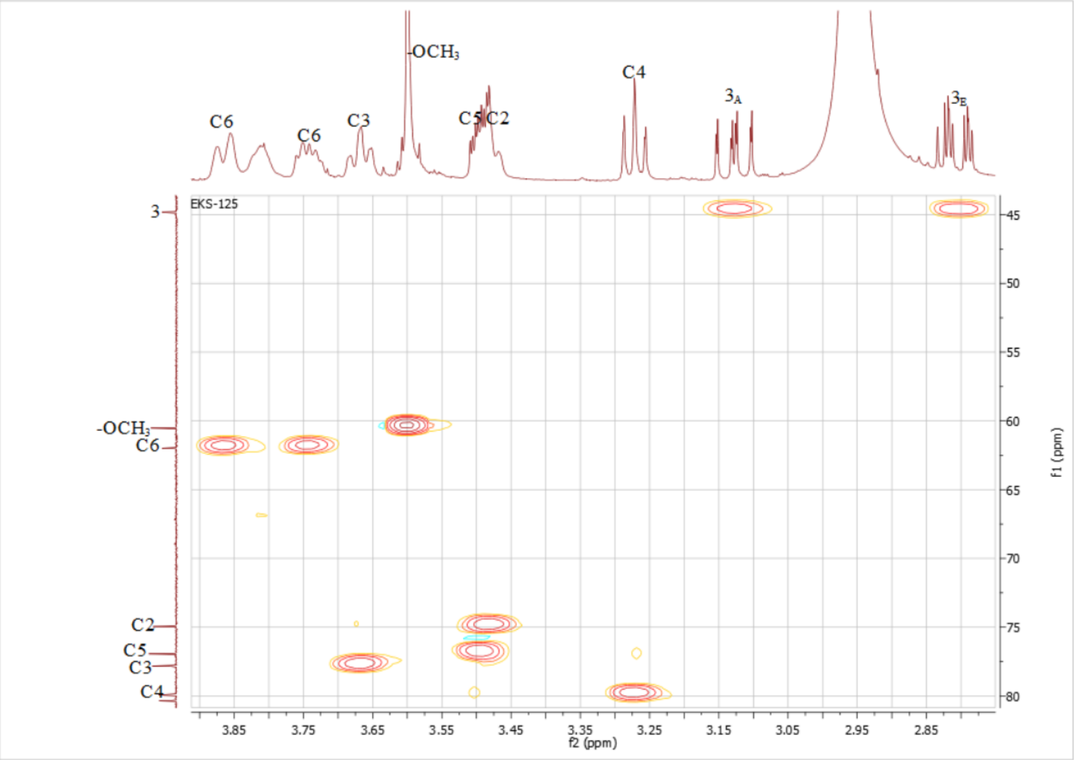

Figure S73. HSQC NMR spectrum of 3′,4′-dihydroxyflavanone 6-*O*-β-D-(4″-*O*-methyl)-glucopyranoside (3c) (Acetone-d$_6$, 151 MHz)

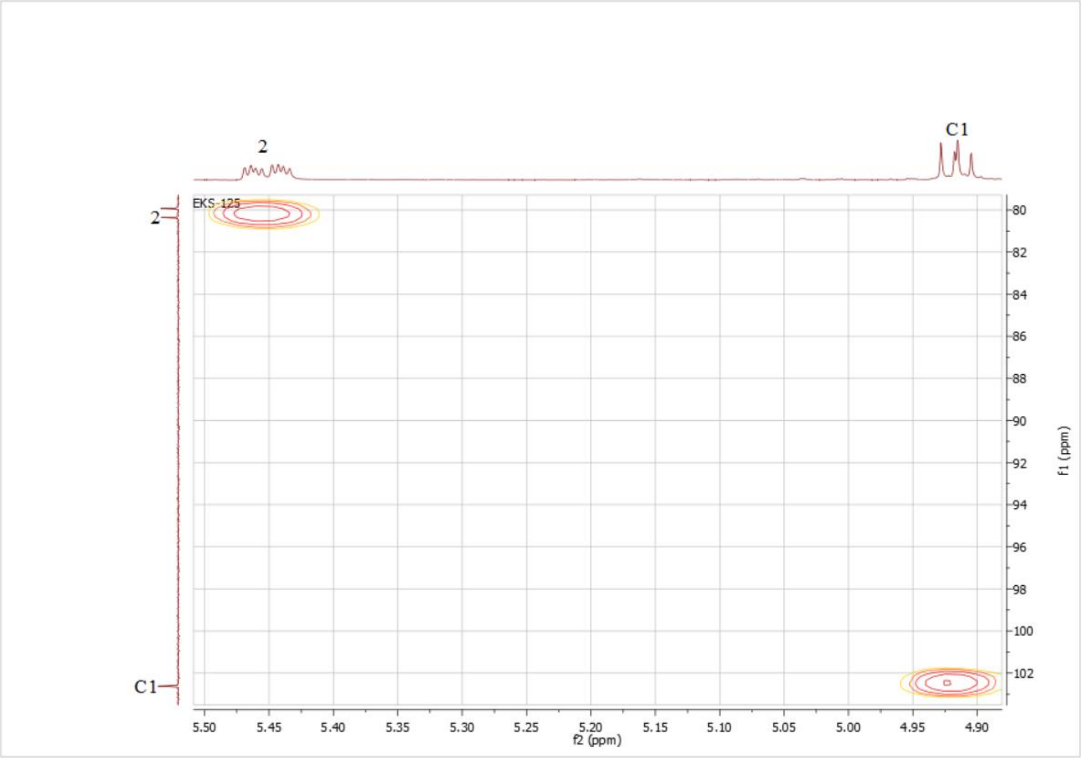

Figure S74. HSQC NMR spectrum of 3′,4′-dihydroxyflavanone 6-*O*-β-D-(4″-*O*-methyl)-glucopyranoside (3c) (Acetone-d$_6$, 151 MHz)

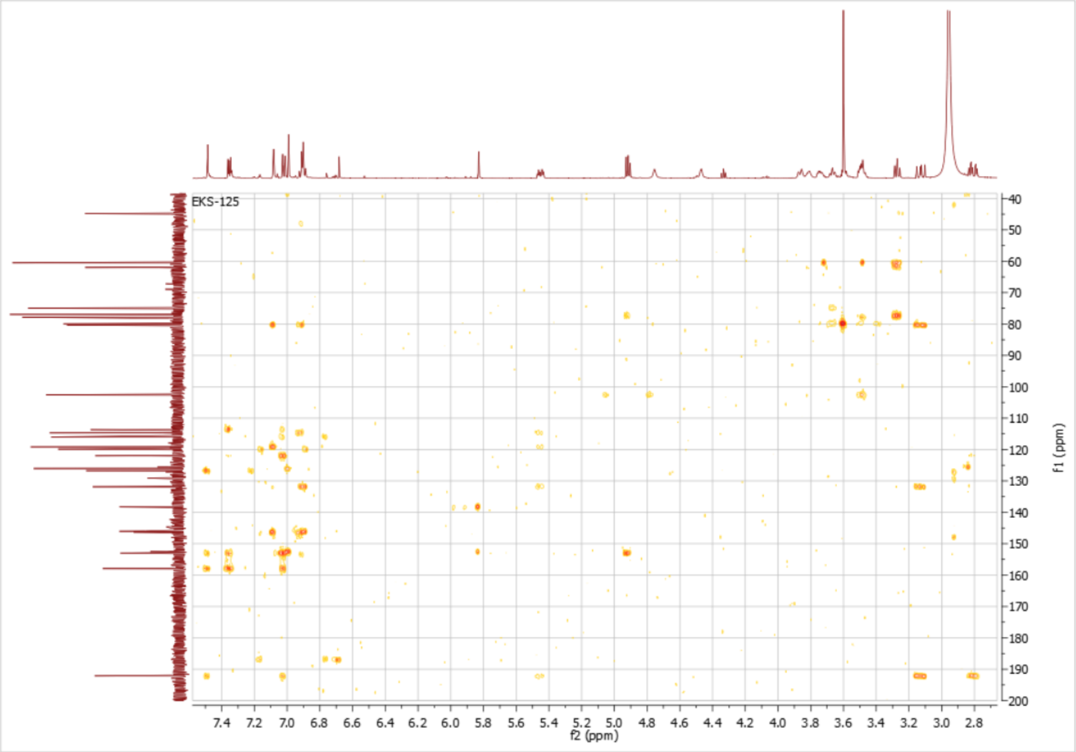

Figure S75. HMBC NMR spectrum of 3′,4′-dihydroxyflavanone 6-*O*-β-D-(4″-*O*-methyl)-glucopyranoside (3c) (Acetone-d_6, 151 MHz)

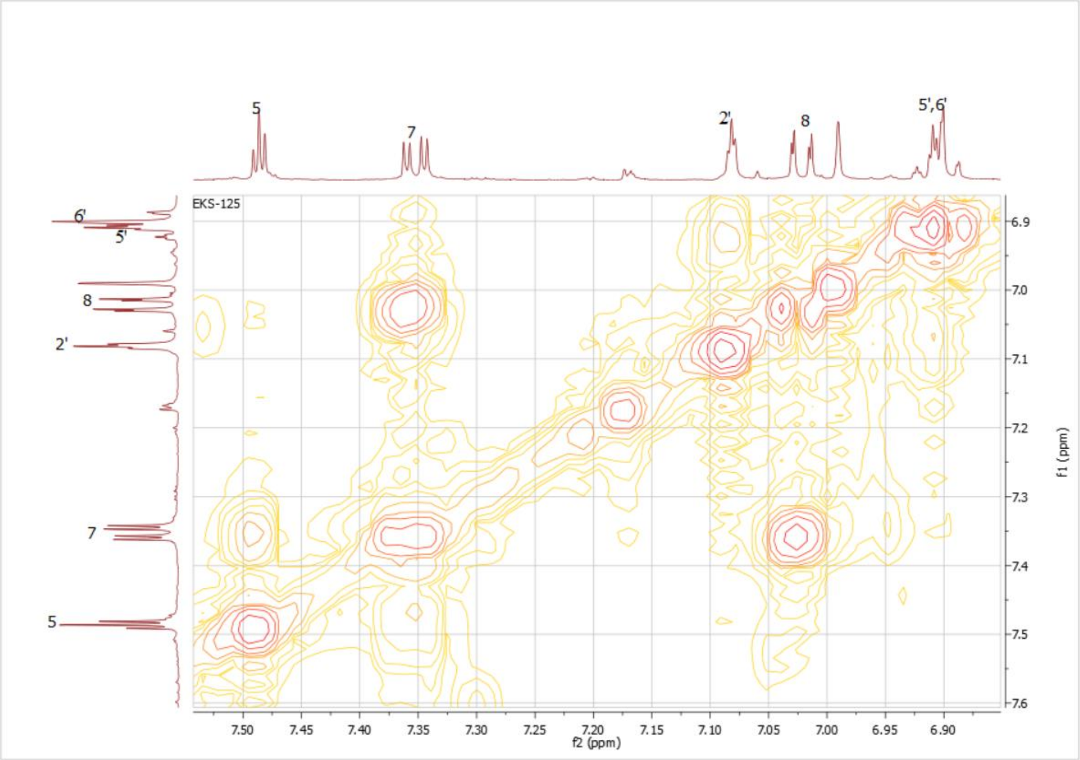

Figure S76. COSY NMR spectrum of 3′,4′-dihydroxyflavanone 6-*O*-β-D-(4″-*O*-methyl)-glucopyranoside (3c) (Ac etone-d_6, 600 MHz)

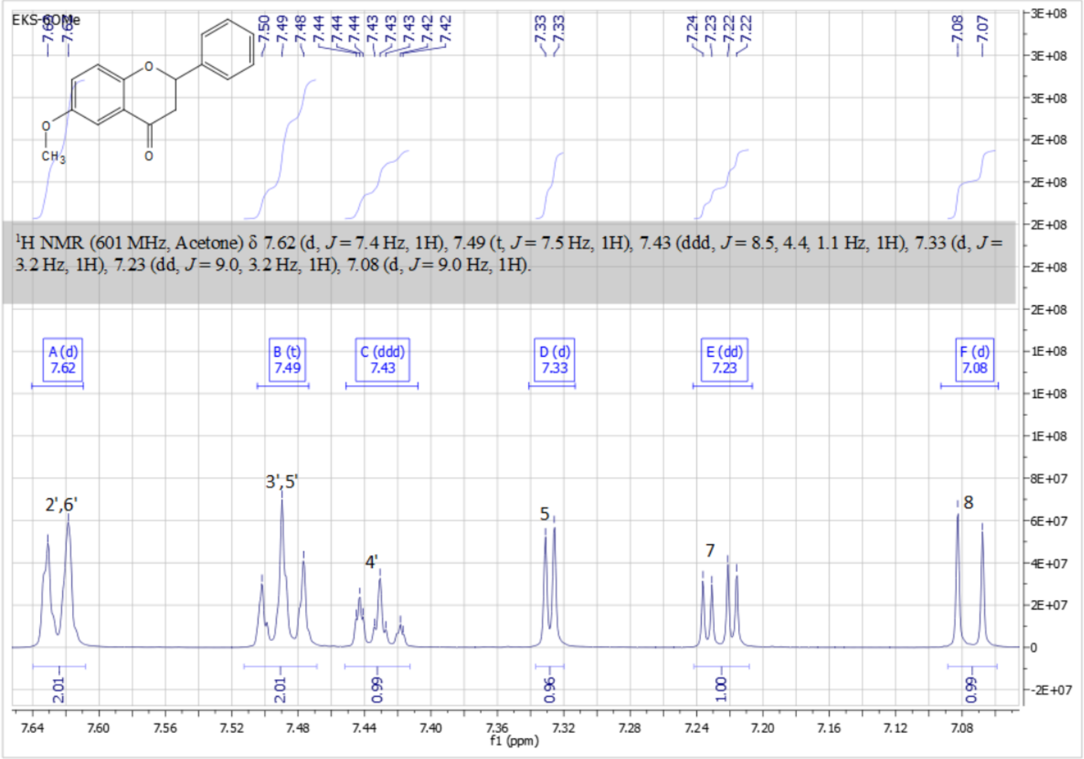

Figure S77. ^{1}H NMR spectrum of 6-methoxyflavanone (4) (Acetone-d_6, 600 MHz)

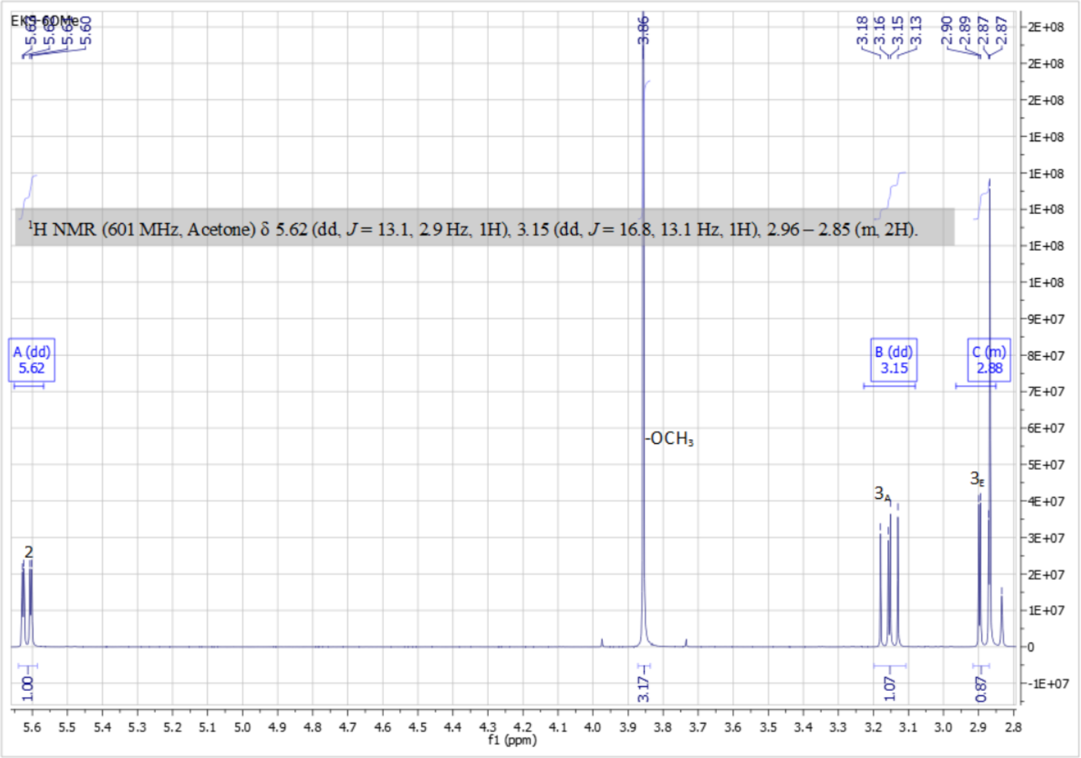

Figure S78. ^{1}H NMR spectrum of 6-methoxyflavanone (4) (Acetone-d_6, 600 MHz)

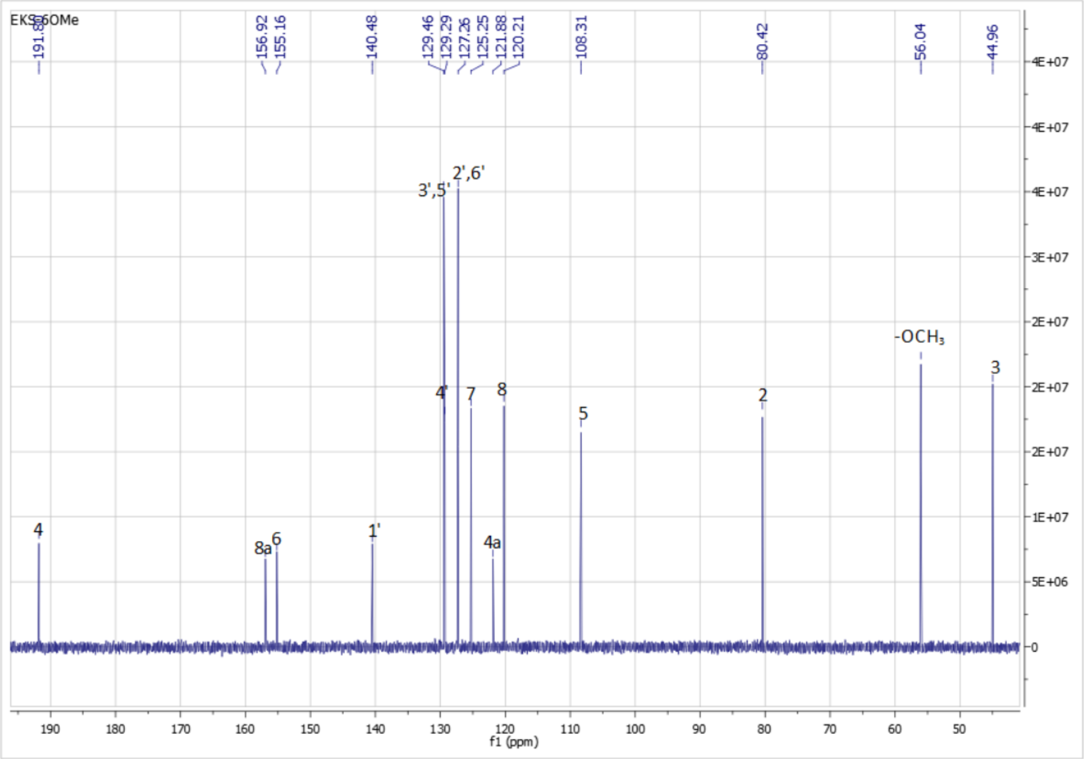

Figure S79. ^{13}C NMR spectrum of 6-methoxyflavanone (4) (Acetone-d_6, 151 MHz)

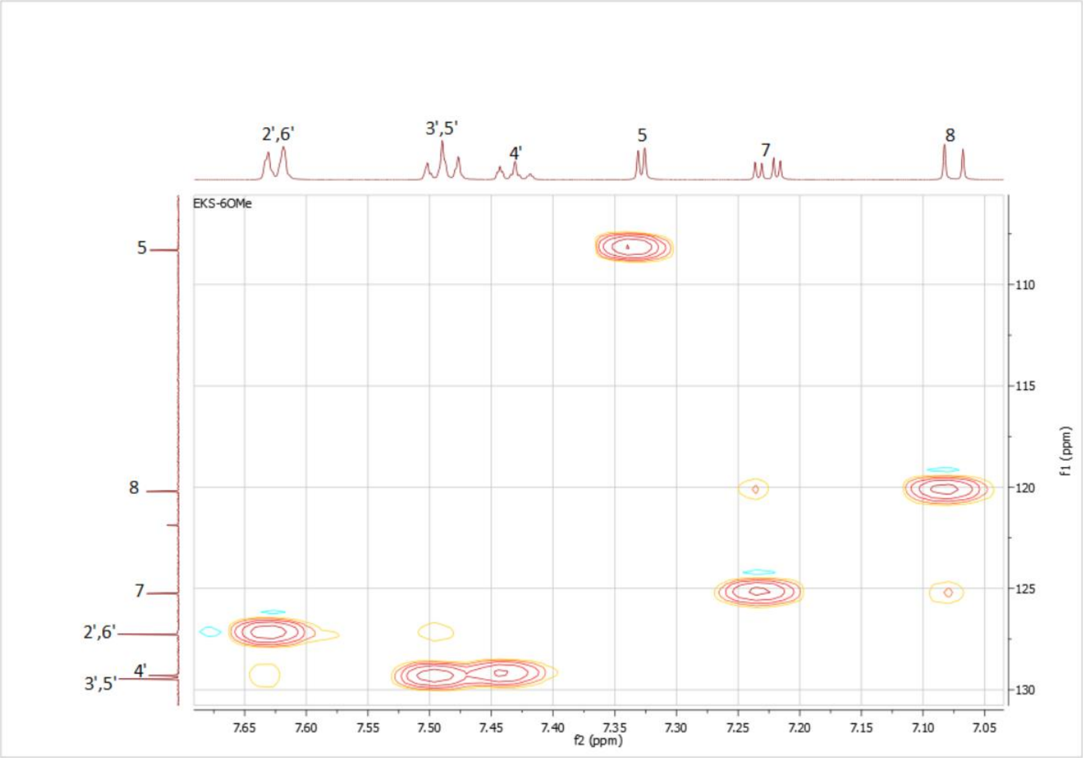

Figure S80. HSQC NMR spectrum of 6-methoxyflavanone (4) (Acetone-d_6, 151 MHz)

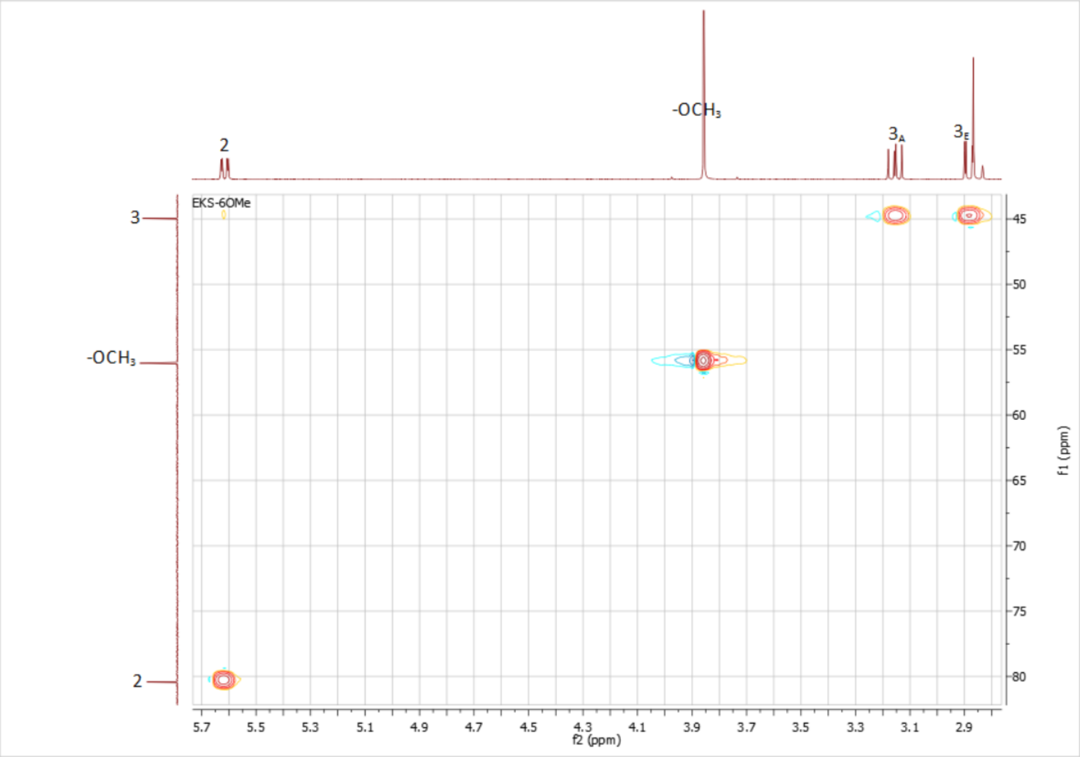

Figure S81. HSQC NMR spectrum of 6-methoxyflavanone (4) (Acetone-d6, 151 MHz)

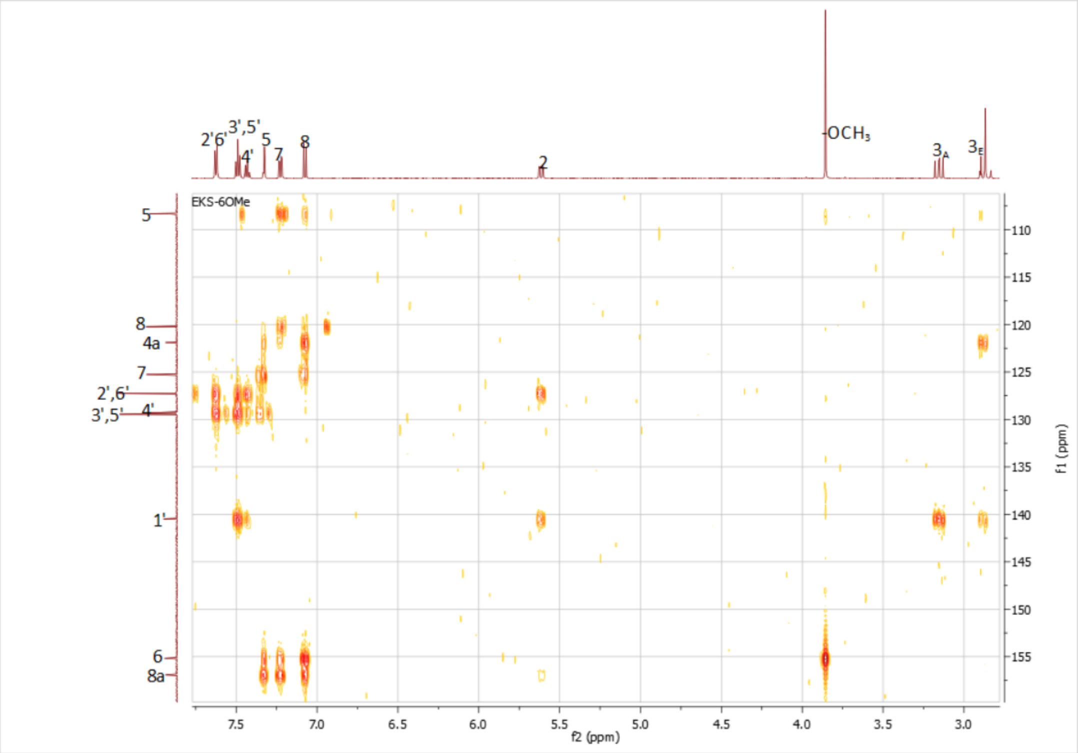

Figure S82. HMBC NMR spectrum of 6-methoxyflavanone (4) (Acetone-d6, 151 MHz)

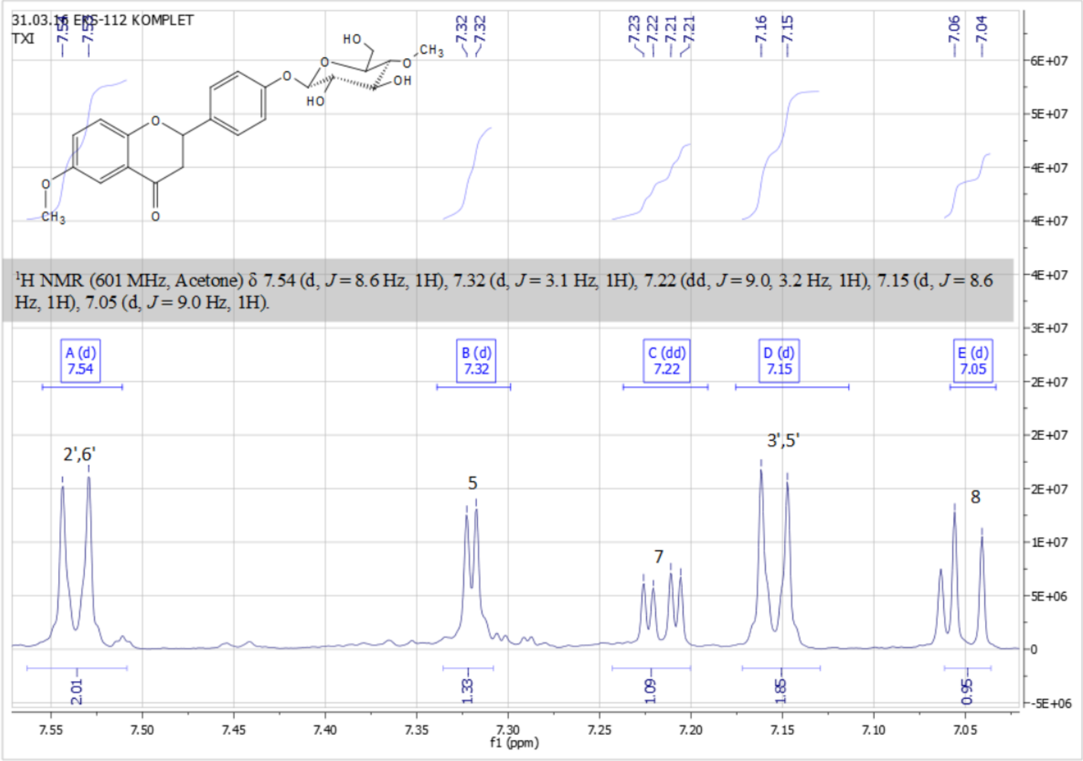

Figure S83. ^{1}H NMR spectrum of 6-methoxyflavanone 4′-*O*-β-D-(4″-*O*-methyl)-glucopyranoside (4a) (Acetone-d$_6$, 600 MHz)

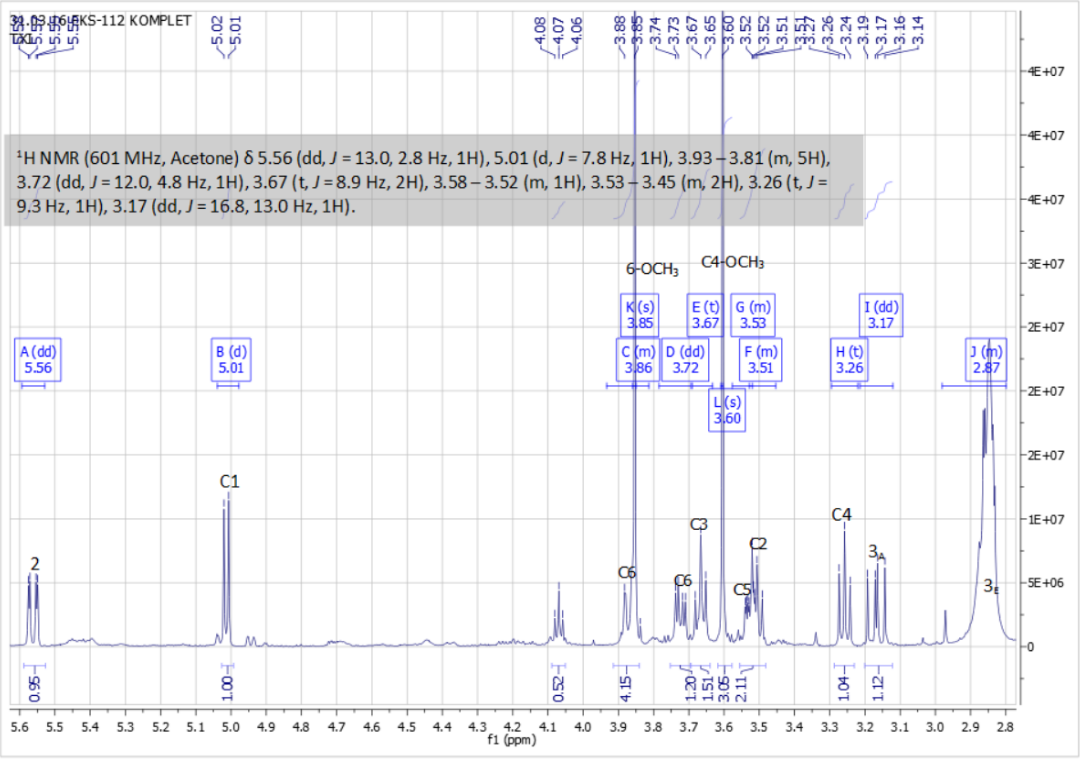

Figure S84. ^{1}H NMR spectrum of 6-methoxyflavanone 4′-*O*-β-D-(4″-*O*-methyl)-glucopyranoside (4a) (Acetone-d$_6$, 600 MHz)

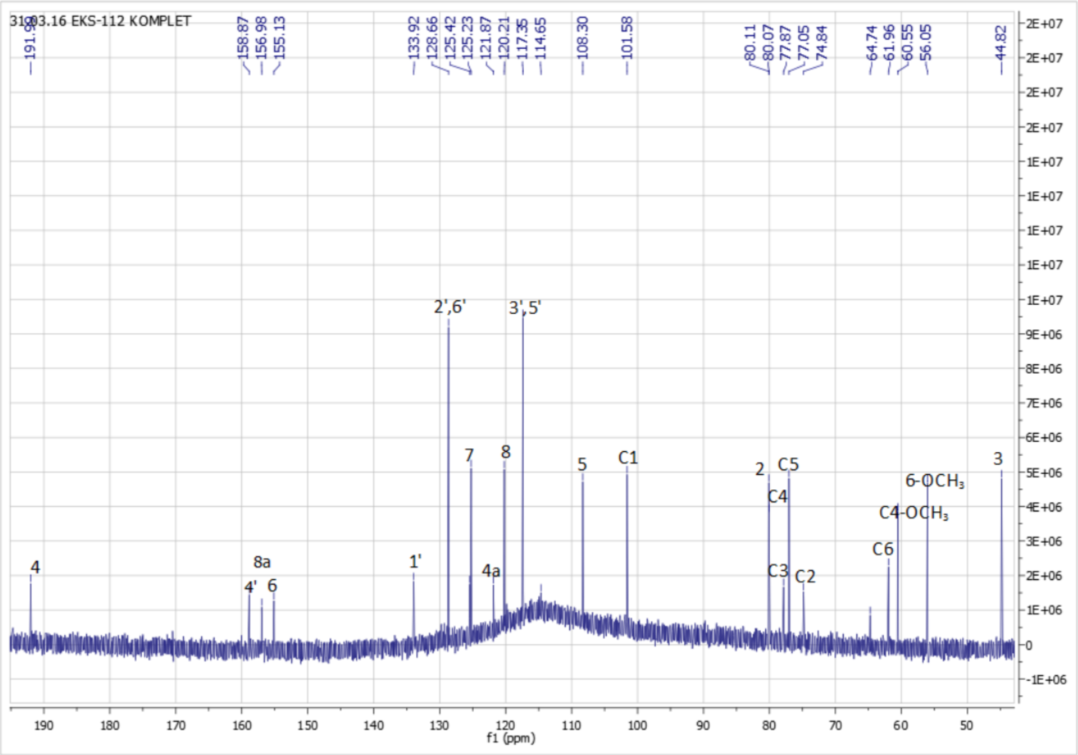

Figure S85. ^{13}C NMR spectrum of 6-methoxyflavanone 4′-*O*-β-D-(4″-*O*-methyl)-glucopyranoside (4a) (Acetone-d_6, 151 MHz)

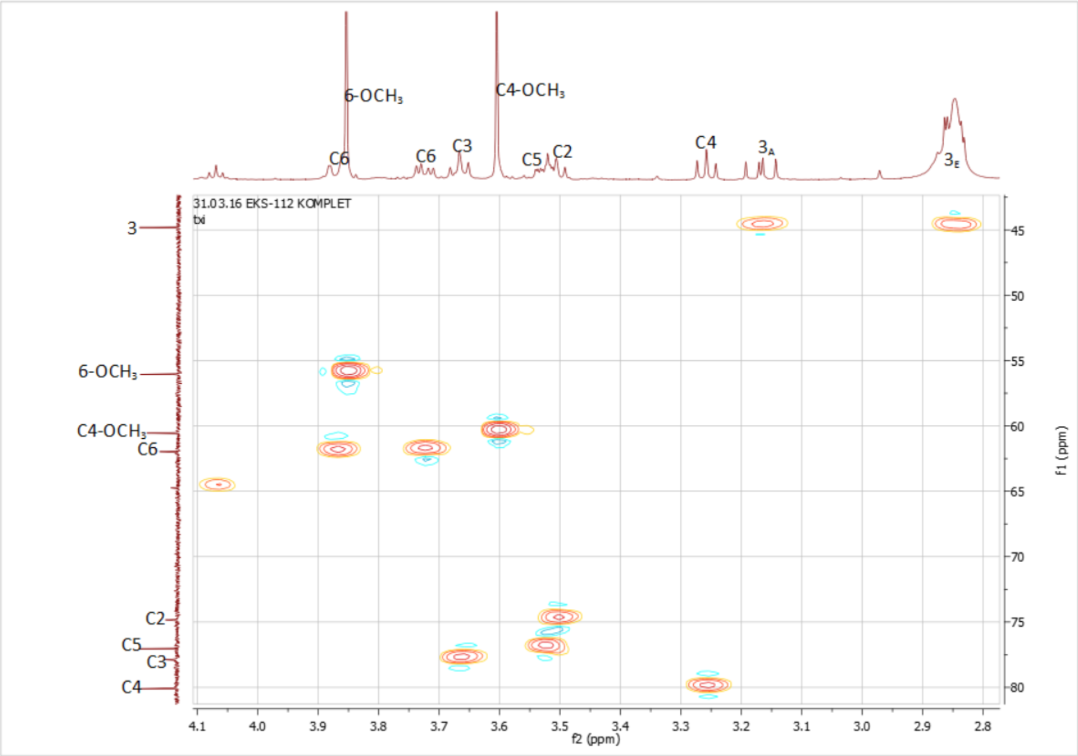

Figure S86. HSQC NMR spectrum of 6-methoxyflavanone 4′-*O*-β-D-(4″-*O*-methyl)-glucopyranoside (4a) (Acetone-d_6, 151 MHz)

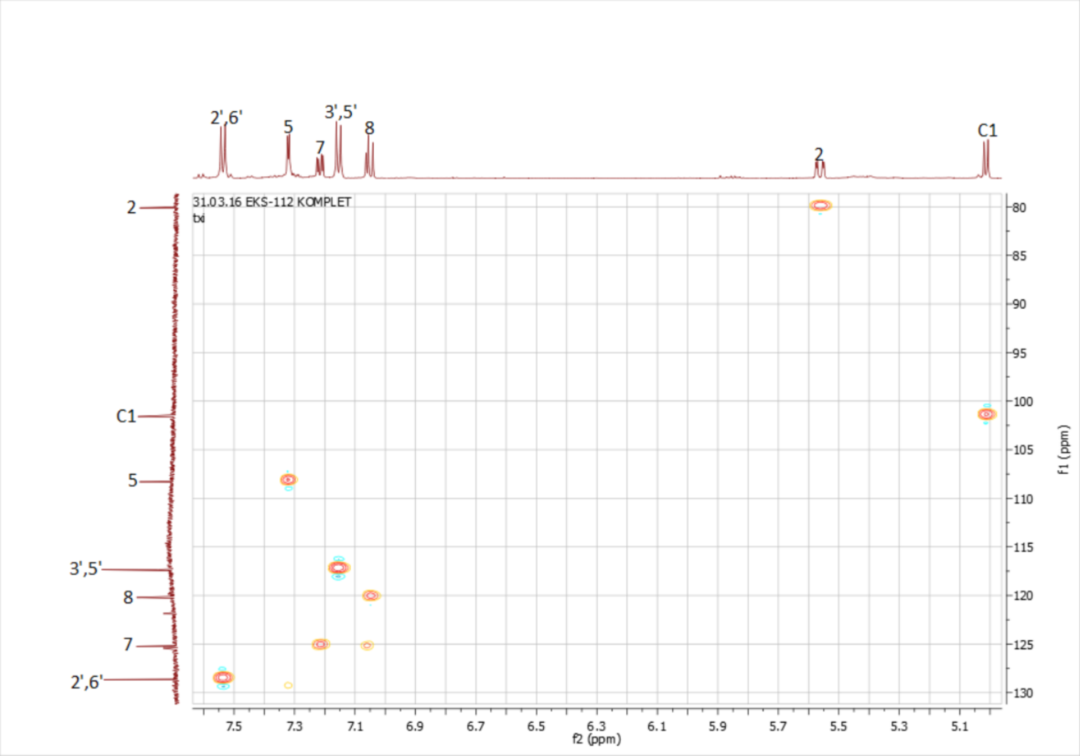

Figure S87. HSQC NMR spectrum of 6-methoxyflavanone 4′-*O*-β-D-(4″-*O*-methyl)-glucopyranoside (4a) (Acetone-d_6, 151 MHz)

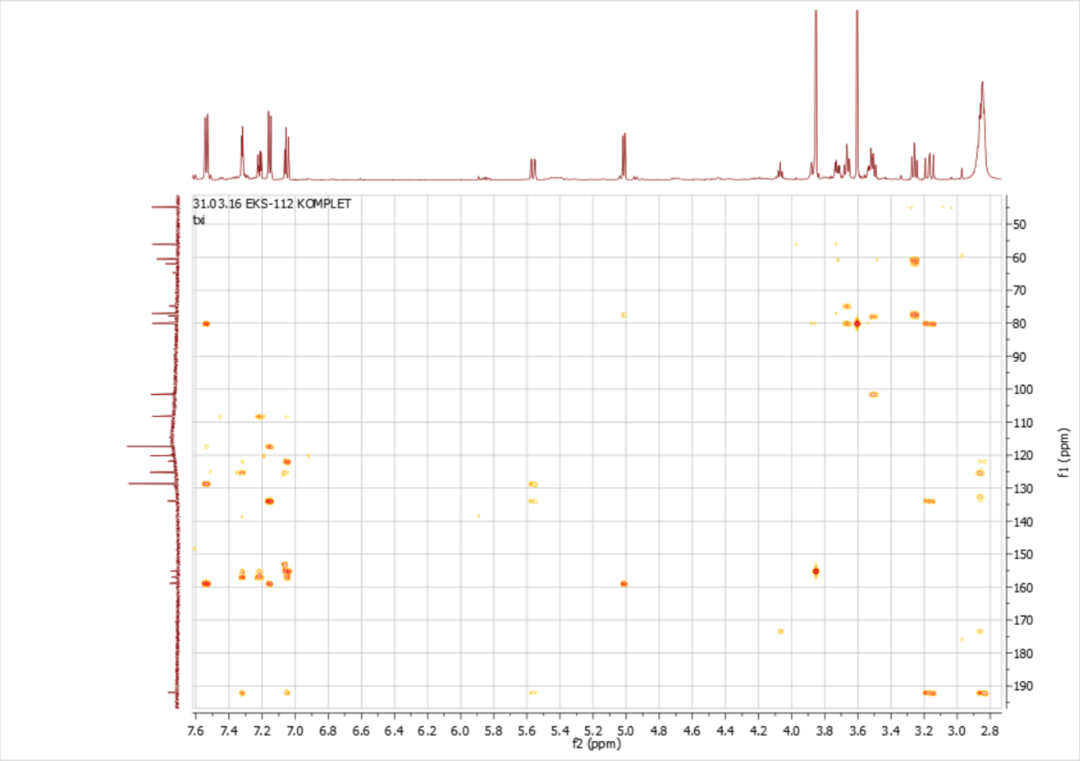

Figure S88. HMBC NMR spectrum of 6-methoxyflavanone 4′-*O*-β-D-(4″-*O*-methyl)-glucopyranoside (4a) (Acetone-d_6, 151 MHz)

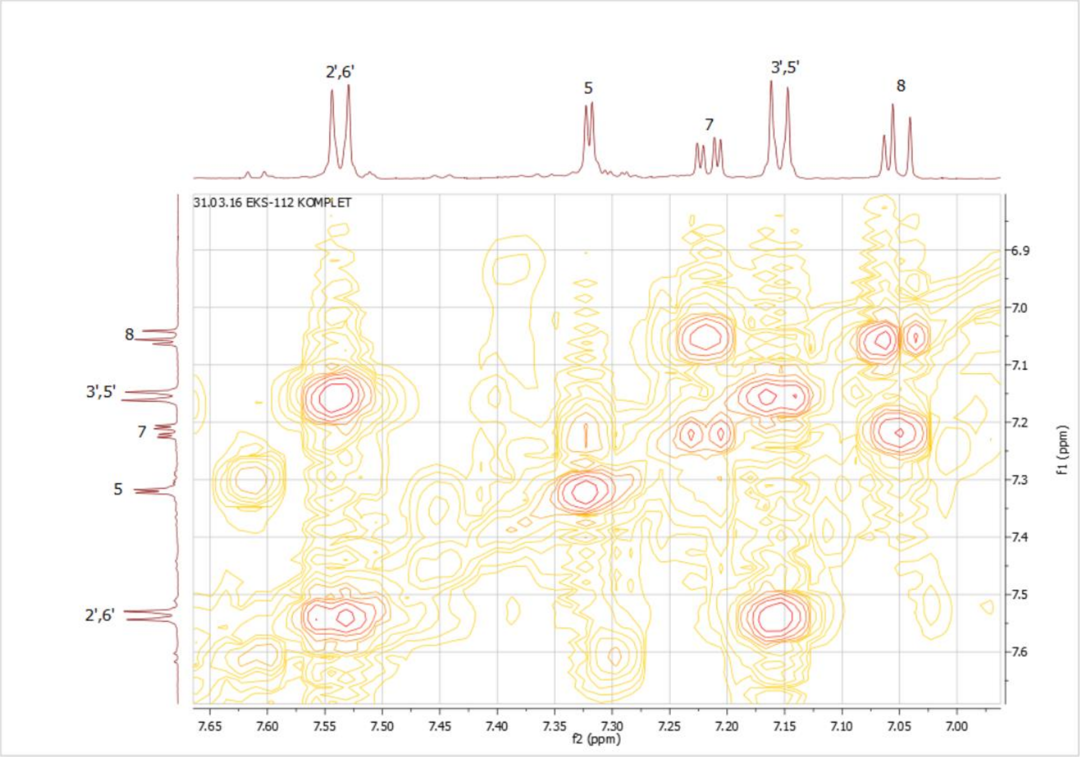

Figure S89. COSY NMR spectrum of 6-methoxyflavanone 4′-*O*-β-D-(4″-*O*-methyl)-glucopyranoside (4a) (Acetone-d6, 600 MHz)

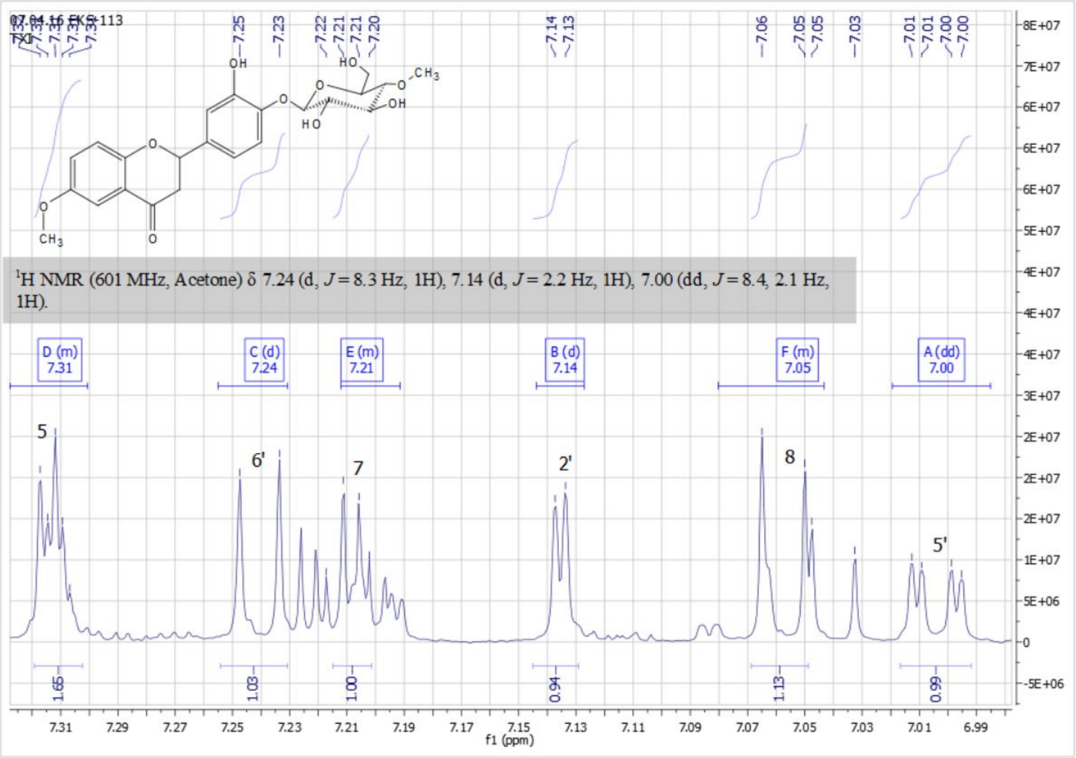

Figure S90. ^{1}H NMR spectrum of 3′-hydroxy-6-methoxyflavanone 4′-*O*-β-D-(4″-*O*-methyl)-glucopyranoside (4b) (Acetone-d6, 600 MHz)

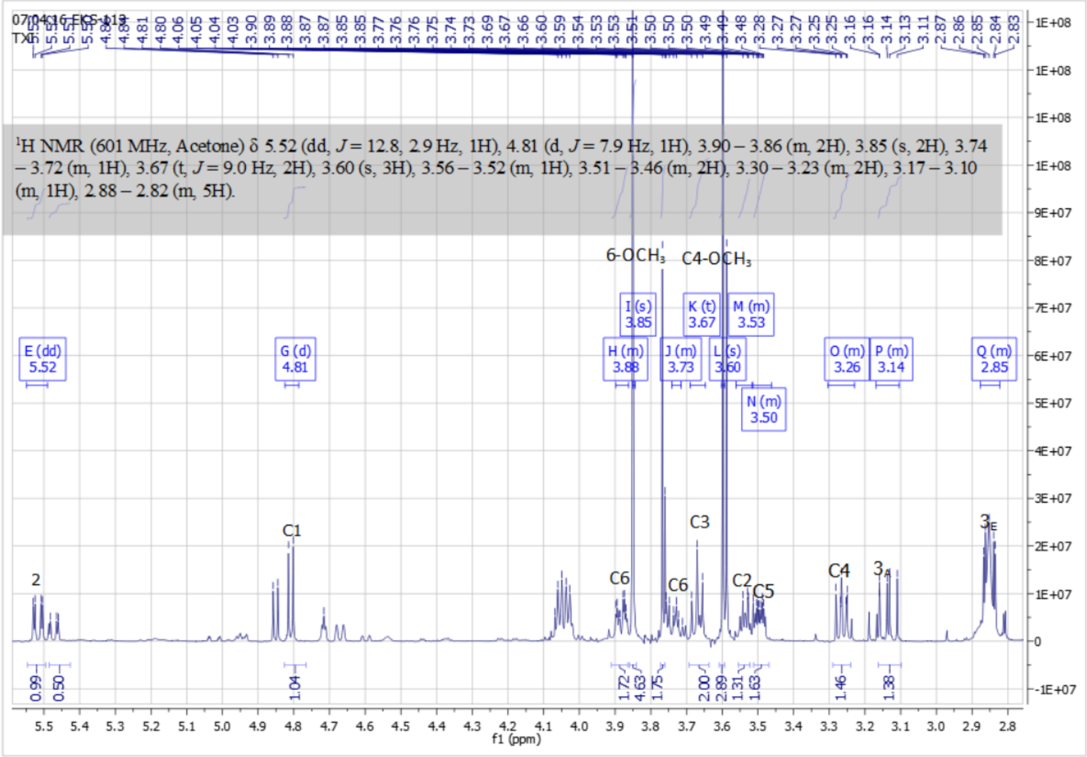

Figure S91. ^{1}H NMR spectrum of 3′-hydroxy-6-methoxyflavanone 4′-*O*-β-D-(4″-*O*-methyl)-glucopyranoside (4b) (Acetone-d6, 600 MHz)

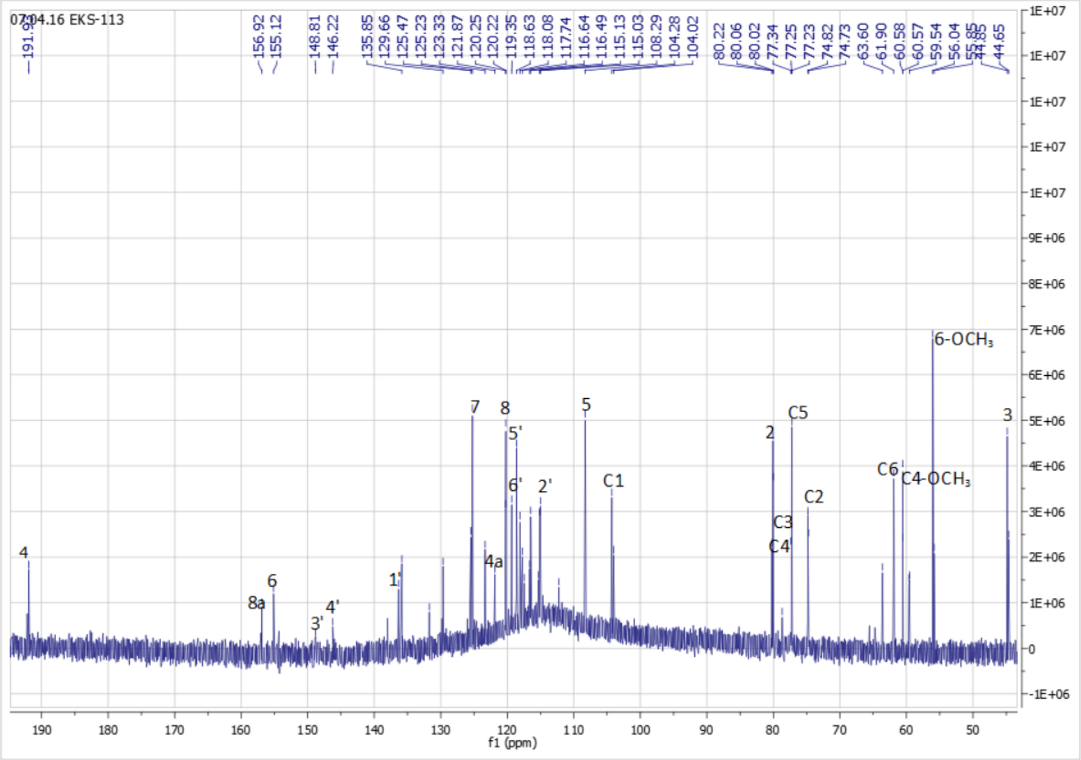

Figure S92. ^{13}C NMR spectrum of 3′-hydroxy-6-methoxyflavanone 4′-*O*-β-D-(4″-*O*-methyl)-glucopyranoside (4b) (Acetone-d6, 151 MHz)

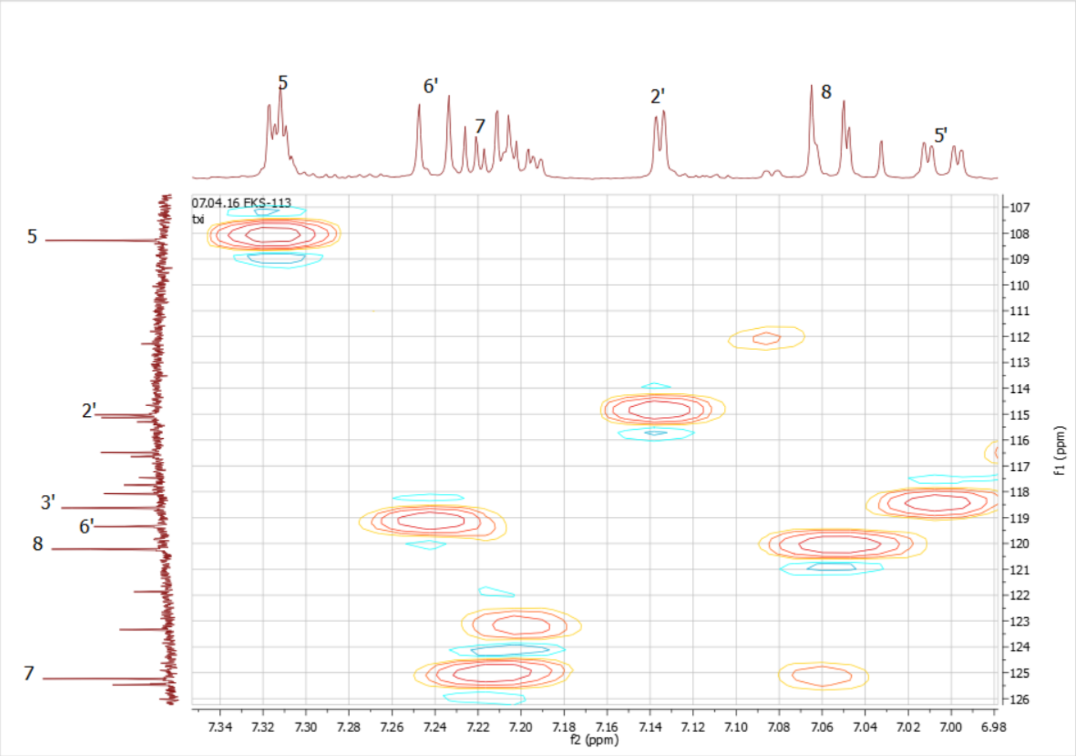

Figure S93. HSQC NMR spectrum of 3′-hydroxy-6-methoxyflavanone 4′-*O*-β-D-(4″-*O*-methyl)-glucopyranoside (4b) (Acetone-d₆, 151 MHz)

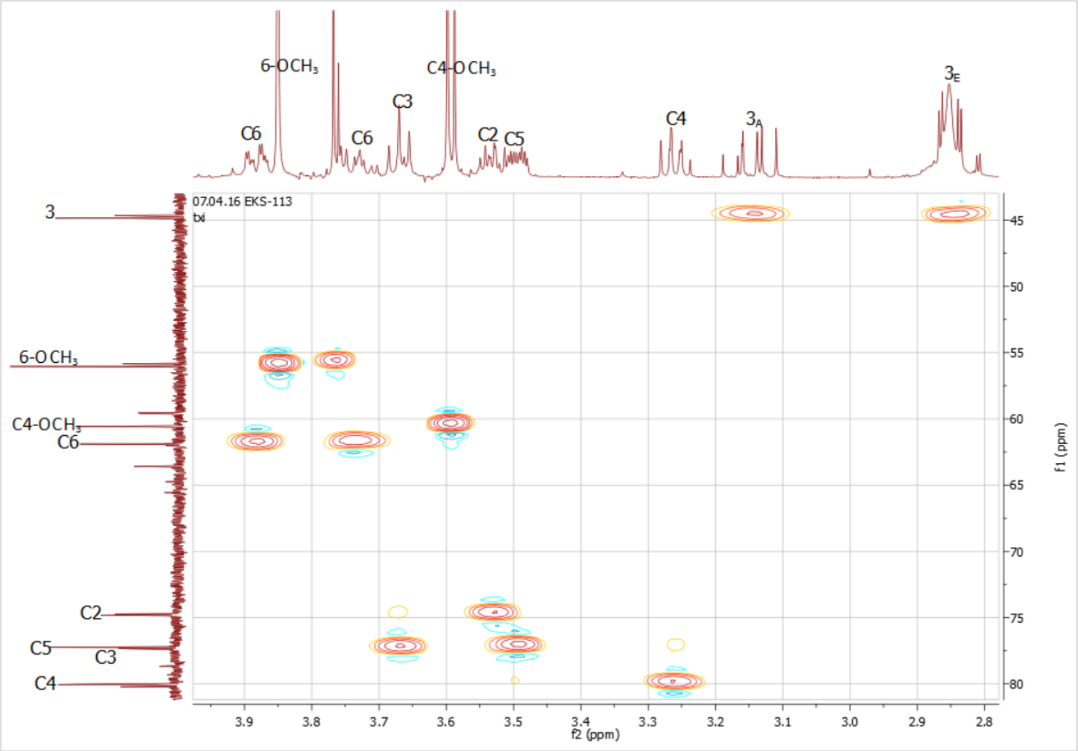

Figure S94. HSQC NMR spectrum of 3′-hydroxy-6-methoxyflavanone 4′-*O*-β-D-(4″-*O*-methyl)-glucopyranoside (4b) (Acetone-d₆, 151 MHz)

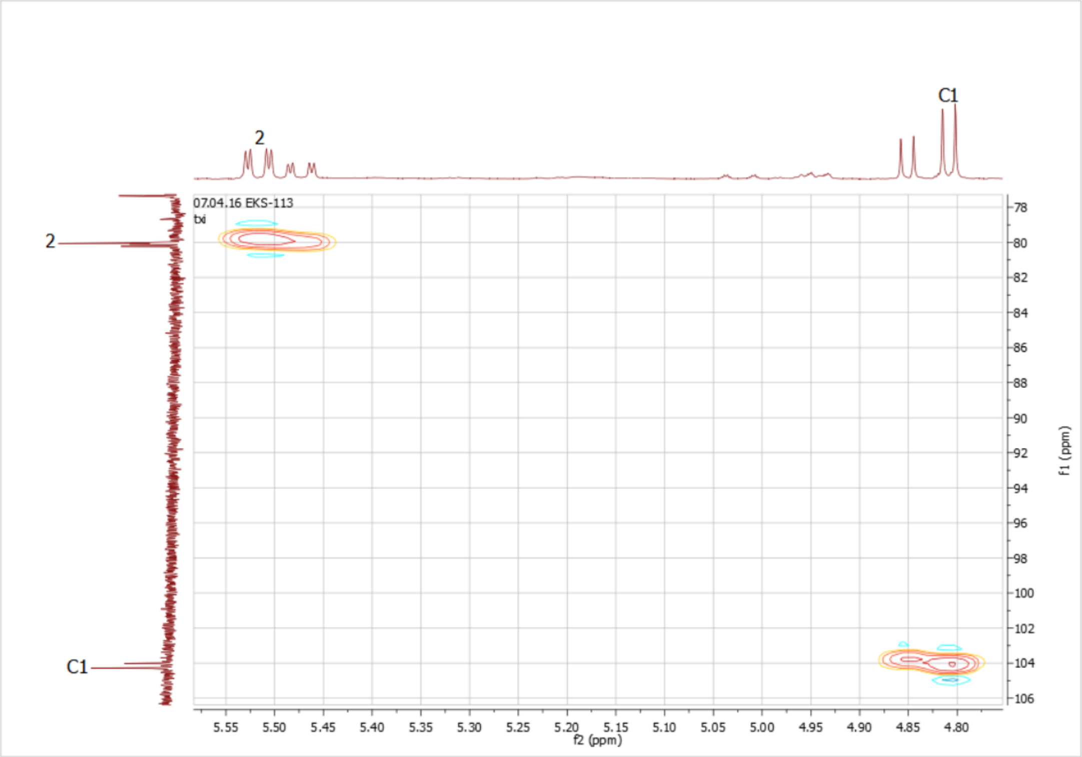

Figure S95. HSQC NMR spectrum of 3′-hydroxy-6-methoxyflavanone 4′-*O*-β-D-(4′′-*O*-methyl)-glucopyranoside (4b) (Acetone-d₆, 151 MHz)

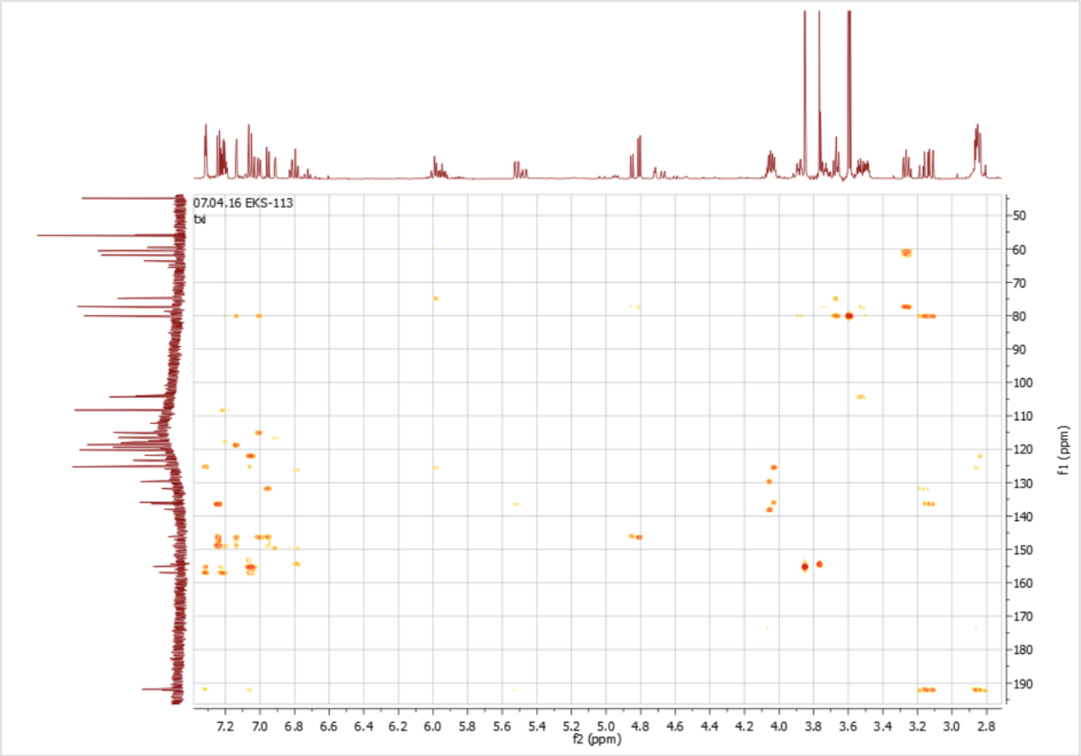

Figure S96. HMBC NMR spectrum of 3′-hydroxy-6-methoxyflavanone 4′-*O*-β-D-(4′′-*O*-methyl)-glucopyranoside (4b) (Acetone-d₆, 151 MHz)

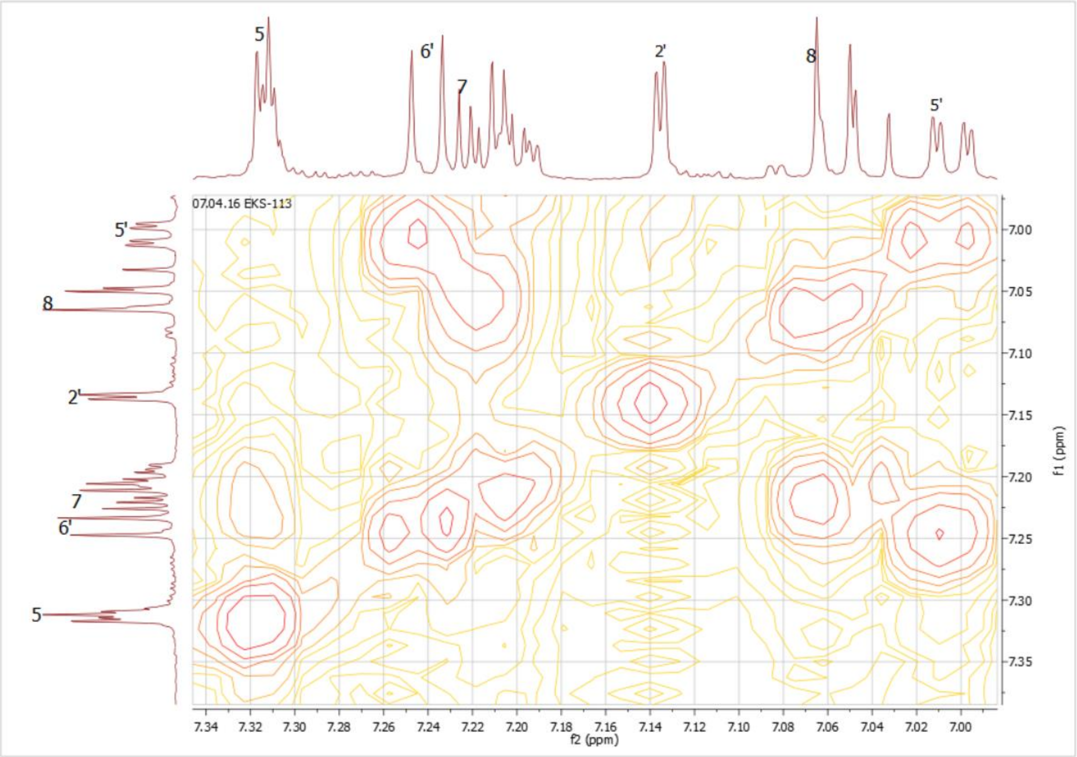

Figure S97. COSY NMR spectrum of 3′-hydroxy-6-methoxyflavanone 4′-*O*-β-D-(4″-*O*-methyl)-glucopyranoside (4b) (Acetone-d6, 600 MHz)

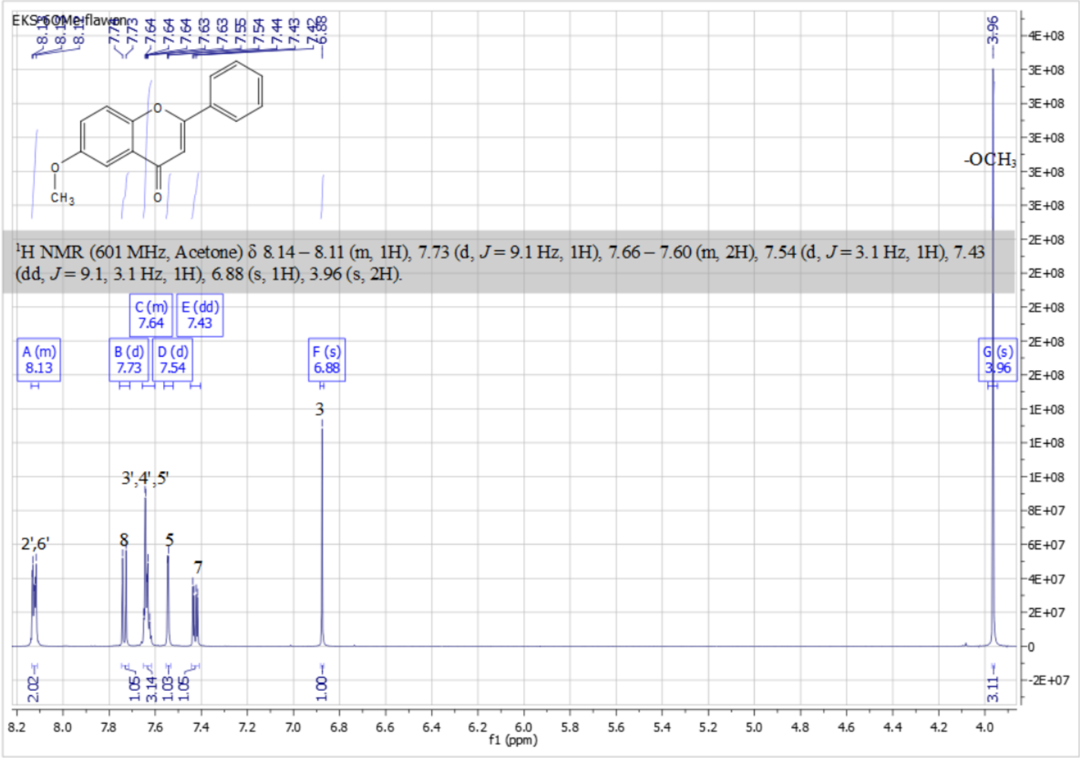

Figure S98. ^{1}H NMR spectrum of 6-methoxyflavone (5) (Acetone-d6, 600 MHz)

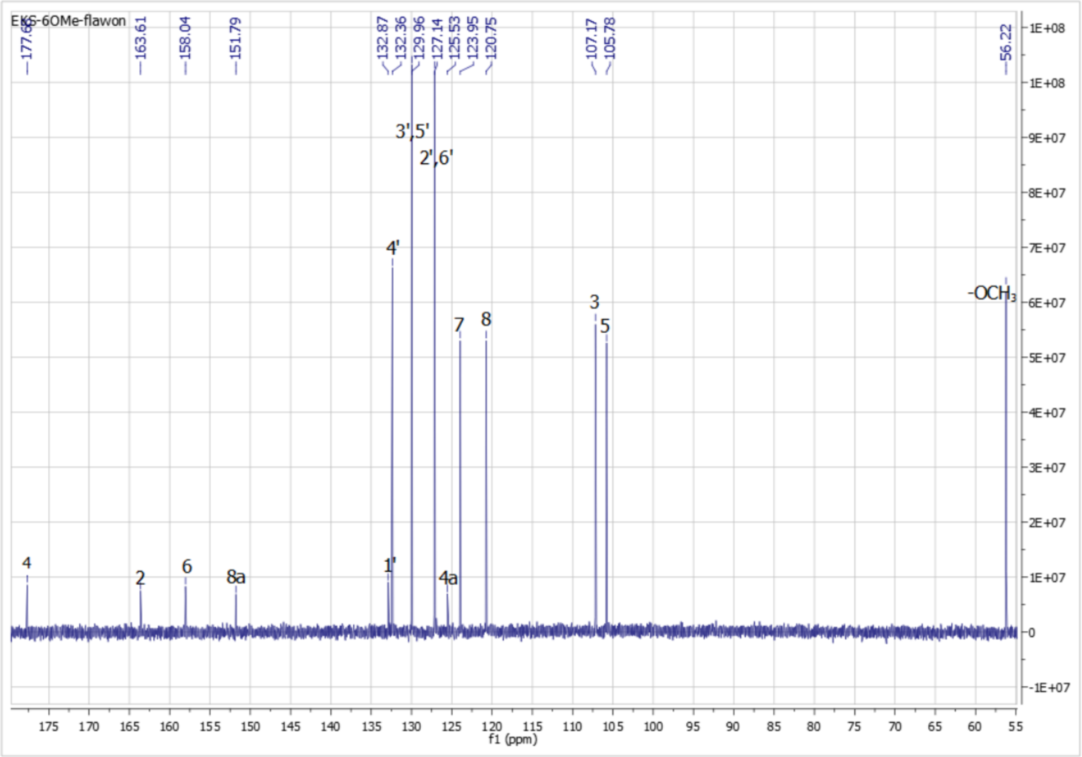

Figure S99. ^{13}C NMR spectrum of 6-methoxyflavone (5) (Acetone-d$_6$, 151 MHz)

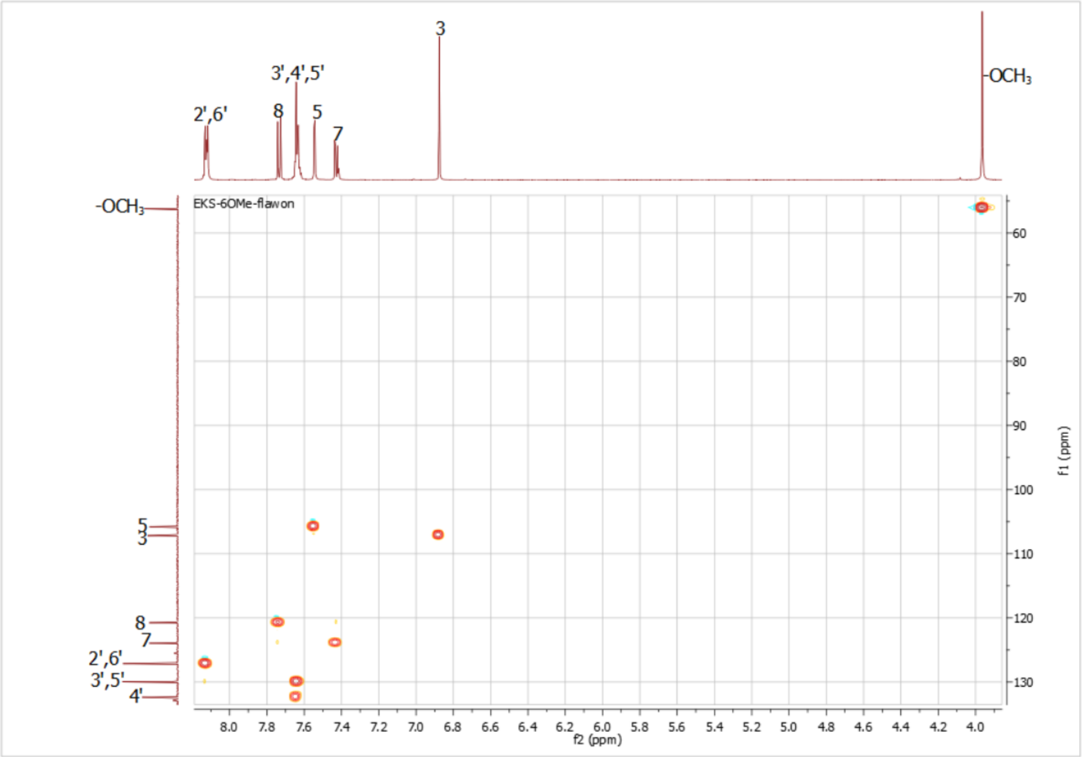

Figure S100. HSQC NMR spectrum of 6-methoxyflavone (5) (Acetone-d$_6$, 151 MHz)

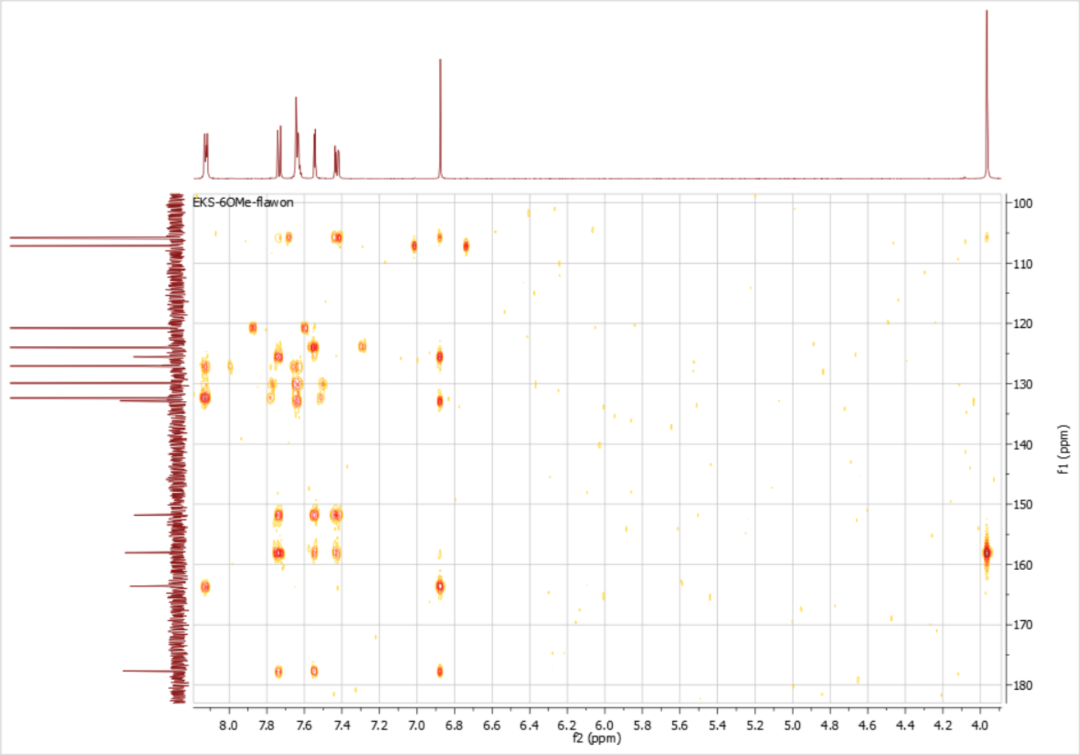

Figure S101. HMBC NMR spectrum of 6-methoxyflavone (5) (Acetone-d6, 151 MHz)

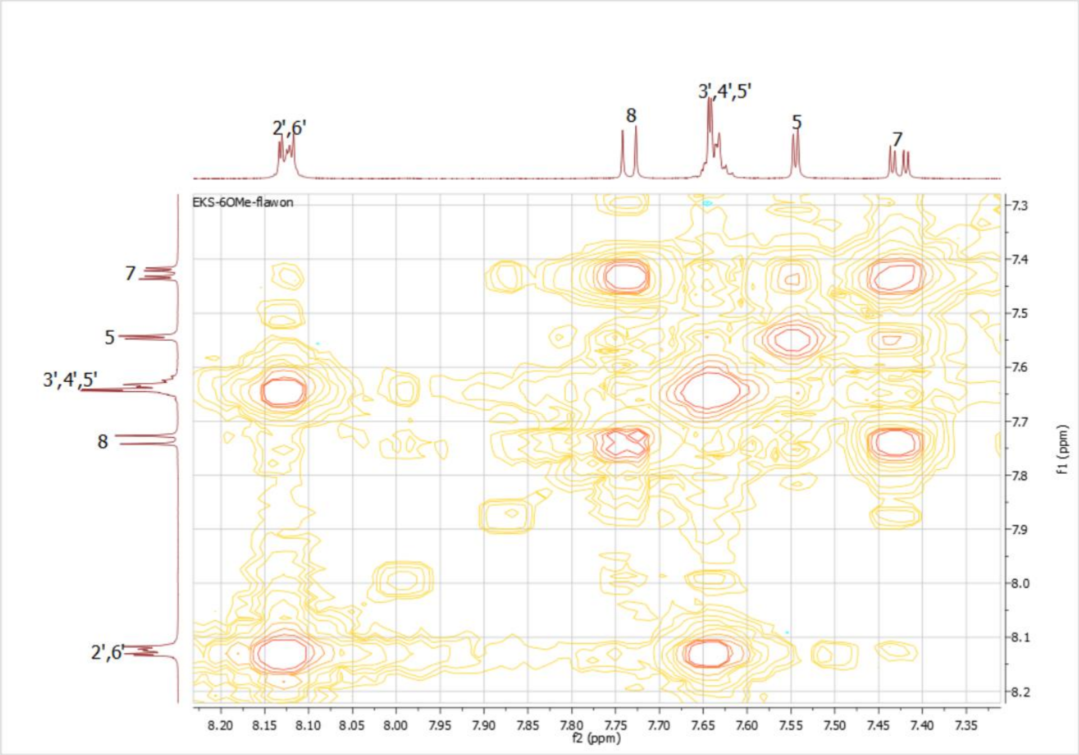

Figure S102. COSY NMR spectrum of 6-methoxyflavone (5) (Acetone-d6, 600 MHz)

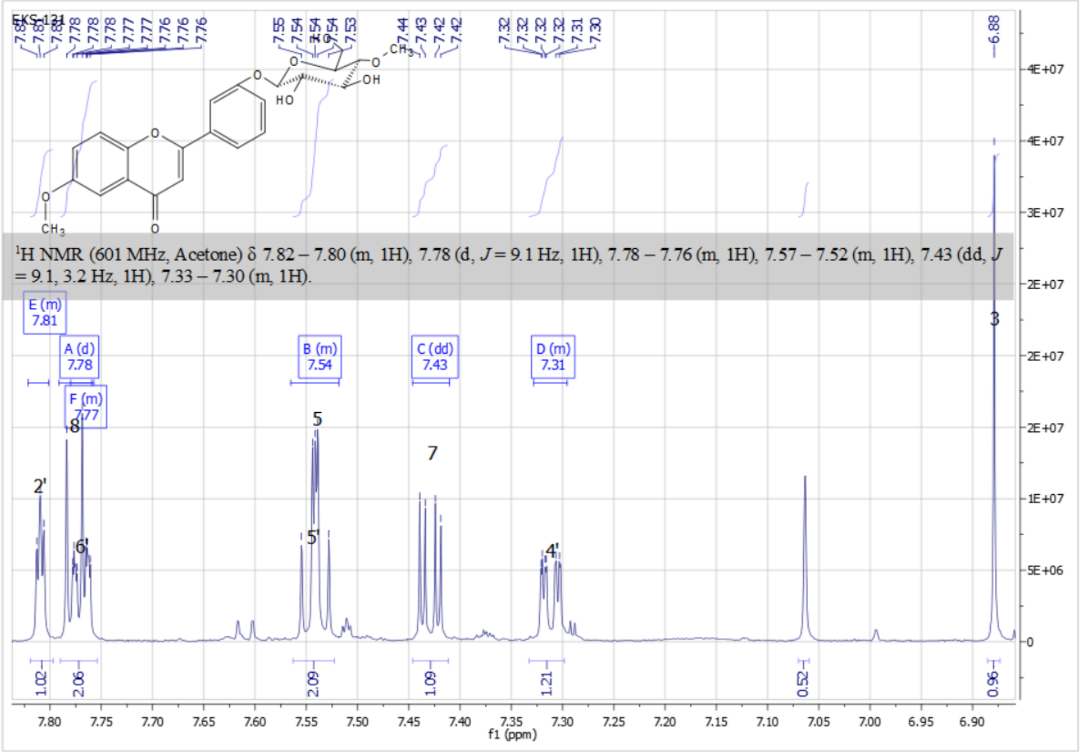

Figure S103. ^{1}H NMR spectrum of 6-methoxyflavone 3′-*O*-β-D-(4″-*O*-methyl)-glucopyranoside (5a) (Acetone-d$_6$, 600 MHz)

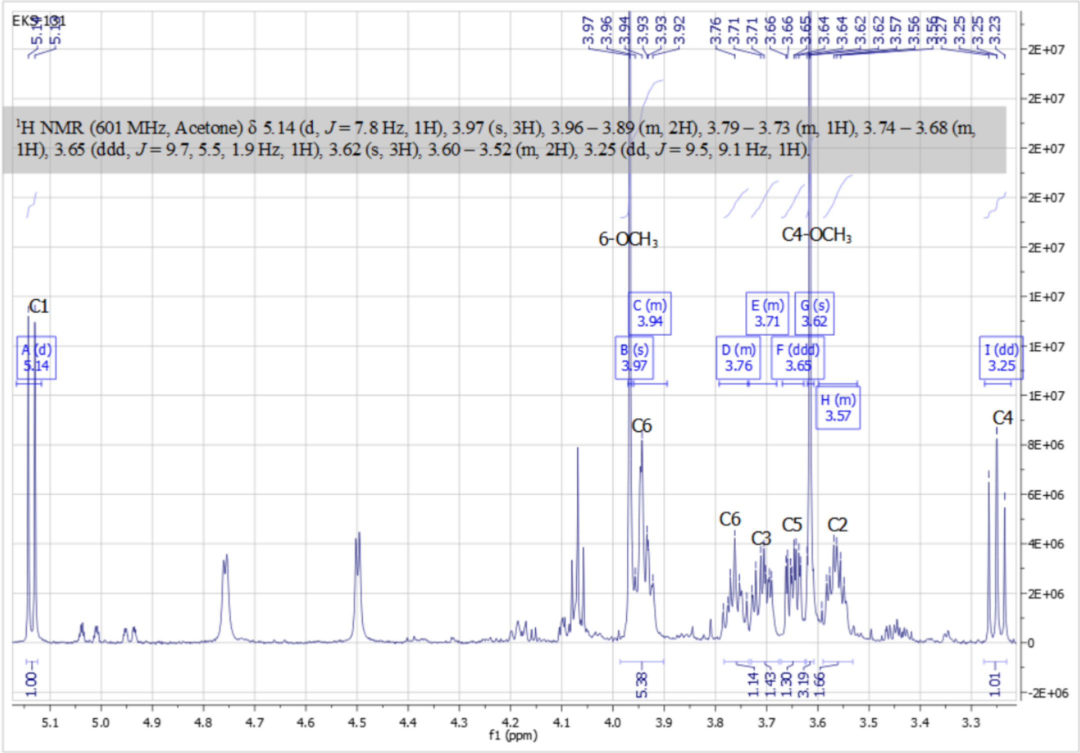

Figure S104. ^{1}H NMR spectrum of 6-methoxyflavone 3′-*O*-β-D-(4″-*O*-methyl)-glucopyranoside (5a) (Acetone-d$_6$, 600 MHz)

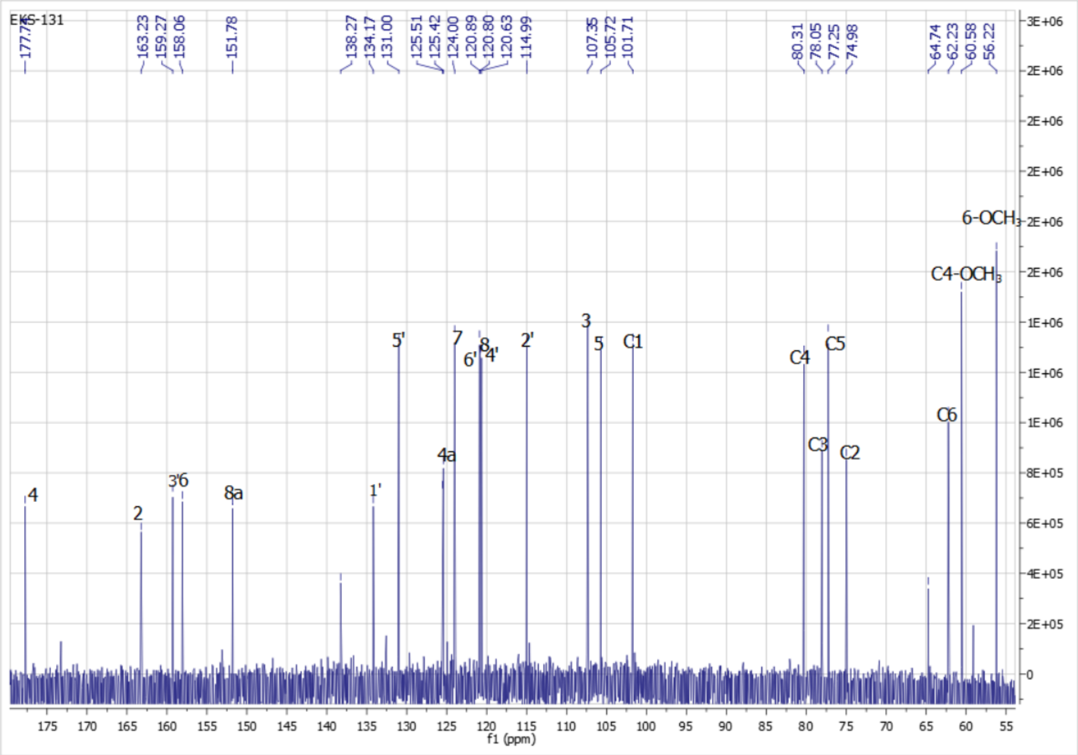

Figure S105. ^{13}C NMR spectrum of 6-methoxyflavone 3′-*O*-β-D-(4″-*O*-methyl)-glucopyranoside (5a) (Acetone-d_6, 151 MHz)

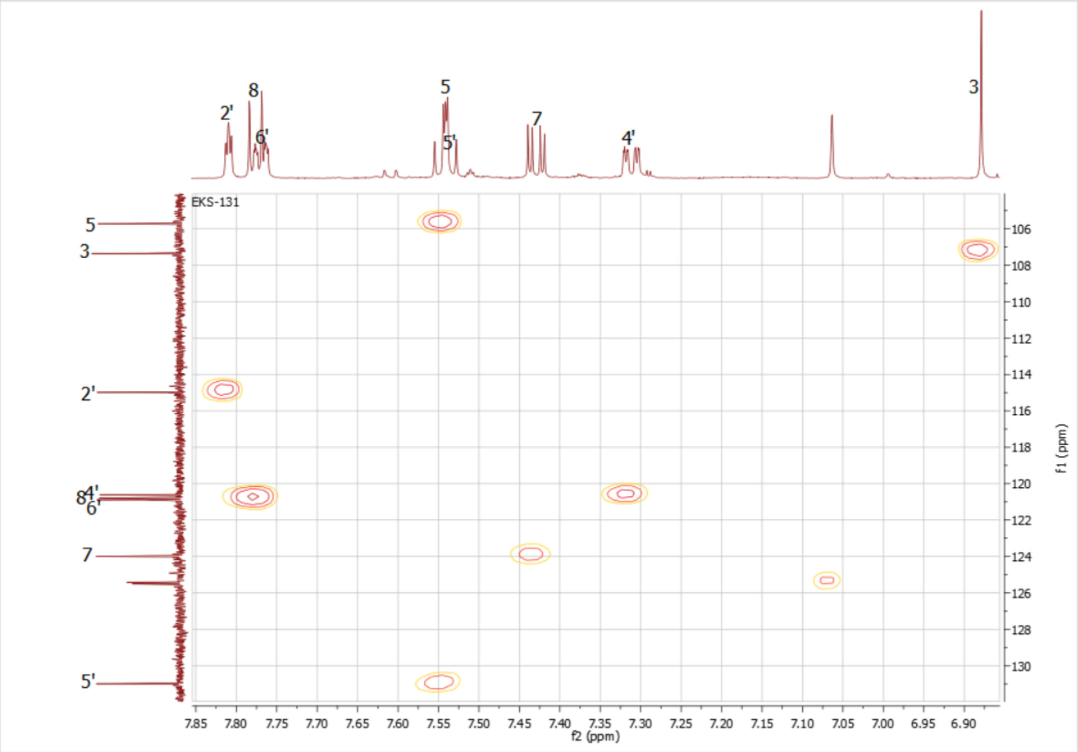

Figure S106. HSQC NMR spectrum of 6-methoxyflavone 3′-*O*-β-D-(4″-*O*-methyl)-glucopyranoside (5a) (Acetone-d_6, 151 MHz)

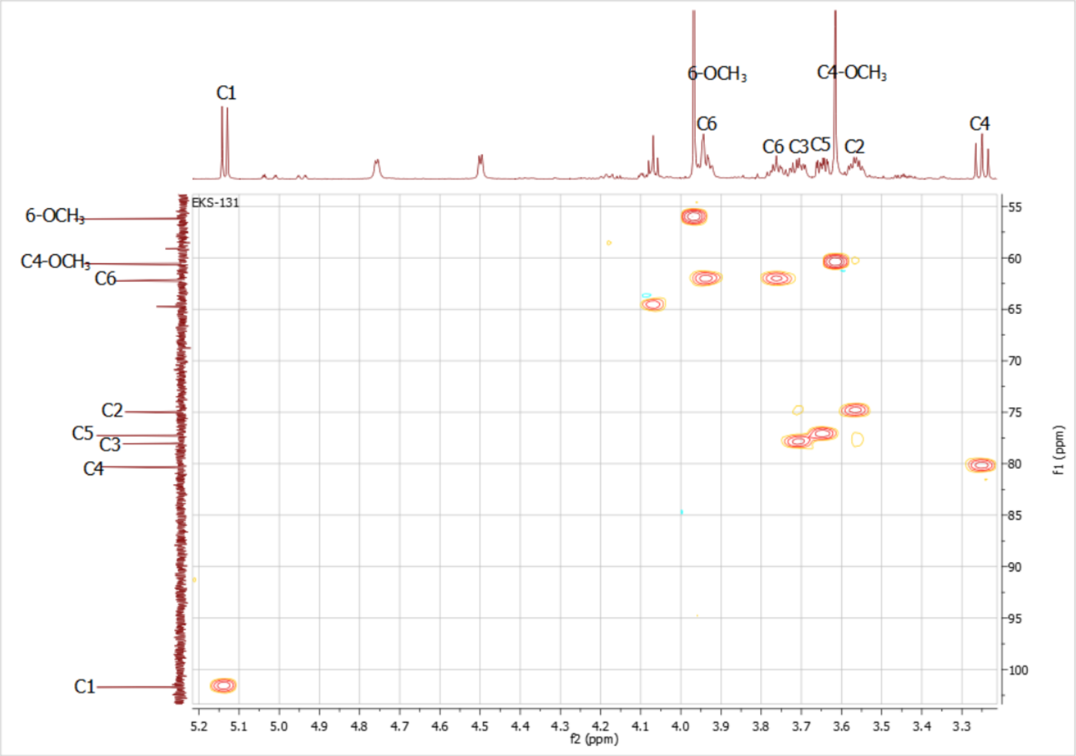

Figure S107. HSQC NMR spectrum of 6-methoxyflavone 3′-*O*-β-D-(4″-*O*-methyl)-glucopyranoside (5a) (Acetone-d₆, 151 MHz)

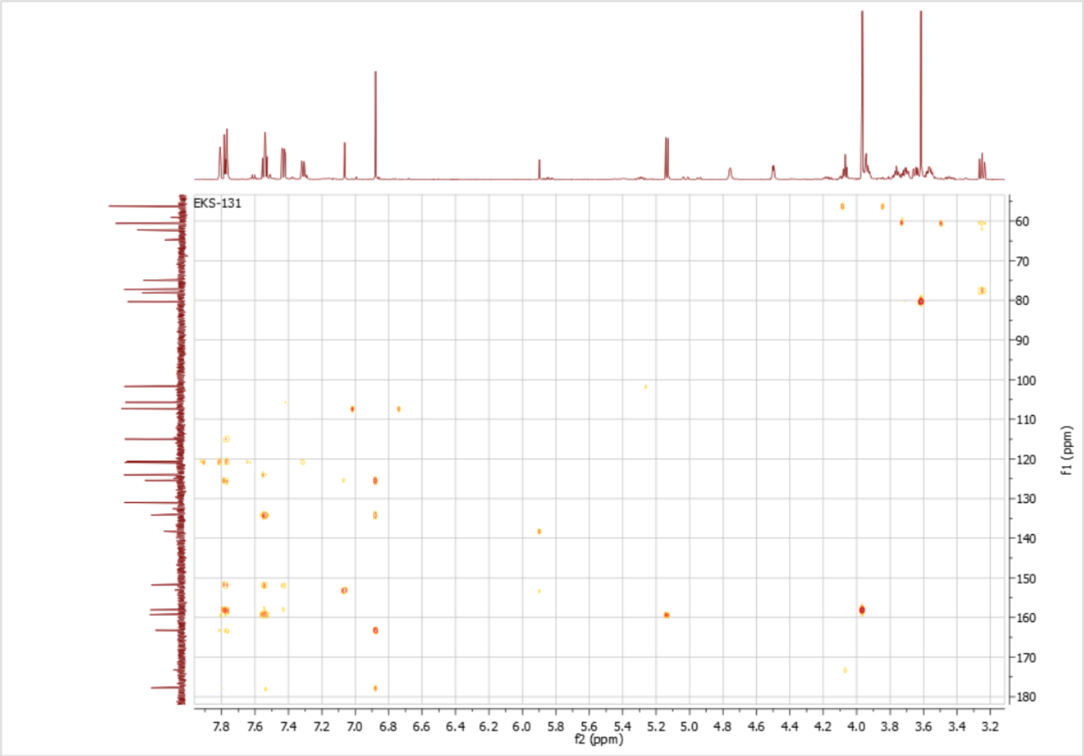

Figure S108. HMBC NMR spectrum of 6-methoxyflavone 3′-*O*-β-D-(4″-*O*-methyl)-glucopyranoside (5a) (Acetone-d₆, 151 MHz)

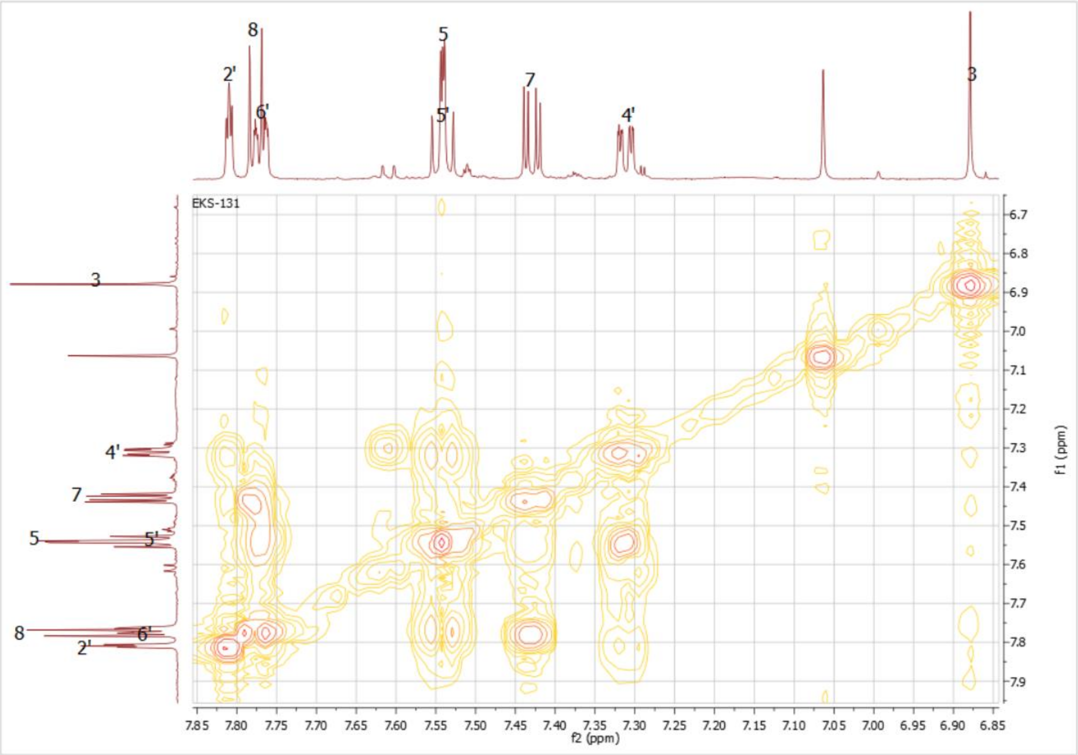

Figure S109. COSY NMR spectrum of 6-methoxyflavone 3′-*O*-β-D-(4′′-*O*-methyl)-glucopyranoside (5a) (Acetone-d$_6$, 600 MHz)

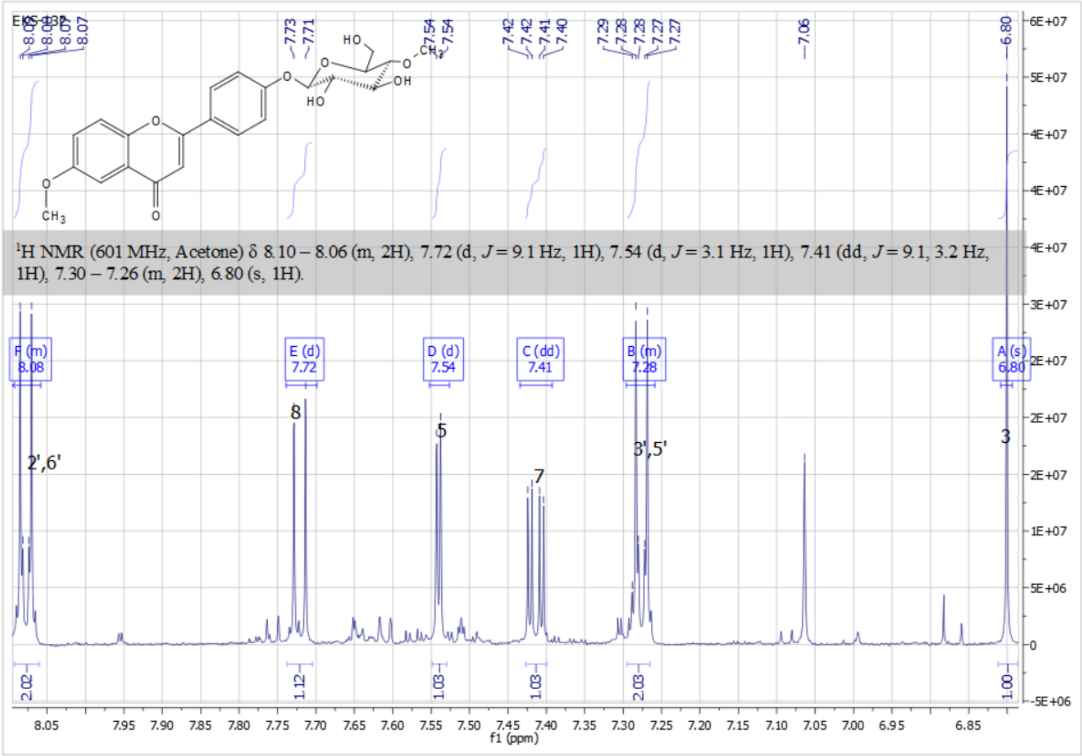

Figure S110. ^{1}H NMR spectrum of 6-methoxyflavone 4′-*O*-β-D-(4′′-*O*-methyl)-glucopyranoside (5b) (Acetone-d$_6$, 600 MHz)

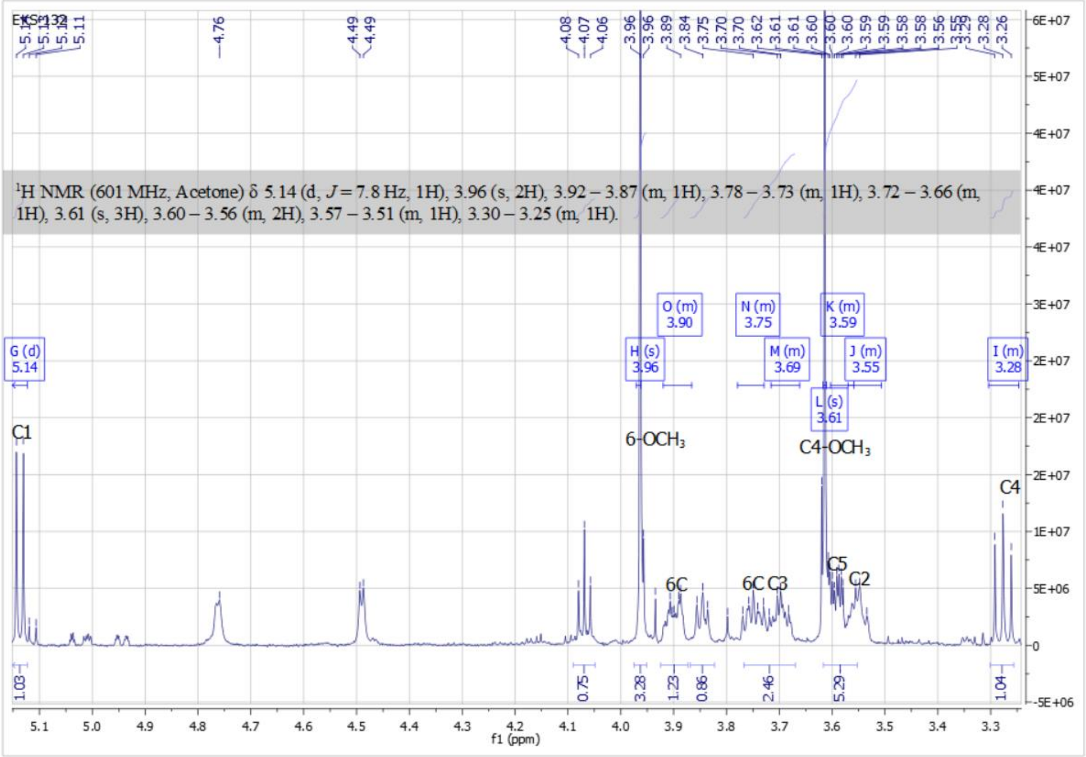

Figure S111. ^{1}H NMR spectrum of 6-methoxyflavone 4′-*O*-β-D-(4″-*O*-methyl)-glucopyranoside (5b) (Acetone-d$_6$, 600 MHz)

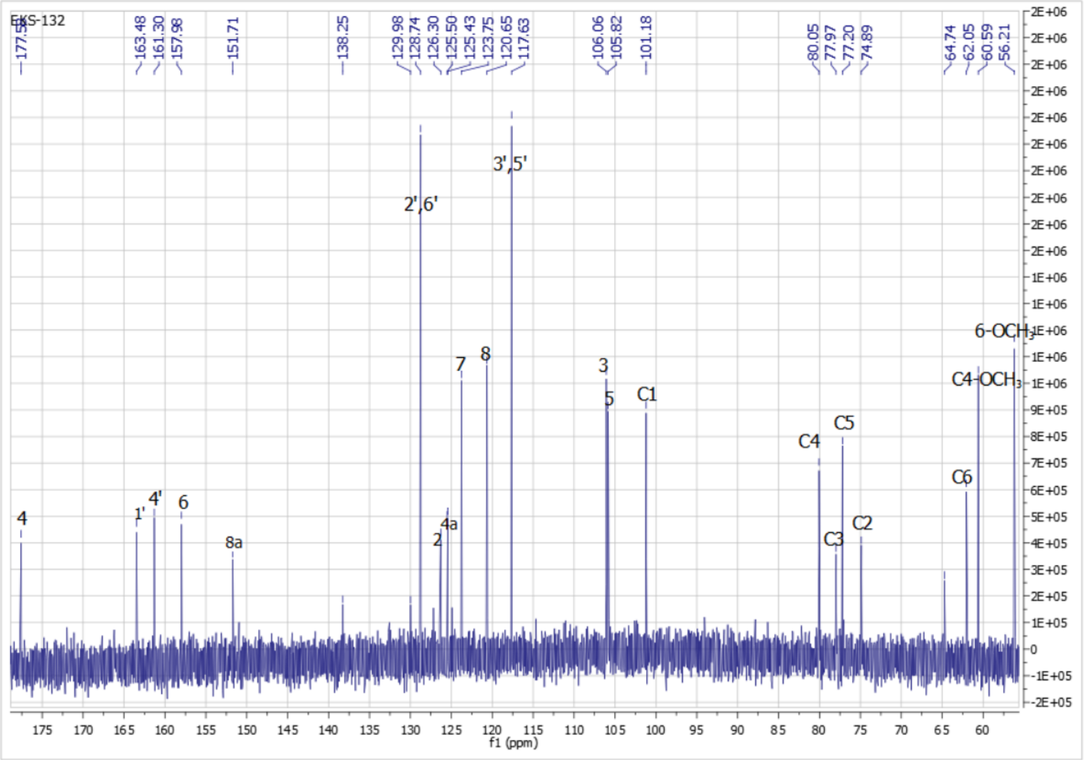

Figure S112. ^{13}C NMR spectrum of 6-methoxyflavone 4′-*O*-β-D-(4″-*O*-methyl)-glucopyranoside (5b) (Acetone-d$_6$, 151 MHz)

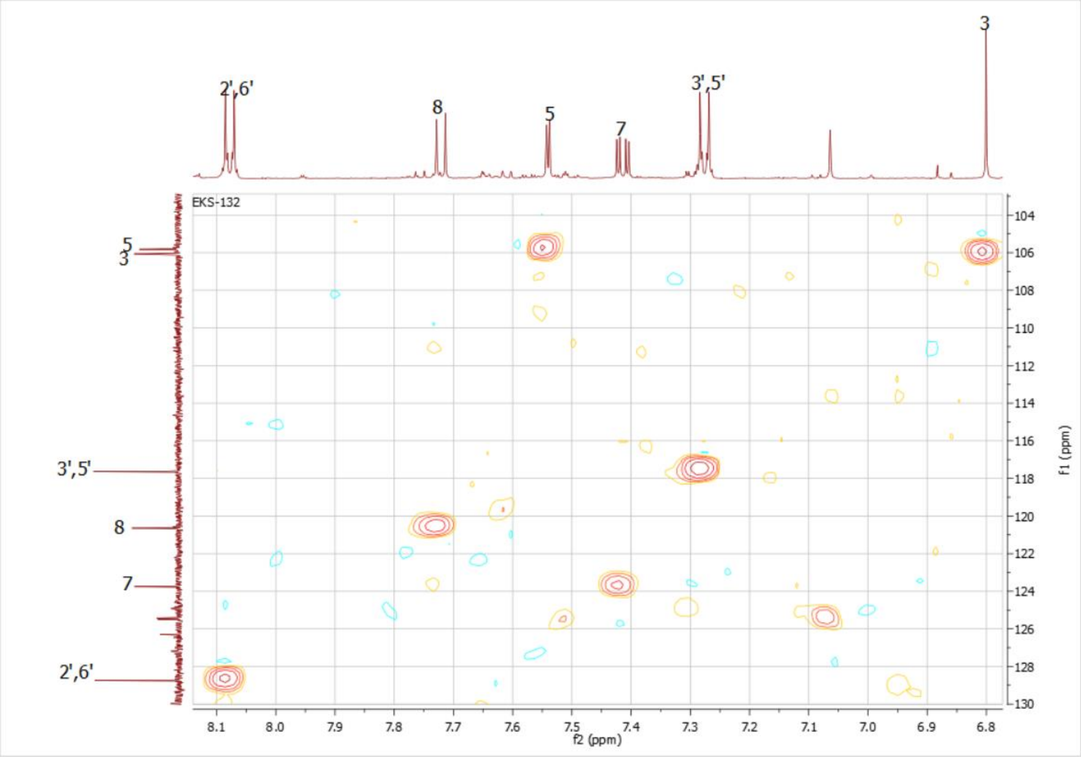

Figure S113. HSQC NMR spectrum of 6-methoxyflavone 4′-*O*-β-D-(4′′-*O*-methyl)-glucopyranoside (5b) (Acetone-d$_6$, 151 MHz)

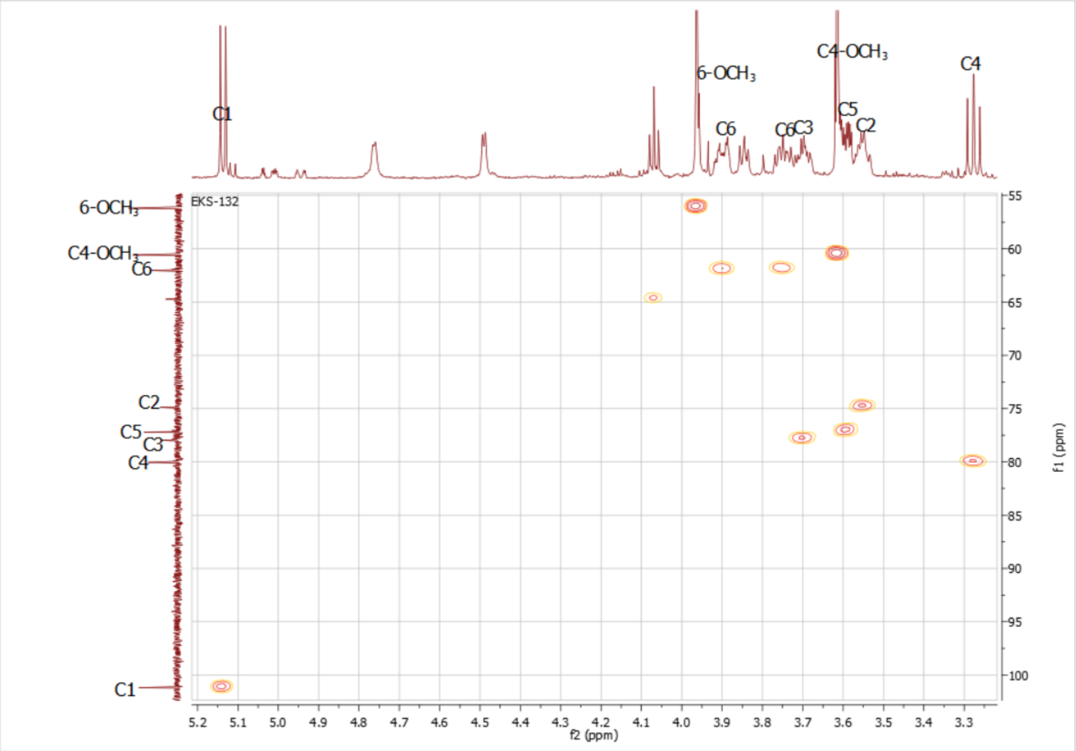

Figure S114. HSQC NMR spectrum of 6-methoxyflavone 4′-*O*-β-D-(4′′-*O*-methyl)-glucopyranoside (5b) (Acetone-d$_6$, 151 MHz)

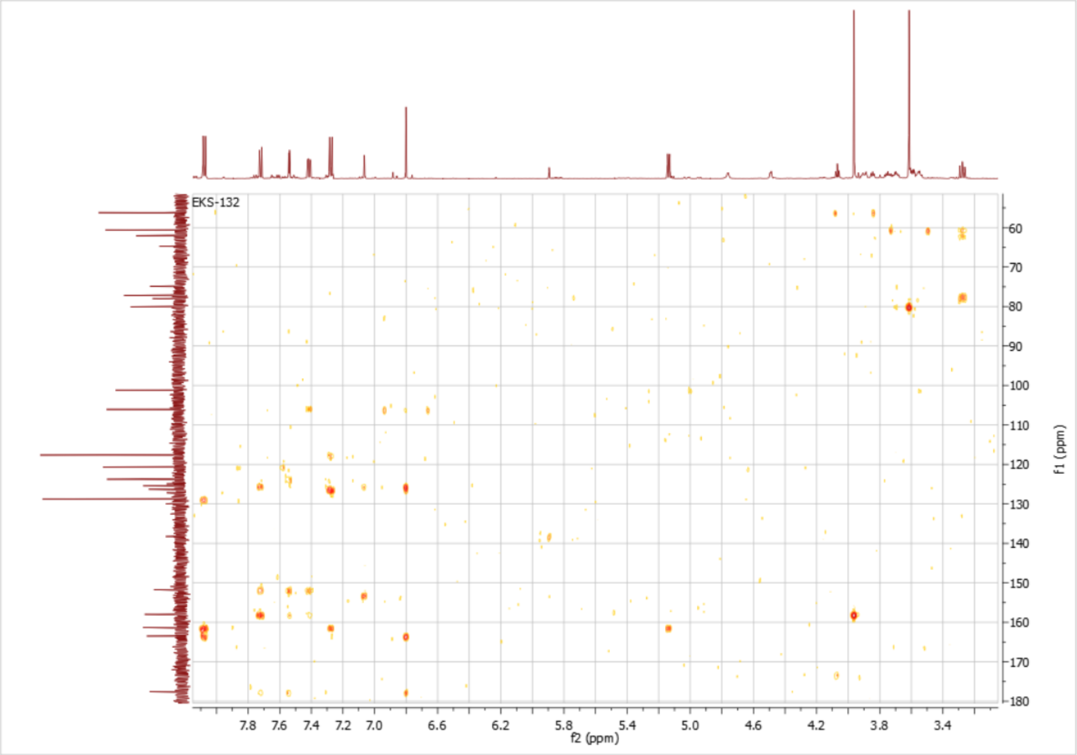

Figure S115. HMBC NMR spectrum of 6-methoxyflavone 4'-*O*-β-D-(4''-*O*-methyl)-glucopyranoside (5b) (Acetone-d_6, 151 MHz)

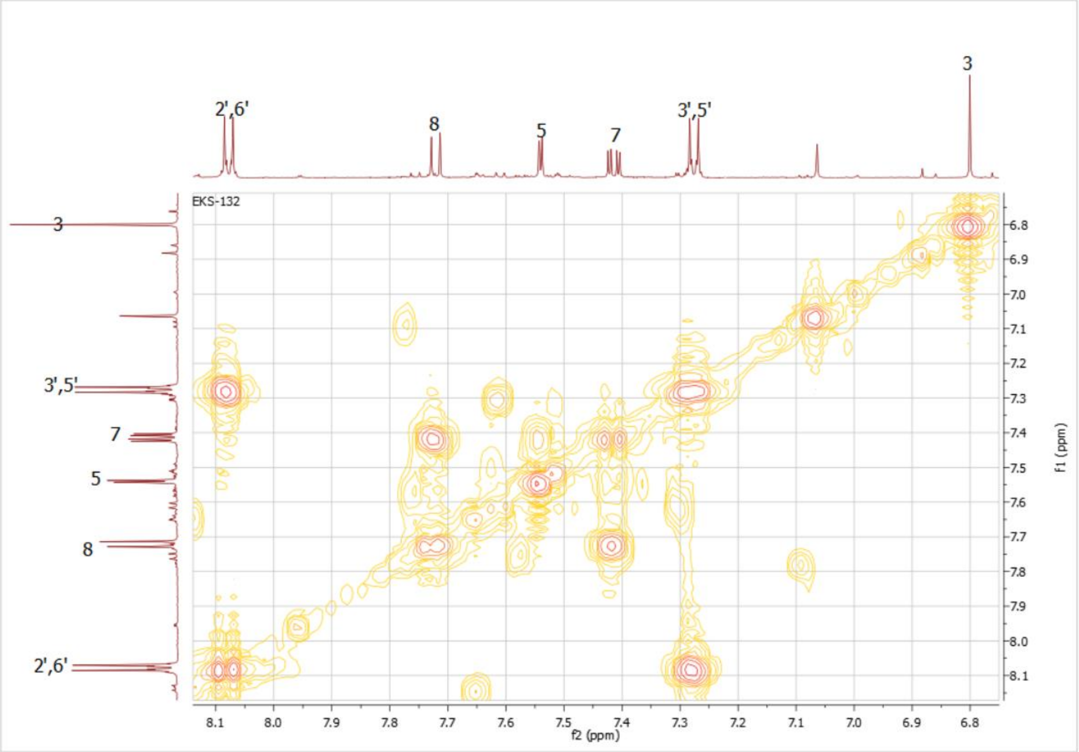

Figure S116. COSY NMR spectrum of 6-methoxyflavone 4'-*O*-β-D-(4''-*O*-methyl)-glucopyranoside (5b) (Acetone-d_6, 600 MHz)

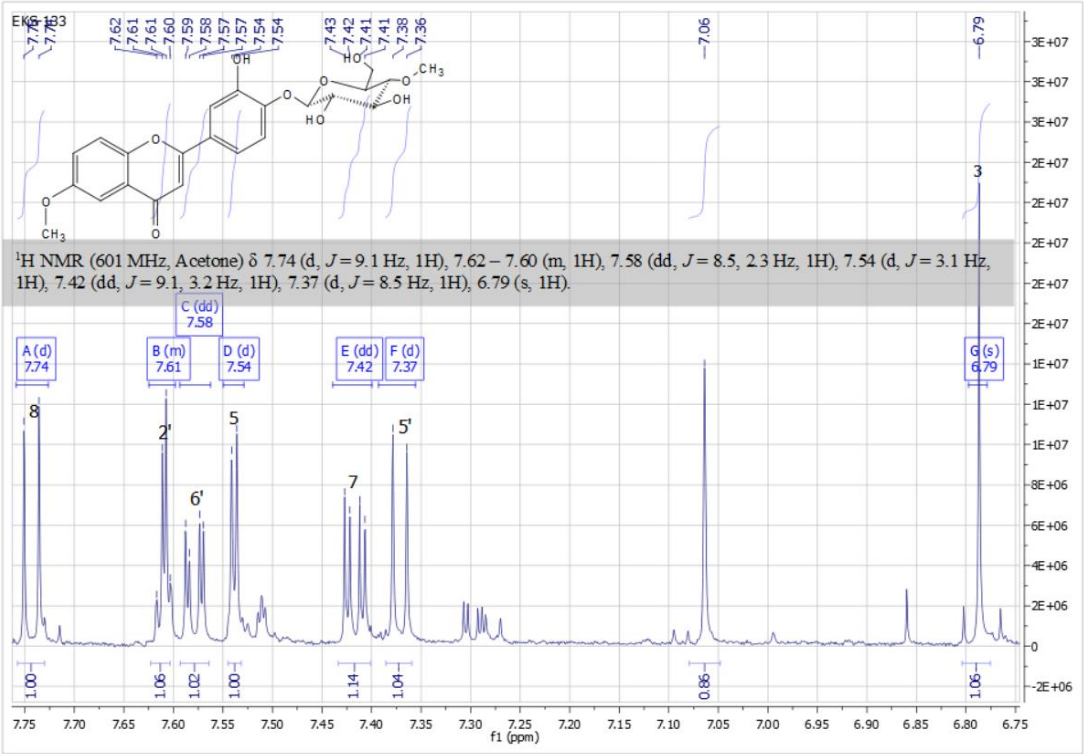

Figure S117. ^{1}H NMR spectrum of 3′-hydroxy-6-methoxyflavone 4′-*O*-β-D-(4″-*O*-methyl)-glucopyranoside (5c) (Acetone-d_6, 600 MHz)

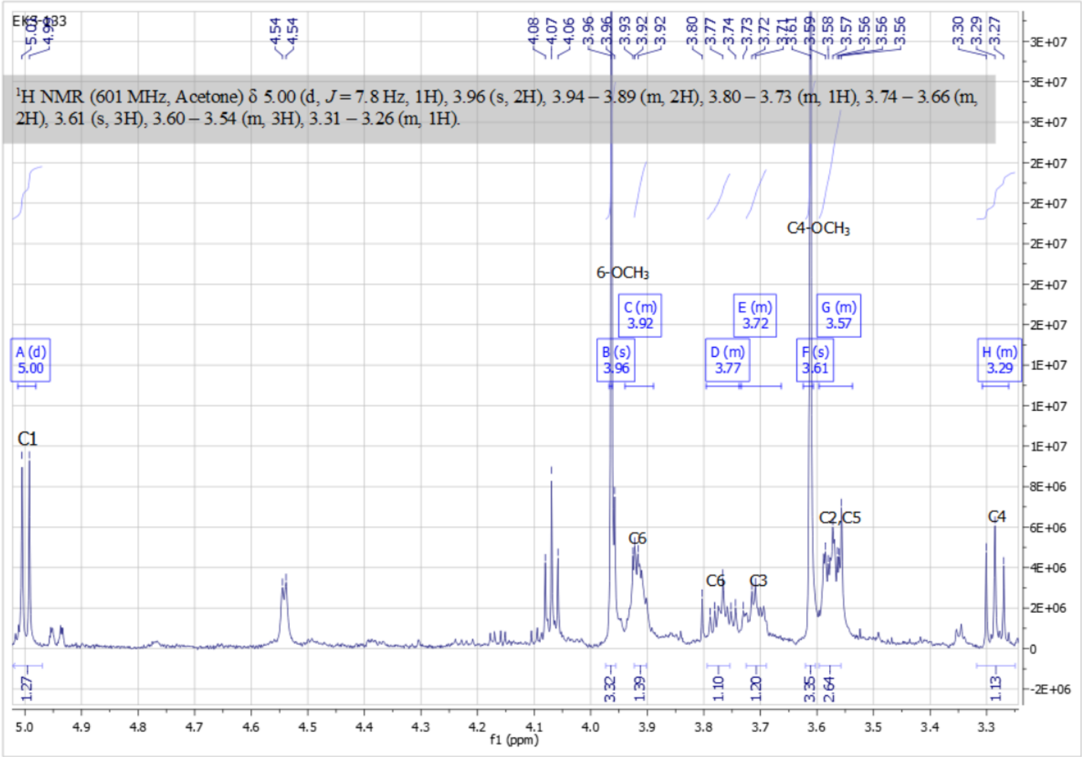

Figure S118. ^{1}H NMR spectrum of 3′-hydroxy-6-methoxyflavone 4′-*O*-β-D-(4″-*O*-methyl)-glucopyranoside (5c) (Acetone-d_6, 600 MHz)

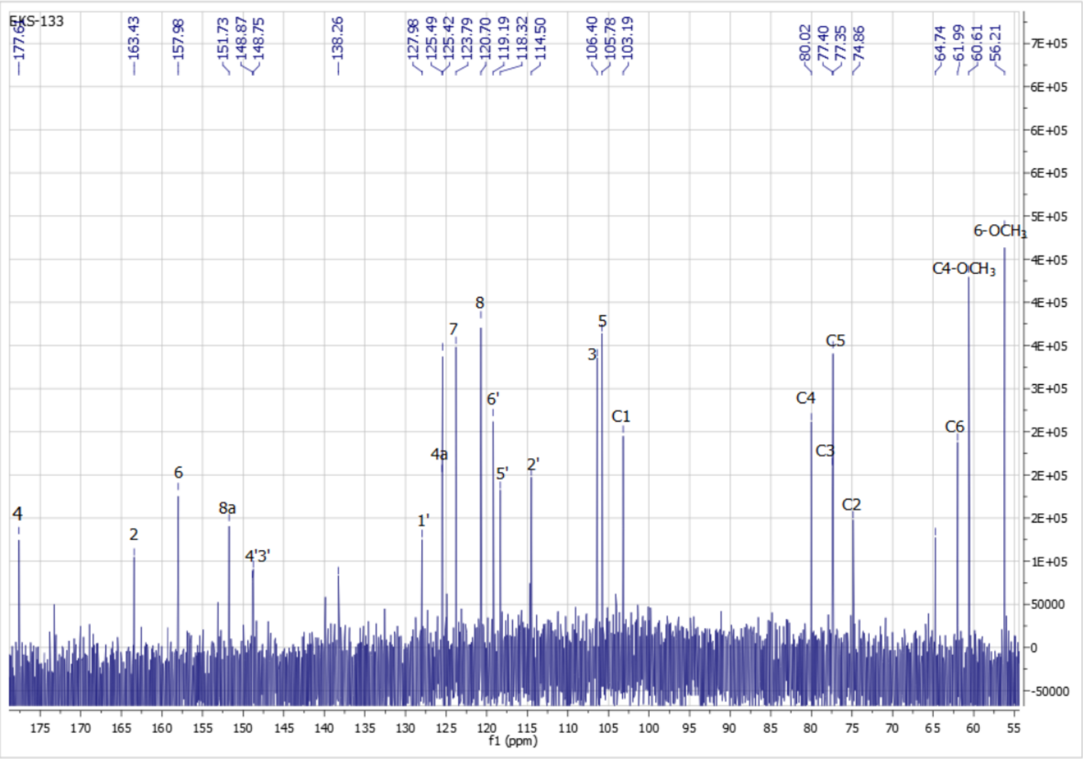

Figure S119. ^{13}C NMR spectrum of 3′-hydroxy-6-methoxyflavone 4′-*O*-β-D-(4″-*O*-methyl)-glucopyranoside (5c) (Acetone-d$_6$, 151 MHz)

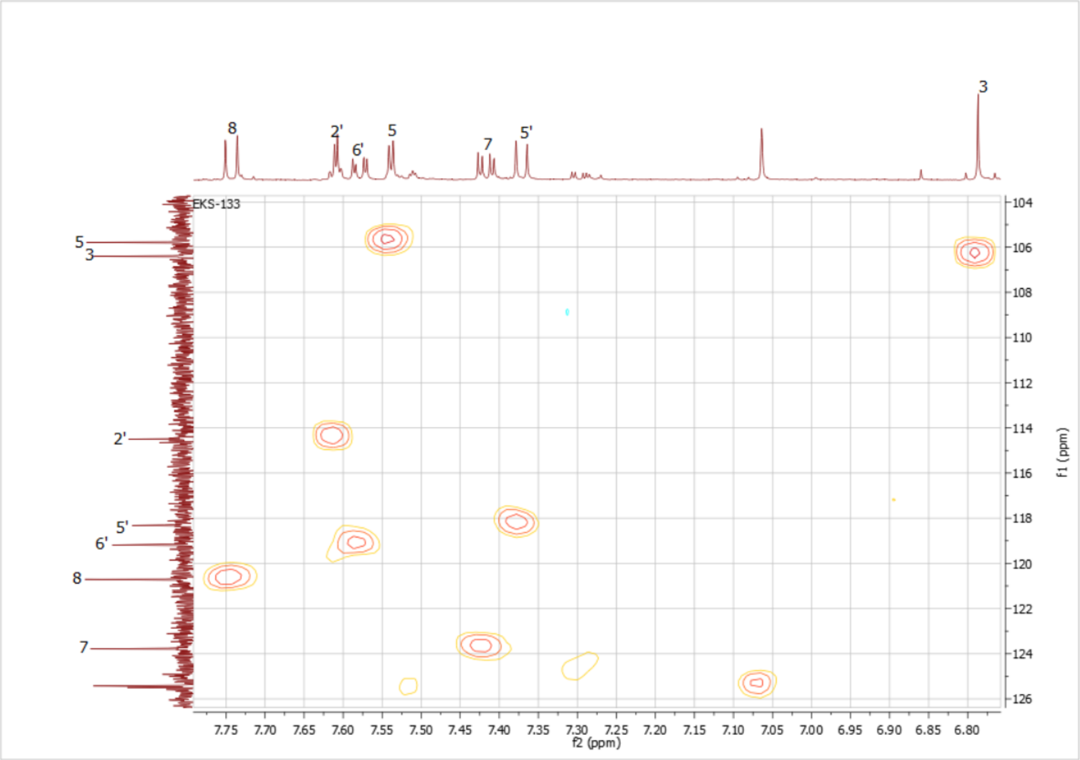

Figure S120. HSQC NMR spectrum of 3′-hydroxy-6-methoxyflavone 4′-*O*-β-D-(4″-*O*-methyl)-glucopyranoside (5c) (Acetone-d$_6$, 151 MHz)

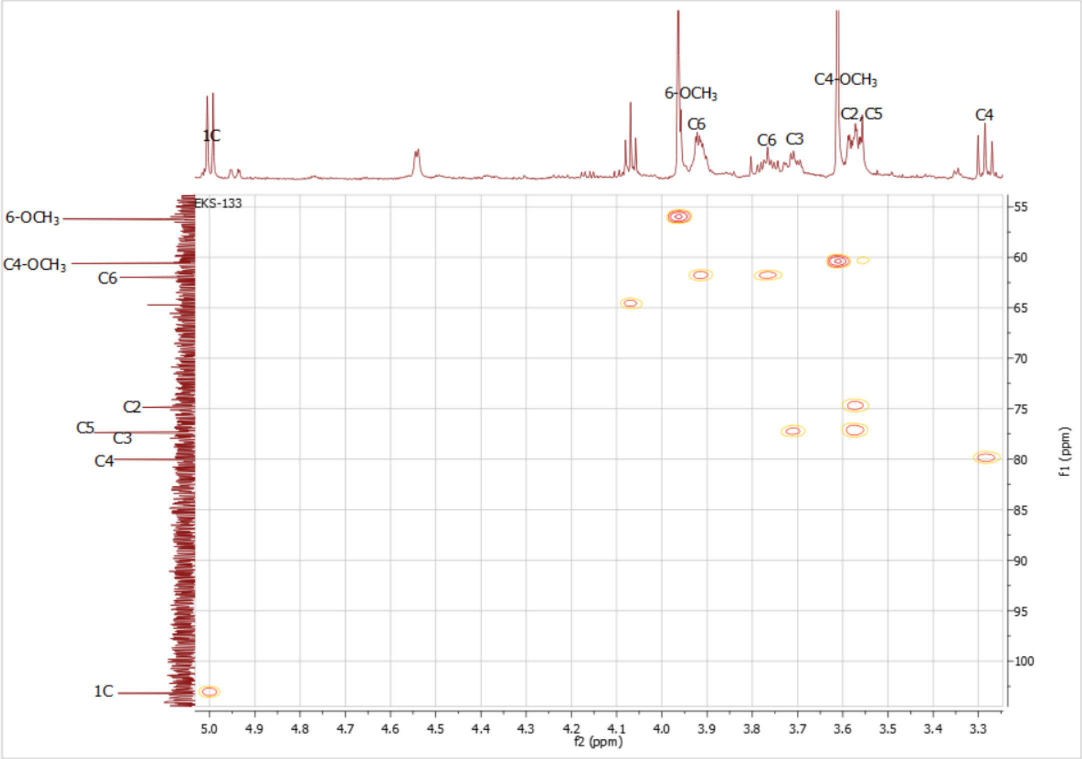

Figure S121. HSQC NMR spectrum of 3′-hydroxy-6-methoxyflavone 4′-*O*-β-D-(4″-*O*-methyl)-glucopyranoside (5c) (Acetone-d6, 151 MHz)

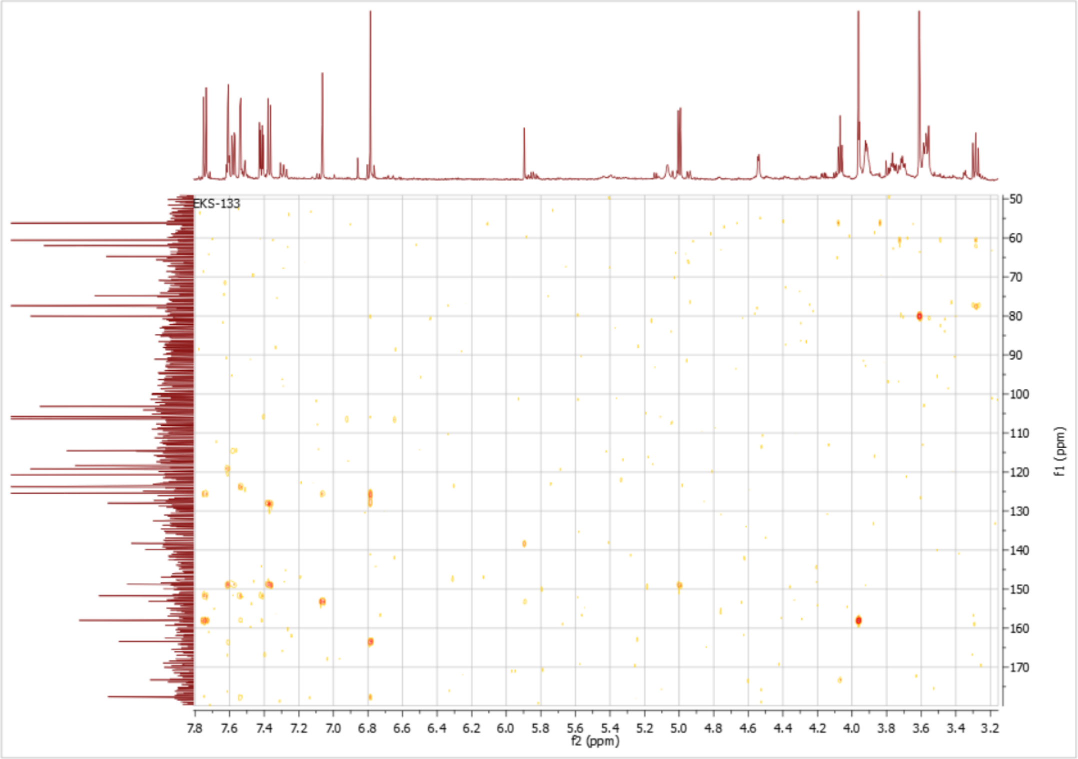

Figure S122. HMBC NMR spectrum of 3′-hydroxy-6-methoxyflavone 4′-*O*-β-D-(4″-*O*-methyl)-glucopyranoside (5c) (Acetone-d6, 151 MHz)

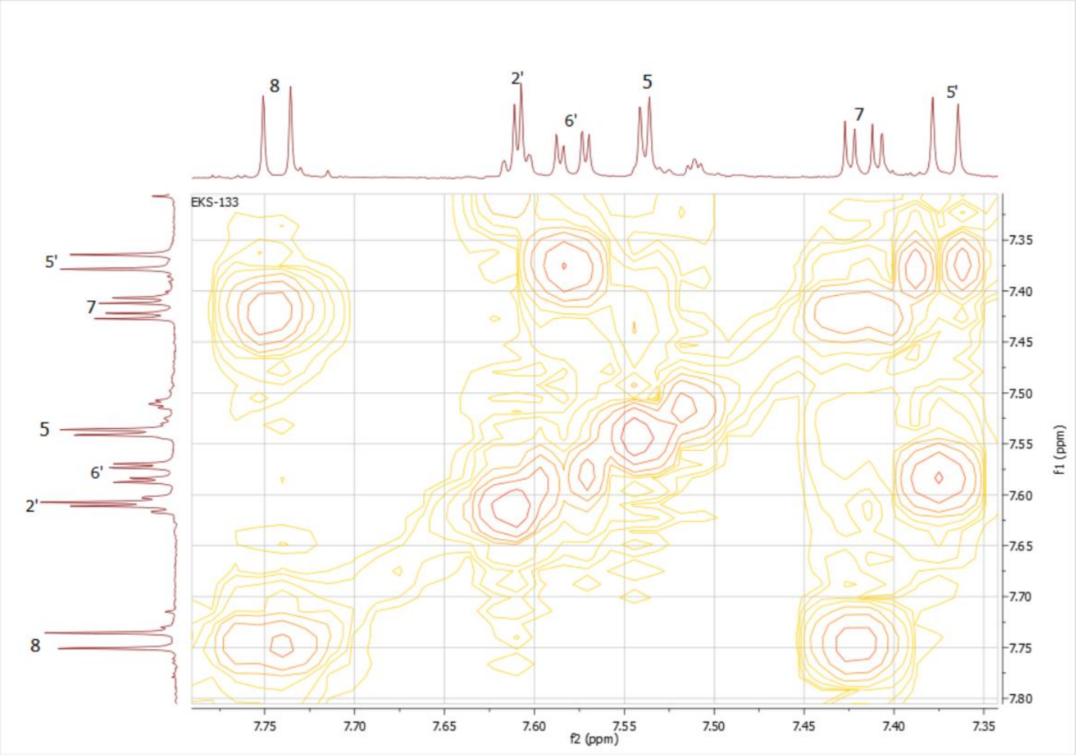

Figure S123. COSY NMR spectrum of 3′-hydroxy-6-methoxyflavone 4′-*O*-β-D-(4′′-*O*-methyl)-glucopyranoside (5c) (Acetone-d_6, 600 MHz)
